# Data-Centric Heterogeneous Catalysis: Identifying Rules and Materials Genes of Alkane Selective Oxidation

**DOI:** 10.1021/jacs.2c11117

**Published:** 2023-02-06

**Authors:** Lucas Foppa, Frederik Rüther, Michael Geske, Gregor Koch, Frank Girgsdies, Pierre Kube, Spencer J. Carey, Michael Hävecker, Olaf Timpe, Andrey V. Tarasov, Matthias Scheffler, Frank Rosowski, Robert Schlögl, Annette Trunschke

**Affiliations:** † The NOMAD Laboratory at the Fritz-Haber-Institut of the Max-Planck-Gesellschaft and IRIS-Adlershof of the Humboldt-Universität zu Berlin, Faradayweg 4-6, D-14195 Berlin, Germany; ‡ BasCat - UniCat BASF JointLab, Hardenbergstraße 36, D-10623 Berlin, Germany; § Department of Inorganic Chemistry, Fritz-Haber-Institut of the Max-Planck-Gesellschaft, Faradayweg 4-6, D-14195 Berlin, Germany; ∥ Max Planck Institute for Chemical Energy Conversion, 45470 Mülheim, Germany; ⊥ BASF SE, Catalysis Research, Carl-Bosch-Straße 38, D-67065 Ludwigshafen, Germany

## Abstract

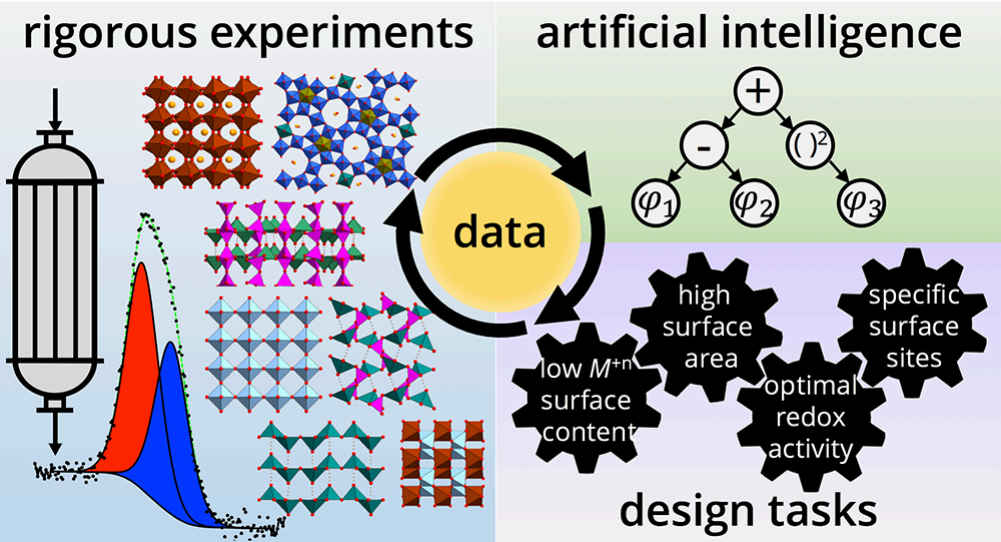

Artificial intelligence (AI) can accelerate catalyst design by identifying key physicochemical descriptive parameters correlated with the underlying processes triggering, favoring, or hindering the performance. In analogy to genes in biology, these parameters might be called “materials genes” of heterogeneous catalysis. However, widely used AI methods require big data, and only the smallest part of the available data meets the quality requirement for data-efficient AI. Here, we use rigorous experimental procedures, designed to consistently take into account the kinetics of the catalyst active states formation, to measure 55 physicochemical parameters as well as the reactivity of 12 catalysts toward ethane, propane, and *n*-butane oxidation reactions. These materials are based on vanadium or manganese redox-active elements and present diverse phase compositions, crystallinities, and catalytic behaviors. By applying the sure-independence-screening-and-sparsifying-operator symbolic-regression approach to the consistent data set, we identify nonlinear property–function relationships depending on several key parameters and reflecting the intricate interplay of processes that govern the formation of olefins and oxygenates: local transport, site isolation, surface redox activity, adsorption, and the material dynamical restructuring under reaction conditions. These processes are captured by parameters derived from N_2_ adsorption, X-ray photoelectron spectroscopy (XPS), and near-ambient-pressure in situ XPS. The data-centric approach indicates the most relevant characterization techniques to be used for catalyst design and provides “rules” on how the catalyst properties may be tuned in order to achieve the desired performance.

## Introduction

Heterogeneous catalysis is a key technology that enables the production of fuels and synthetic materials and that addresses pollution control, greenhouse gas emissions, and dwindling resources. More efficient catalysts are needed to render desired energy conversion and storage in the required large scales, to close the carbon cycle in the use of fossil raw materials, and to apply alternative carbon-free molecules such as ammonia for storage of energy and matter.^1−4^ However, the description and modeling of catalysis and the design of catalysts are challenged by the intricate interplay of numerous, not fully understood underlying processes that govern the materials’ function, such as the surface bond-breaking and -forming reactions, the restructuring of the material under the catalytic reaction environment, and the transport of molecules and energy. In particular, the solid-state chemistry of the material is strongly coupled with the chemistry of the catalytic reaction, since the stability of surface and bulk phases under reaction conditions is determined by the fluctuating chemical potential, which in turn depends on the kinetics of the elementary reaction steps in the usually complex reaction network. Thus, reversible catalyst dynamics characterizes stationary operation, and the states of the material which are relevant for the conversion of reactants into products are often unknown.^5−12^ Due to such complexity, the theoretical first-principles atomistic modeling of the full catalytic progression is extremely difficult,^6,13−15^ and the experimental design of catalysts is hindered by the overwhelming number of materials- and reaction-related physicochemical parameters that could be tuned to achieve improved performance.

AI has been employed to uncover correlations, patterns, and anomalies in materials science and catalysis, accelerating the design of improved or even novel materials.^16−29^ However, AI and machine learning typically need a large amount of data and only a few AI approaches are applicable to a number of materials that can be synthesized and characterized in detail by experiments (e.g., <10^2^). For such data-efficient approaches, it is then crucial that the data are of high quality. Nevertheless, only the smallest part of the available heterogeneous catalysis data meets the requirements for data-efficient AI. This is due to several reasons. First, the kinetics of the formation of the active states of a catalyst is often neglected while designing the experiments for measuring the catalyst properties and performance. Thus, the same system may run on different paths depending on the workflow of the experiment, generating different active states and leading to inconsistent data, which compromises the reproducibility. Second, most of the systematic studies focus on specific chemically related materials and reactions, and negative results are often omitted. Such subjective bias and the lack of diversity in terms of materials and process parameters (e.g., good as well as bad catalysts and a wide range of reaction conditions) prevent the derivation of general property–function relationships. Finally, incomplete reporting and lack of metadata also make catalysis-research data difficult to reuse.

To overcome these challenges, it is becoming increasingly accepted in catalysis research that the application of rigorous protocols for experiments is necessary.^16,30−35^ We have recently established standardized and detailed procedures for obtaining and reporting heterogeneous catalysis research data documented in “experimental handbooks.”^15^ Crucially, these “clean experiments” are designed to consistently take into account the dynamic nature of the catalyst while generating catalyst samples and measuring their properties and performance. The symbolic-regression sure-independence-screening-and sparsifying-operator (SISSO) AI analysis^36,37^ of the so-generated data resulted in the identification of correlations as interpretable, typically nonlinear analytical expressions of the most relevant physicochemical parameters. These descriptive parameters reflect the processes triggering, favoring, or hindering the reactivity toward propane oxidation as a selected example of a complex catalytic reaction at the gas–solid interface.^38^ In analogy to genes in biology, these parameters might be called “materials genes” of heterogeneous catalysis, since they describe the catalyst function similarly as genes in biology relate, for instance, to the color of the eyes or to health issues, that is, they capture complex relationships, but they do not provide the full understanding of the underlying processes. The analytical expressions, in turn, might be seen as “rules” for catalyst design because they indicate how the materials properties might be tuned in order to improve the performance.

**Figure 1:**
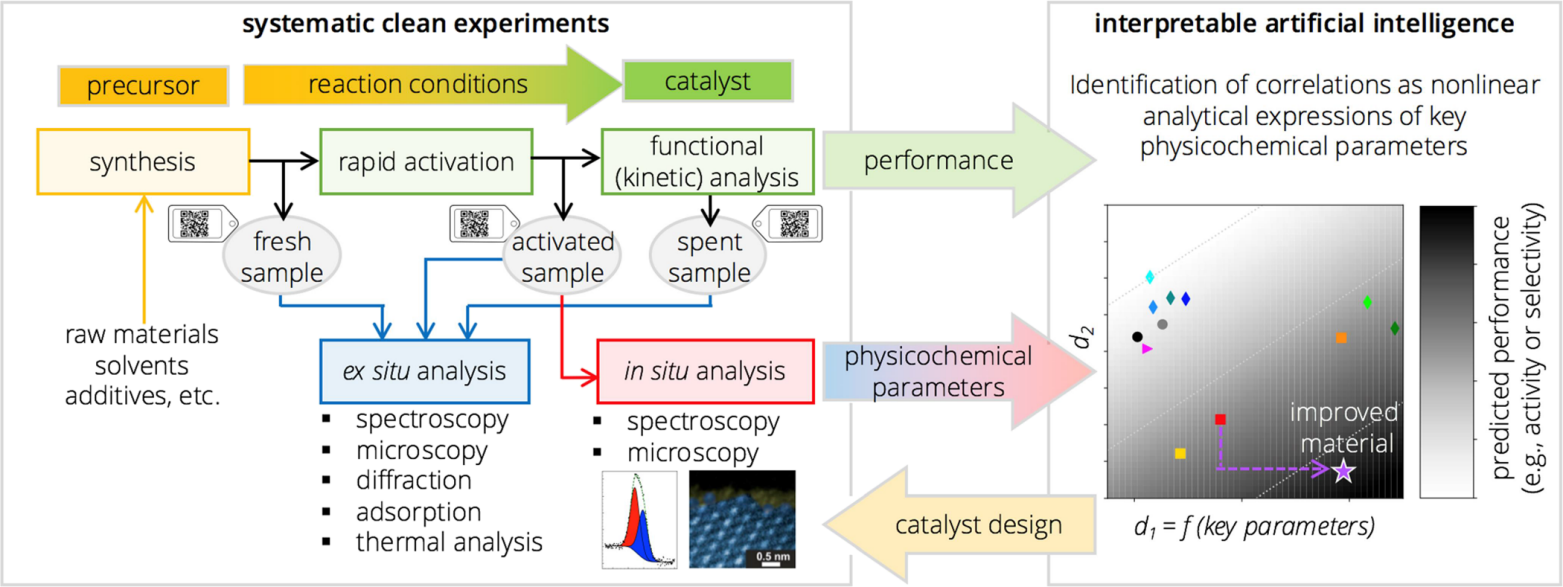
Kinetics makes the difference: systematic “clean experiments” designed to capture the kinetics of the formation of the catalyst active states under reaction conditions are used to generate a consistent and detailed data set, which is then analyzed via the sure-independence-screening-and-sparsifying-operator (SISSO) artificial intelligence (AI) approach in order to uncover the key physicochemical parameters describing the performance. After the synthesis, the prepared materials are subjected to a rapid activation process before they are tested in catalysis. Fresh, activated, and spent samples receive a unique identification number (per synthesis batch) and are fully characterized by ex situ as well as in situ techniques. The correlations uncovered by AI can then be used to guide catalyst design. In the figure, *d*_1_ and *d*_2_ correspond to the descriptor components and they are functions of the key parameters.

To demonstrate the universality of this strategy, we apply here the clean-data-centric approach to identify property–function relationships, simultaneously describing the performance in ethane (C_2_), propane (C_3_), and *n*-butane (C_4_) selective oxidation reactions (Figure 1). Our analysis is based on 12 vanadium- or manganese-based catalysts, which were extensively characterized and present diverse physical properties and catalytic behaviors. We show that the combination of clean data and AI is a prerequisite for the identification of property–function relationships, since these relationships involve multiple parameters related in a nonlinear fashion. The key physicochemical parameters identified not only characterize the catalyst under thermodynamic standard conditions where phases are defined, but also reflect the properties of the materials under the conditions applied at each reaction, as captured by in situ spectroscopy characterization data. This result highlights that the crystal structure and translational repetitive arrangement of atoms in the surface, used in conventional catalyst design approaches and theoretical models, are insufficient to describe selective oxidation catalysis. The data-centric approach accelerates catalyst design, while highlighting the underlying processes.

## Alkane Selective Oxidation

The oxidation of short-chain alkanes with molecular oxygen was chosen as a class of chemical reactions in this study because they involve complex transformations that lead to a variety of products. The selective formation of certain desired compounds requires chemically and structurally sophisticated catalysts.^39^ The particular difficulty is to reach a sufficient yield of valuable products such as olefins or unsaturated oxygenates, that is, to achieve high selectivity of these products at high conversion, while avoiding total oxidation to CO_2_. Numerous studies in the literature and in our own laboratories have investigated these reactions.^40−65^ However, very diverse reaction conditions have been applied, as shown by the literature survey presented in Table S1. This makes a direct comparison of the catalysts impossible and potentially systematic trends not apparent. In general, only catalysts that are structurally related are compared for one given reaction. For example, the influence of the chemical composition of phase-pure polycrystalline (Mo,V,Te,Nb) mixed oxides with the “M1” crystal structure on the catalytic properties in propane oxidation was systematically investigated.^40,66^ Similarly, different vanadium phosphate phases^60^ or vanadium pentoxide and vanadyl pyrophosphate (VPP)^53^ were compared in the oxidation of *n*-butane. Other studies investigate multiple reactions, but focus on one single material. For instance, the oxidation of ethane, propane, and *n*-butane was compared on both M1-structured (Mo,V,Te,Nb)^47^ and MoV oxide^42^ catalyst systems. In general, however, systematic studies that simultaneously address chemically diverse catalyst materials and oxidation reactions of homologous compounds are uncommon.

High-throughput experiments have also been used to investigate a large number of catalysts, which were tested under the same conditions, for example, in propane oxidation over Mo-V-based catalysts.^67^ However, the downside of these experiments is that the synthesis is generally not optimized and a comprehensive and in-depth characterization of numerous catalysts synthesized in parallel is time- and resource-intensive.^42,49^ These shortcomings are addressed in this study by applying a holistic approach.

## Experimental Details

**Figure 2:**
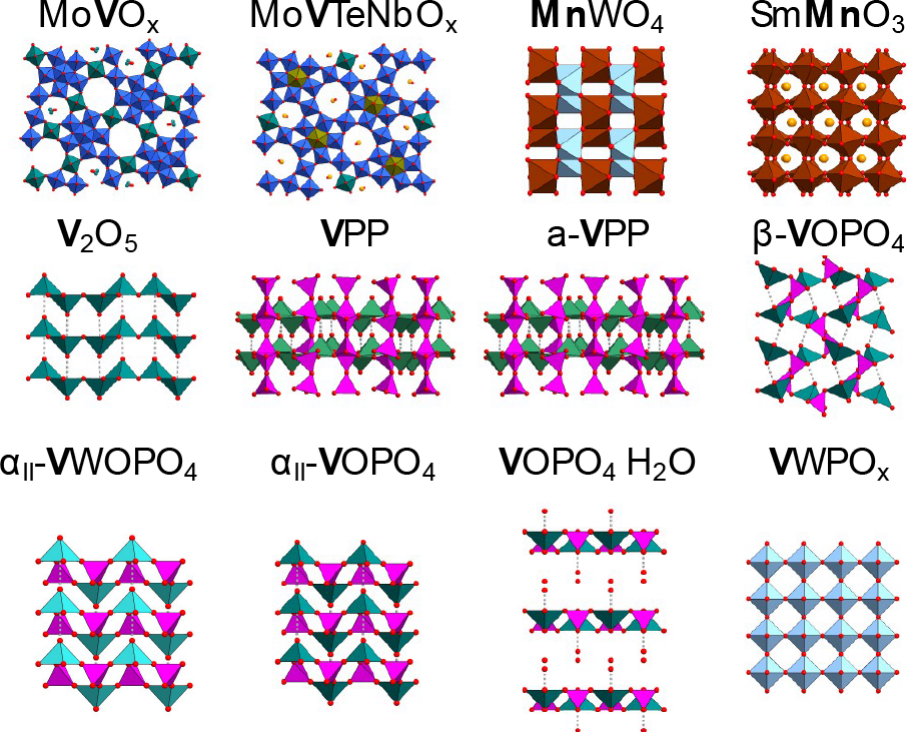
Schematic representation of the crystal structures of the 12 vanadium- and manganese-based catalysts analyzed in this work.

Here, we systematically investigate 12 catalysts (Figure 2) containing vanadium or manganese as redox-active elements (RAE) according to standard operating procedures described in a handbook. The handbook, which is also accessible via a link in the Supporting Information, establishes guidelines for kinetic analysis and the exact procedure for catalyst testing to ensure the exchange of catalyst data between the two laboratories involved in the experiments.

Ethane, propane, and *n*-butane oxidation reactions were performed to study the function of the catalysts. The choice of materials includes the industrial state-of-the-art catalyst for *n*-butane selective oxidation to maleic anhydride (MAN), vanadyl pyrophosphate (VPP or VPO) (VO)_2_P_2_O_7_, as well as the extensively researched MoVTeNbO_*x*_, a mixed-metal oxide “M1” phase catalyst. Following the structural diversity of bulk catalysts studied in oxidation catalysis, both amorphous and crystalline materials as well as single-phase compounds and phase mixtures were included in the sample set.

### Catalyst Synthesis

The synthesis of the catalyst precursors is described in the Supporting Information (the synthesis is also documented in a local database and the corresponding sample IDs are summarized in Table S2). A rather large batch size of 20 g was specified in order to provide enough material to carry out all three reactions as well as the comprehensive materials characterization on one and the same batch. The synthesis includes thermal treatments, pressing, and sieving. The so-obtained “fresh samples” are finally subjected to a rapid catalyst activation under harsh reaction conditions. The resulting samples are referred to as “activated samples”.

### Catalyst Test

**Figure 3:**
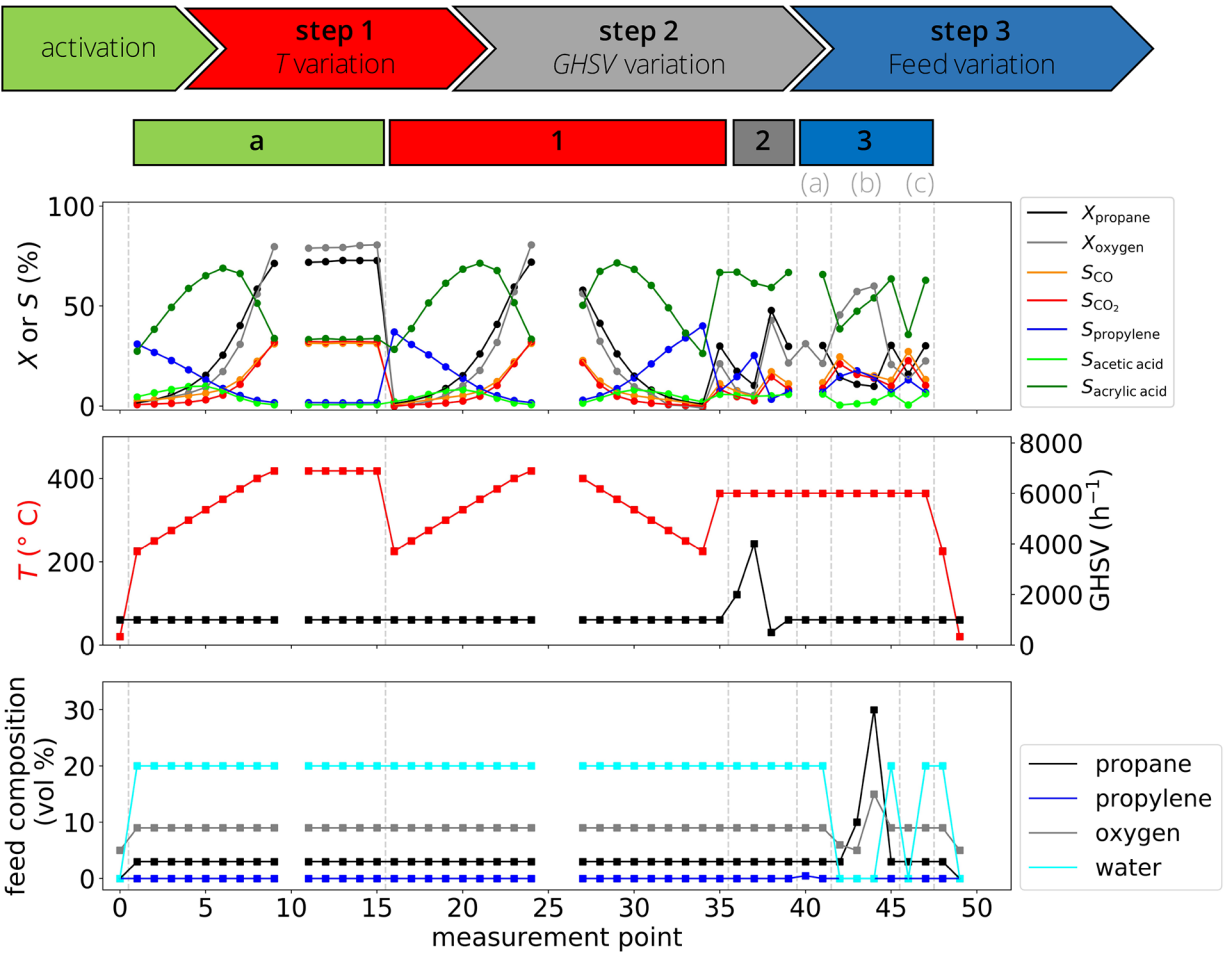
Handbook procedure for catalyst testing, illustrated for the example of MoVTeNbO_*x*_ applied to the propane oxidation reaction. The catalyst test starts with catalyst activation, during which the precursor is exposed to the rather harsh reaction environment for 48 h. Then, the test proceeds with three steps designed to acquire diverse kinetic information. At each measurement point, the steady-state gas-phase concentrations, and thus the alkane conversion and selectivity values, are measured (upper panel) at the chosen reaction conditions (middle and bottom panels). In the figure, *T* and *GHSV* stand for temperature and gas-hourly space-velocity, respectively. The figures corresponding to the remaining catalysts and reactions are available in the Supporting Information.

The functional (kinetic) analysis starts with a rapid activation procedure (Figure 3, in green), whose goal is to quickly bring the catalyst into a steady-state. Rapidly deactivating catalysts are also identified in this way. In this procedure, which takes 48 h, the fresh catalysts are exposed to rather harsh conditions. The rapid activation requirement is that the conversion of either alkane or oxygen reaches approximately 80% by increasing the temperature. The maximum temperature is limited to 450 °C in order to minimize the influence of gas-phase reactions.

Following the rapid activation, the catalyst test proceeds with three steps designed to generate basic kinetic information: temperature variation (step 1), contact time variation (step 2), and feed variation (step 3), shown in red, gray, and blue, respectively, in Figure 3. The feed variation step 3 is further split into three parts, in which (a) a reaction intermediate is co-dosed, (b) the alkane/oxygen ratios are varied at a fixed steam concentration, and (c) the water concentration is modified. Based on step 1, a reference temperature *T*_ref_ is determined for each catalyst and reaction. *T*_ref_ is the temperature, for which 30% alkane conversion is achieved in standard gas composition (see handbook in the Supporting Information). If 30% alkane conversion is not achieved, *T*_ref_ is considered the maximum temperature, 450 °C. Then, steps 2 and 3 are performed at *T*_ref_. Catalyst stability during the catalyst test is monitored by measurements under reference conditions (*T*_ref_ and standard gas feed composition) after each step. At each measurement point, a wide range of possible products are quantified (Table S3). For instance, 17 molecules are measured in the case of propane oxidation. Moreover, the oxygen conversion is determined. All measurements correspond to the steady state. At the end of the catalyst test, the “spent samples” are collected for characterization. The detailed description of the catalyst tests is presented in the Supporting Information.

### Catalyst Characterization

A total of 55 material properties and associated physicochemical parameters were measured (Table S4) using fresh, activated, and spent samples. Considering that the properties of activated and spent materials are unique for each reaction, 135 parameters per material were determined, that is, a total of 1620 quantities were measured. The fresh and activated samples were characterized using the ex situ techniques N_2_ adsorption, X-ray diffraction (XRD), X-ray fluorescence, and X-ray photoelectron spectroscopy (XPS). Temperature-programmed reduction and oxidation (TPRO) was conducted using the fresh catalysts. Advanced in situ near-ambient-pressure XPS (NAP-XPS) experiments at the synchrotron were carried out using the activated samples. Following the test protocol for kinetic analysis (Figure 3), three feed compositions were applied for each of the considered reactions in the NAP-XPS experiments: (i) dry, (ii) wet, and (iii) alkane-rich. We note that the slow catalyst formation processes under the in situ conditions used in NAP-XPS, which often differ from the real reaction conditions in a fixed-bed reactor,^45^ are avoided by the use of samples which were previously subjected to a rapid activation procedure. The detailed description of characterization experiments is provided in the Supporting Information, and more general guidelines to be followed in the characterization of catalysts are discussed in the handbook, which is accessible via a link in the Supporting Information. All raw data are also available by using this link.

## AI Approach

In order to identify correlations between physicochemical parameters and the catalyst performance, we apply the SISSO approach^36^ as implemented in the SISSO++ code.^68^ SISSO is tailored to the typical scenario in materials science and heterogeneous catalysis research, in which the number of characterized materials is small and the intricacy of underlying processes is high. SISSO identifies models as typically nonlinear analytical equations. From the model expressions, the key parameters emerge, enabling insights into the underlying processes. As such, SISSO determines interpretable descriptors.

The SISSO approach consists of three steps. (i) First, a set of input quantities, termed *primary features*, is chosen (Tables S4 and S5). These parameters characterize the materials in the data set and the potentially relevant processes that govern their performance. (ii) Second, mathematical operators (e.g., subtraction, multiplication, and logarithm) are applied to these primary features and then again and again to the resulting functions. As a result, an immense number of expressions—up to trillions—is generated, each of them describing a different possibly intricate interplay of underlying processes for each material of the data set. (iii) Then, SISSO identifies the few expressions, typically <4, which combined by weighting coefficients, best correlate with a chosen target performance of interest *F* (e.g., a material function) for the samples in the training set. The result is a model for the target of the form
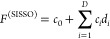
1where *d_i_* are the mentioned analytical expressions (features) composing a *D*-dimensional vector, referred to as the *descriptor*. The complexity of the model is determined by *D* and the number of times the mathematical operators are recursively applied to combine the primary features, denoted *q* and corresponding to the depth of the symbolic-regression tree, also referred to as the *rung*.

Because an immense number of possible expressions is considered in the analysis, SISSO can identify complex relationships based on much smaller data sets compared to those used for machine-learning approaches (e.g., neural networks or kernel-ridge regression), whose performance relies more strongly on the amount of data. However, it is then crucial that the data fed to SISSO are diverse and present high quality. We also note that SISSO captures nonlinear relationships that are overlooked in approaches such as the principal least square regression analysis.

In this work, we apply the multitask SISSO (MT-SISSO) transfer-learning approach^37^ to simultaneously model the reactivity toward the three oxidation reactions. Within MT-SISSO, one searches for a single descriptor but allows the fitted coefficients (*c*_0_ and *c_i_* in eq 1) to adapt to each of the tasks. Here, each reaction corresponds to one task. Thus, the fitted coefficients are different for each reaction, that is, *c_i_* = *c_i_*(*r*), where *r* corresponds to the reaction. We define the primary features corresponding to activated and spent samples, such as they assume different values depending on the modeled reaction, or, equivalently, depending on the task. The application of MT-SISSO with reaction-dependent primary features allows us to obtain *universal* descriptors for oxidation catalysis, that is, descriptors that are identical for all the considered reactions, even though the parameters entering in the descriptor expressions and the fitted coefficients assume different values for each reaction.

The optimal descriptor complexity with respect to its predictability is determined by using a leave-one-material-out cross-validation strategy (see details in Figures S1 and S2). This is done to prevent overfitting to the data set, as the expressions generated by SISSO are flexible and could fit the rather small number of materials used in this work nearly exactly but with the result of a lower prediction quality. The optimal complexity is considered the one which minimizes the averaged root-mean-squared error evaluated on left-out (predicted) materials (CV-RMSE). Only the models obtained at the identified optimal complexity using the whole data set for training, referred to as *best identified models*, are discussed.

## Results

### Catalyst Test

During activation, most of the catalysts immediately reach steady state, as shown by the results of the catalyst tests in Figures S3–S14. MoVTeNbO_*x*_ and MoVO_*x*_ (Figures S3 and S4, respectively) show slight deactivation in ethane oxidation. The phosphate catalysts turned out to exhibit very low activity in all feeds (Figures S8–S13) with the exception of the pyrophosphates (VPP and a-VPP, Figures S8 and S9, respectively). For these materials, the activity increases gradually during the activation step, in particular in *n*-butane oxidation. The catalysts predominantly show good stability after temperature and contact time variation, but feed variation and especially co-dosing experiments often lead to changes in the catalyst performance (Figures S3–S14).

**Figure 4:**
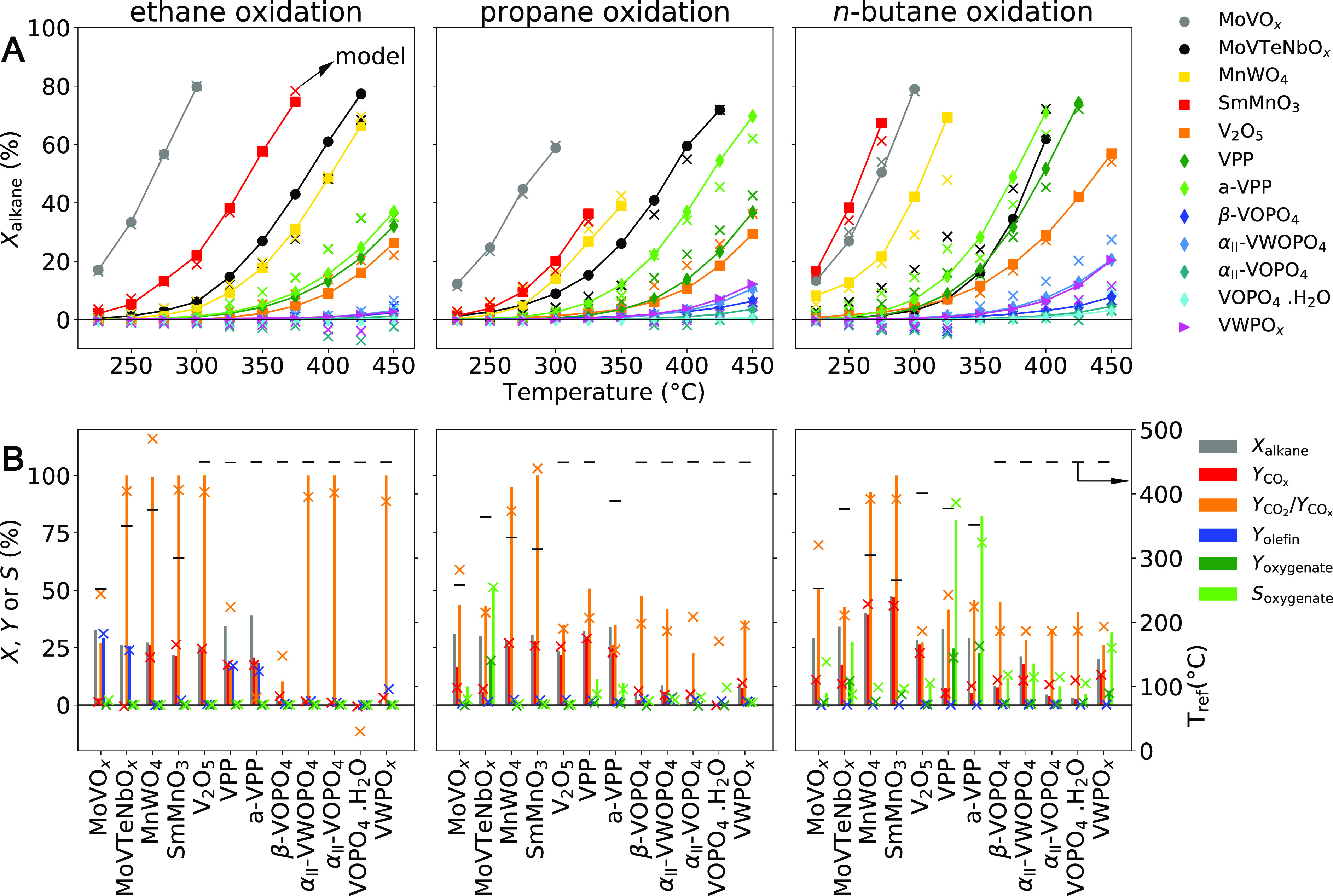
Overview of measured performance highlights the diverse catalytic behaviors among the 12 tested catalysts in terms of activity and selectivity. (A) Alkane conversion *X*_alkane_ during the temperature ramp applied in the catalyst test (Figure 3, step 1). (B): Conversion (*X*_alkane_) and product yield (*Y*_product_) at the reference temperature (*T*_ref_) as well as the oxygenate selectivity at low alkane conversion, *S*_oxygenate_. *T*_ref_ determined for each catalyst is displayed as a black bar and refers to the secondary axis (on the right). The selectivity was interpolated or extrapolated to 5% alkane conversion using the *S*_oxygenate_ vs *X*_alkane_ curves measured by increasing the temperature in the temperature variation step of the catalyst test. The bars are the experimental values (color code in the legend), and the crosses are the values corresponding to the SISSO models.

Three groups of catalysts can be roughly identified based on the alkane conversion *X*_alkane_ measured during the temperature variation step of the catalyst test (Figure 4A): (i) highly active catalysts, containing MoVO_*x*_, SmMnO_3_, and MnWO_4_; (ii) moderately active catalysts, containing MoVTeNbO_*x*_, V_2_O_5_, VPP, and a-VPP; and (iii) unactive catalysts, containing β-VOPO4, α_II_-VWOPO_4_, α_II_-VOPO_4_, VOPO_4_·2H_2_O, and VWPO_*x*_. However, some materials are more or less active depending on the reaction, making the classification nontrivial. For instance, MoVTeNbO_*x*_ is one of the most active catalysts in ethane or propane oxidation, but it has only moderate activity in *n*-butane oxidation. Additionally, while MoVO_*x*_ is the most active catalyst in ethane and propane oxidation, SmMnO_3_ is the most active material for *n*-butane oxidation. This shows that not only the C–H bond strength in the alkane molecule determines its reactivity, but also the catalyst intrinsic properties and the interaction between the alkane and the material play a role.

In addition to the alkane oxidation reactions, all catalysts were investigated in CO oxidation in oxygen-rich and oxygen-poor feeds. Only three catalysts are active for CO oxidation below 420 °C: SmMnO_3_, MnWO_4_, and α_II_-VWOPO_4_ (see light-off curves in Figure S15). The temperature at which 50% conversion is achieved increases in the following order: SmMnO_3_ < MnWO_4_ < α_II_-VWOPO_4_. Although the ranking of these three catalysts matches their ranking in terms of activity in all three alkane oxidations, there are other catalysts that are also very active in alkane oxidation but cannot oxidize CO. Therefore, CO oxidation cannot serve as a simple probe reaction for the alkane consumption rate.

In order to compare selectivity across different catalysts and reactions, we consider the product yields (denoted *Y*_product_) calculated as the selectivity to a specific product times the alkane conversion at *T*_ref_. The yield is considered in this analysis to take into account that different alkane conversions (shown by the gray bars in Figure 4B) are achieved by different catalysts.

The yield of combustion products CO and CO_2_, denoted *Y*_CO_*x*__, is above 20% among the catalysts with high and moderate activity (Figure 4B, red bars). Two specific total combustion catalysts can be identified: MnWO_4_ and SmMnO_3_. These materials provide relatively high *Y*_CO_*x*__, in particular in *n*-butane oxidation, and present a close-to-100% ratio between CO_2_ and CO_*x*_ yield (*Y*_CO_2__/*Y*_CO_*x*__, Figure 4B, yellow bars). This behavior correlates with the unique ability of these catalysts to oxidize CO (vide supra). Nevertheless, in general, CO oxidation cannot be used as a probe reaction for selectivity, since a number of catalysts which produce combustion products in alkane oxidation show no activity in CO oxidation. Although oxygen activation appears to definitely be an important factor influencing the yield of selective oxidation products, oxygen activation under CO oxidation conditions cannot serve as an indicator for alkane oxidation. This is because the surface of the catalyst might be different under CO oxidation conditions compared to the alkane oxidation conditions due to the different chemical potential of the reaction mixtures.

Olefin yields above 10% are observed in the case of ethane oxidation (Figure 4B, blue bars) for the catalysts MoVO_*x*_, MoVTeNbO_*x*_, VPP, and a-VPP. Regarding the oxygenate yield *Y*_oxygenate_, calculated considering only acetic acid, acrylic acid, or MAN, respectively, for C_2_, C_3_, and C_4_ oxidation reactions, it only reaches values above 10% for propane and *n*-butane oxidation (Figure 4B, dark green bars). In propane oxidation, the catalyst producing the highest *Y*_oxygenate_ is MoVTeNbO_*x*_. In *n*-butane oxidation, three materials provide *Y*_oxygenate_ above 10%: MoVTeNbO_*x*_, VPP, and a-VPP. Finally, we also evaluated the selectivity toward oxygenates at the fixed alkane conversion value of 5%, denoted *S*_oxygenate_ (Figure 4B, light green bars). The materials for which *S*_oxygenate_ is high are the same as those for which *Y*_oxygenate_ is relatively high, with the exception of VWPO_*x*_. VWPO_*x*_ presents relatively high *S*_oxygenate_ values in *n*-butane oxidation, in spite of the rather low *Y*_oxygenate_.

### Catalyst Characterization

After synthesis, the materials were generally present as polycrystalline solids, with the exception of the “amorphous” vanadium-phosphorus oxide, referred to here as a-VPP, which contains an amorphous component and poorly crystalline hemihydrate VOHPO_4_·1/2H_2_O. As far as possible, the observed diffraction patterns were analyzed and interpreted using whole powder pattern fitting according to the Rietveld method. The corresponding crystal structure and peak shape models used are listed in Table S6. A detailed analysis of the XRD data and a discussion of possible structural changes observed after catalysis are provided in the Supporting Information (see text and Tables S6–S11 and Figure S16).

The specific surface area of the fresh catalysts varies between 0.70 and 60 m^2^/g. The mostly macroporous materials have total pore volumes below 0.5 cm^3^/g. Structural transformations and crystallization processes that take place during the activation procedure are accompanied by an increase or decrease in the specific surface area and the pore volume (Figure S17), and changes in the surface composition are thus to be expected. The changes differ depending on the reaction carried out, especially with regard to the pore volume (Figure S17A). Even the phase-pure materials, for which no phase transformations are observed under reaction conditions, exhibit considerable fluctuations in their interfacial properties.

**Figure 5:**
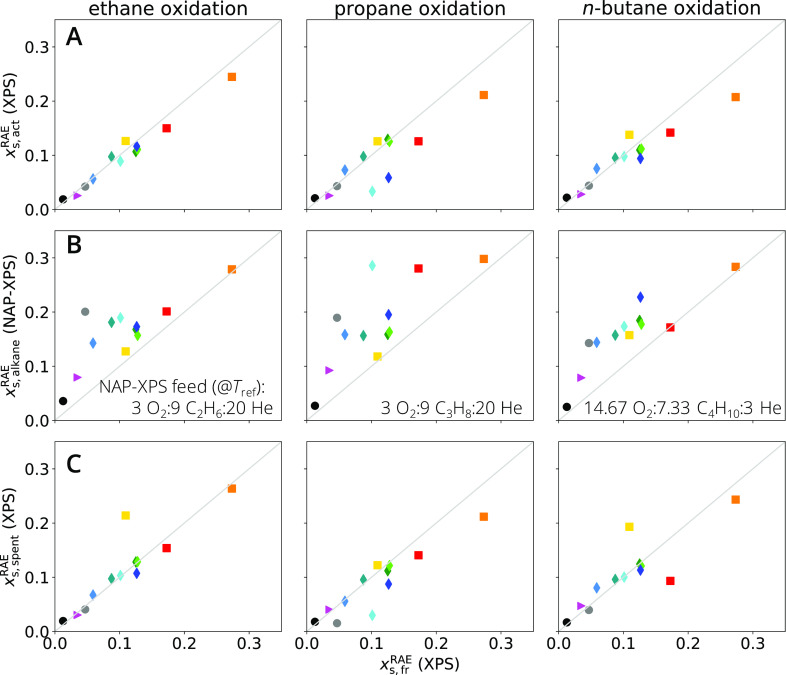
Quantifying the environment-dependent catalyst restructuring: the surface redox-active-element (RAE) content of (A) activated materials, (B) materials under reaction conditions, and (C) spent materials (shown in the ordinates) is different from that of the fresh catalyst (shown in the abscissa), indicating that the material surface adapts to the environment to which it is—or was—exposed. The surface stoichiometry of the fresh, activated, and spent materials are measured by ex situ XPS under UHV, while that of the material under reaction condition is measured by in situ NAP-XPS at alkane-rich feed (feed compositions shown in B). The surface content as well as the surface electronic properties measured under dry and wet feeds by NAP-XPS are shown in Figures S18 and S19. The catalysts are represented using the same convention as for Figure 4.

The connection between the surface composition and the chemical potential of the environment becomes clear through a detailed analysis of the XPS data. The surface contents of the RAEs vanadium or manganese on fresh catalyst samples, denoted *x*_s, fr_^RAE^ and measured by XPS at ultra-high-vacuum (UHV) conditions, are displayed in the abscissa of the plots in Figure 5. The values lie in the range 0.01–0.30, in units of atomic fraction. The perovskite SmMnO_3_ and the vanadium oxide V_2_O_5_ present the highest RAE surface contents, whereas the mixed-metal oxides MoVO_*x*_, MoVTeNbO_*x*_, and VWPO_*x*_ are the materials with lowest values. The comparison of RAE surface content with activity toward the analyzed oxidation reactions does not suggest a direct link.

The surface RAE content of fresh samples (abscissa) is compared with that of the activated samples under UHV, denoted *x*_s, act_^RAE^ (ordinate), in Figure 5A, for each reaction. Most of the materials present similar RAE surface contents on fresh vs activated catalyst samples, corresponding to data points close to the diagonal. However, some catalysts have a significantly lower *x*_s, act_^RAE^ compared to *x*_s, fr_^RAE^, for instance, SmMnO_3_, V_2_O_5_, β-VOPO_4_, and VOPO_4_·2H_2_O after activation in wet propane feed at high conversion. The decrease in surface RAE content upon rapid activation can be ascribed to phase changes, the loss of volatile compounds, and/or to the migration of vanadium or manganese to the material’s bulk.

The comparison of RAE surface content of the fresh samples with the surface RAE content of the activated samples under NAP alkane-rich conditions, denoted *x*_s, alkane_^RAE^ in Figure 5B, shows that the surface RAE content increases for most materials and reactions. The difference between *x*_s, act_^RAE^, measured by XPS, and *x*_s, alkane_^RAE^, measured by NAP-XPS, can be attributed on the one hand to the different depth of information. NAP-XPS examines the outer surface (inelastic mean free path λ_NAP_^RAE^ = 0.53 – 0.74 nm), while XPS includes the composition of the subsurface (λ_lab_^RAE^ = 1.19 – 2.06 nm). On the other hand, the chemical potential in the gas phase has a significant influence on the surface composition, as can be seen from the comparison of the surface concentrations of almost all catalysts containing at least two metals in the dry, wet, and alkane-rich feed determined by NAP-XPS (Figure S18). The increase in RAE during the in situ measurement (Figure 5B) is significantly more pronounced than the changes observed between *x*_s, fr_^RAE^ and *x*_s, act_^RAE^ in Figure 5A. This is in particular the case for the phosphate-based materials and MoVO_*x*_ (displayed as diamonds and gray circles, respectively, in Figure 5).

The analysis of spent catalysts (Figure 5C) shows similar RAE surface contents compared to the activated samples (Figure 5A), indicating that the changes in the surface occurring during the reaction are reversible. The changes in RAE surface contents under reaction conditions are accompanied by changes in RAE oxidation states, as shown in Figure S19 and Tables S12 and S13.

These results highlight that the materials’ structure and properties, in particular on the surface, adapt to the environment. This phenomenon is captured by the application of in situ spectroscopy. Additionally, the comparison between fresh and activated samples (Figure 5A) shows that the materials properties depend on the environment to which it *was* exposed. Finally, we note that this systematic interface analysis according to the handbook procedures not only enables a *qualitative* discussion of the underlying restructuring, but it also provides a *quantification* of the environmental effect on catalyst’s properties.

### Property–Function Relationships

**Figure 6:**
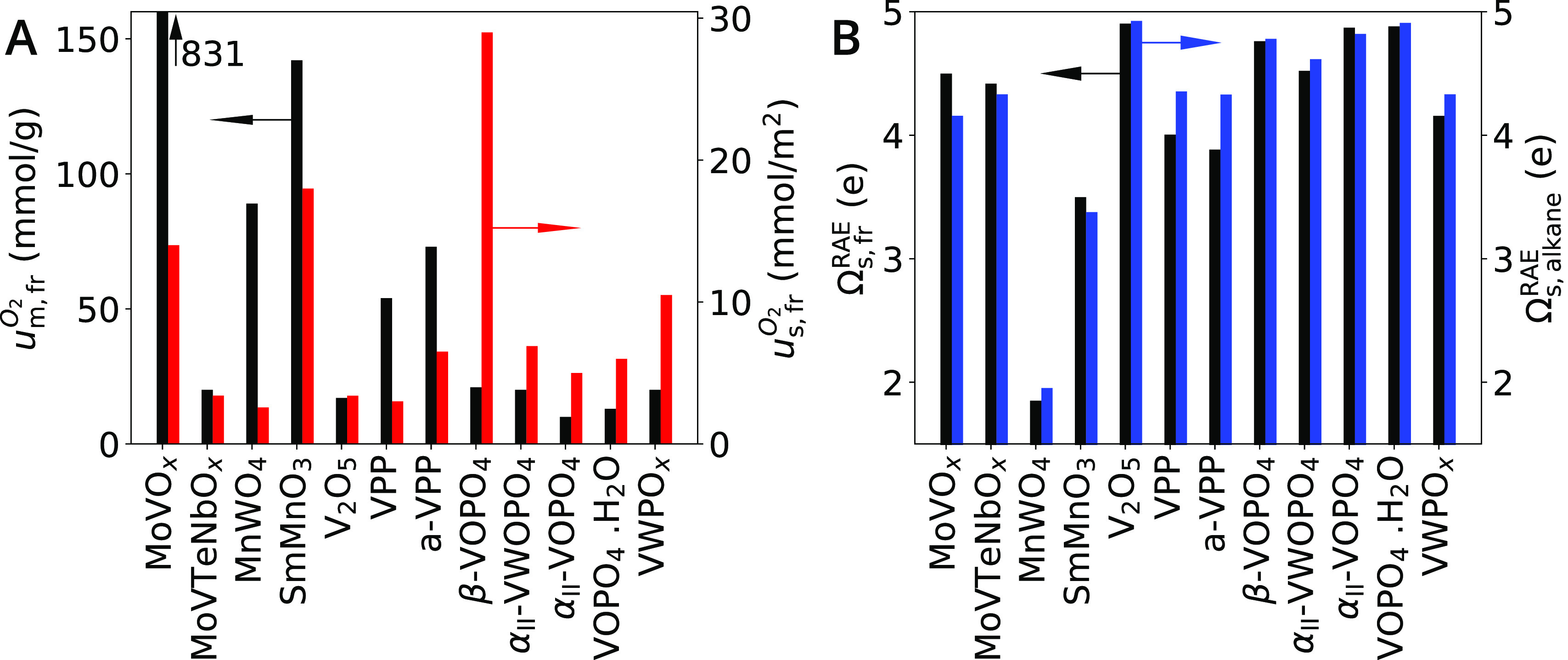
Quantifying the bulk and surface redox properties: (A) Reversible oxygen uptake of the fresh catalyst samples after TPRO up to the maximum reaction temperature in the catalytic test per mass (*u*_m, fr_^O_2_^) and per surface area (*u*_s, fr_^O_2_^). (B) Surface RAE oxidation states measured on the fresh catalyst samples by XPS (Ω_s, fr_^RAE^) and surface RAE oxidation states measured on the activated catalyst samples under alkane-rich feed by NAP-XPS (Ω_s, alkane_^RAE^).

Based on the predominantly empirical knowledge in oxidation catalysis, mainly structural properties of the bulk and the associated redox properties of the materials have been proposed as descriptors of reactivity in alkane oxidation. However, we find that such parameters alone are not able to consistently capture the reactivity trends across the materials and reactions analyzed in this work. For instance, it is expected that the more activated oxygen the catalyst can offer, the higher its activity. Such redox capacity is captured by the reversible oxygen uptake measured by TPRO. The reversible oxygen uptake per mass and per surface area measured on the fresh catalyst samples (denoted *u*_m, fr_^O_2_^ and *u*_s, fr_^O_2_^, respectively) are shown in black and red in Figure 6A, respectively, for the 12 investigated catalysts. The reversible oxygen uptake per mass correlates with the high activity of MoVO_*x*_, SmMnO_3_, and MnWO_4_ (Figure 4A, in gray, red and yellow, respectively). However, this parameter does not capture that MoVTeNbO_*x*_ or V_2_O_5_ have moderate activity, since the oxygen uptakes for these materials are comparable to the values corresponding to the low-activity catalysts β-VOPO_4_, α_II_-VWOPO_4_, α_II_-VOPO_4_, VOPO_4_·2H_2_O, and VWPO_*x*_. The reversible oxygen uptake per surface area is not able to fully account for the observed activity trend either.

The use of intrinsic materials properties as descriptors for selective oxidation is problematic because they are reaction-independent, that is, they neglect any effect resulting from the interaction of the reaction mixture with the material. The chemical potential under reaction is, however, known to influence the reactivity in alkane oxidation.^42,45,69−71^ For this reason, we have also verified whether the surface RAE oxidation state measured under UHV on the fresh catalyst samples and under NAP alkane-rich conditions (denoted Ω_s, fr_^RAE^, and Ω_s, alkane_^RAE^, respectively, in Figure 6B, Table S13) correlates with the reactivity. The VPP and a-VPP materials, which are highly selective toward MAN in *n*-butane oxidation (Figure 4B), present the highest increase in oxidation state under NAP conditions with respect to UHV (compare black and blue bars in Figure 6B). In spite of these observations, no general correlation between the surface RAE oxidation states and activity or selectivity in alkane oxidation is apparent.

In summary, the direct comparison of the experimental results presented above demonstrates that the handbook method enables a quick and fair ranking of different catalysts and that reference systems can be established in this way. It also becomes clear that the classification of catalysts must always be seen in the context of a specific reaction, since the reactivity depends both on the material and on the feed composition. Particularly because the choice of materials in our data set is based on previous work in oxidation catalysis (Table S1), a wide diversity of behaviors in selective oxidation is represented under the considered reaction conditions. Catalysts and reaction conditions providing value-added olefins and oxygenates as well as the undesired combustion products or inactive catalysts are all present in the data. However, for reactions as complicated as the oxidation of alkanes, the empirical structure– or property–reactivity relationships are not apparent from simple correlations.

### SISSO Analysis

In order to identify potentially nonlinear correlations between several measured physicochemical parameters and the catalyst performance, we applied the SISSO approach. As primary features, we offered the 55 materials properties and associated parameters shown in Table S4. In particular, parameters describing the interaction of the catalyst materials with the reaction environment are included by means of the properties of activated/spent materials as well as by the use of information obtained from in situ NAP-XPS measurements. We note that the diversity of physical properties and catalytic behaviors present in our data set is crucial for the derivation of property–function relationships by AI, since the algorithm needs to be exposed to diverse behaviors (e.g., selective and unselective catalysts) in order to “learn” the reactivity patterns. Indeed, including information on materials and reaction conditions related to undesired (mediocre) performance is helpful.^72^

We start discussing the SISSO analysis of catalyst activity. For this purpose, we chose the alkane conversion during the (increasing) temperature ramp step of the catalyst test (step 1 in Figure 3), denoted *X*_alkane_, as target. For this target, we model all measured temperatures within the range 225–450 °C simultaneously by letting the model fitted coefficients adapt not only to the reaction, but also to the temperature, that is, *c_i_* = *c_i_*(*r*, *T*), where *T* corresponds to the measured temperature. The total number of data points is 311, corresponding to 12 catalysts × 3 reactions × ca. 10 measured temperatures.

**Figure 7:**
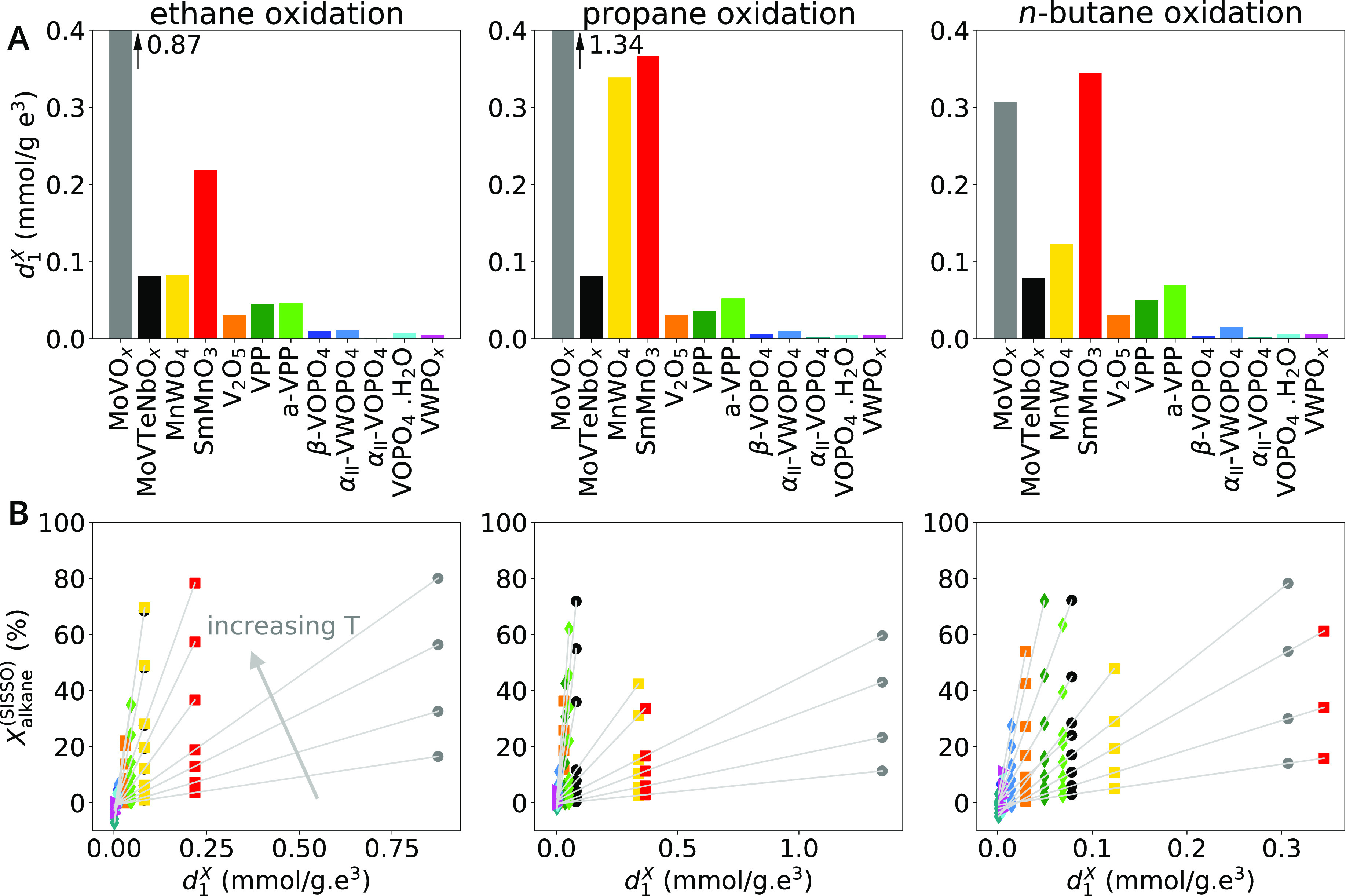
Descriptor for alkane conversion identified with SISSO based on 311 data points captures the activity of the 12 oxidation catalysts across the three considered reactions. (A) Descriptor *d*_1_^*X*^ values. (B) Alkane conversion estimated by the SISSO models. The coefficients of the model *c*_0_^*X*^ and *c*_1_^*X*^ adapt to each reaction and temperature, while the descriptor *d*_1_^*X*^ assumes one single expression in all cases (see Table 1). The *d*_1_^*X*^ values for a fixed material are different for each reaction because the parameters entering the expression are reaction-dependent. The catalysts are represented in B using the same convention as for Figure 4.

The comparison between the measured conversion and the fit of the best identified model for alkane conversion, denoted *X*_alkane_^(SISSO)^, shown as solid markers and crosses in Figure 4A, respectively, indicates the good quality of the fit. The values for the 1-dimensional (1-D) descriptor component (*d*_1_^*X*^) evaluated for each material and reaction, shown in Figure 7A, further highlights that the descriptor correctly captures the performance across materials. In particular, the descriptor distinguishes the most active catalysts (MoVO_*x*_, MoVTeNbO_*x*_, SmMnO_3_, and MnWO_4_) from the remaining ones. Moreover, the reactivity trend across reactions is also well described. The fact that SmMnO_3_ is more active than MoVO_*x*_ (in red and gray, in Figure 7A) only for the case of *n*-butane oxidation, for instance, is captured. Because the *X*_alkane_^(SISSO)^ model is based on the same descriptor *d*_1_^*X*^ for all temperatures and reactions, it can be written as *X*_alkane_^(SISSO)^ = *c*_0_^*X*^(*r*, *T*) + *c*_1_^*X*^(*r*, *T*)*d*_1_^*X*^ and graphically represented as multiple straight lines in a *X*_alkane_^(SISSO)^ vs *d*_1_^*X*^ plot (Figure 7B), each of them corresponding to a different temperature.

**Table 1:**
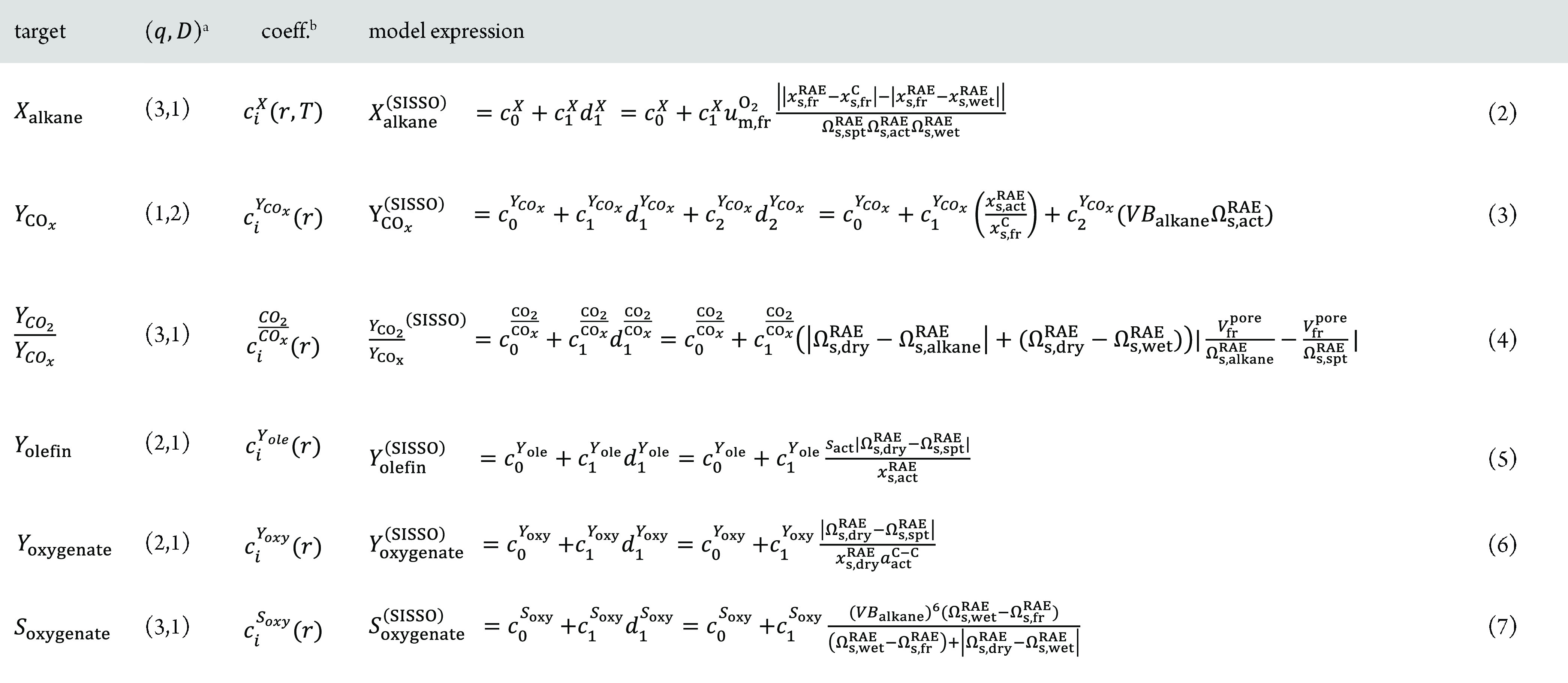
Models Identified for Catalyst Performance in Ethane, Propane, and *n*-Butane Oxidation Reactions by the SISSO Approach Using the 12 Vanadium- and Manganese-Based Catalysts and the 55 Different Physicochemical Parameters Measured for Each Catalyst (and Reaction)

a Optimal model complexity with respect to predictability identified by leave-one-material-out cross-validation. *q* and *D* correspond to the depth of the symbolic-regression tree and to the descriptor dimension, respectively.

b With the application of MT-SISSO, the fitted model coefficients (coeff.) are allowed to adapt to each reaction, that is, *c_i_* = *c_i_*(*r*), where *r* stands for reaction (ethane, propane, or *n*-butane oxidation), or simultaneously to each reaction and temperature, that is, *c_i_* = *c_i_*(*r*, *T*), in the case of the model *X*_alkane_^(SISSO)^.

**Table 2:** Key Parameters or “Materials Genes” Identified for Catalyst Performance in Ethane, Propane, and *n*-Butane Oxidation Reactions by the SISSO Approach Using the 12 Vanadium- and Manganese-Based Catalysts

parameter	technique	unit	description	related underlying process
*u*_m, fr_^O_2_^	TPRO	μmol O_2_·g^–1^	O_2_ uptake per mass	bulk and surface
				redox capacity
				
*x*_s, fr_^RAE^	XPS	fraction atom	surface atomic content of the redox active element (RAE)	
*x*_s, act_^RAE^	XPS			surface redox activity
*x*_s, dry_^RAE^	NAP-XPS^a^			and restructuring
*x*_s, wet_^RAE^	XPS			
				
*x*_s, fr_^C^	XPS	fraction atom	surface atomic content of carbon	adsorption
				
*a*_act_^C – C^	XPS	fraction area	amount of C 1s, C–C component	surface sensitivity
				
Ω_s, fr_^RAE^	XPS	e^c^	surface oxidation state of the RAE	
Ω_s, act_^RAE^	XPS			
Ω_s, spt_^RAE^	XPS			surface redox activity
Ω_s, wet_^RAE^	NAP-XPS^b^			and restructuring
Ω_s, dry_^RAE^	NAP-XPS^b^			
Ω_s, alkane_^RAE^	NAP-XPS^b^			
				
*VB*_alkane_	NAP-XPS^b^	eV	valence-band onset	surface charge transfer
				
*V*_fr_^pore^	N_2_ ads.	cm^3^·g^–1^	pore volume per mass	local transport
*s*_act_	N_2_ ads.	m^2^·g^–1^	surface area per mass	

a The subscripts “fr”, “act”, and “spt” indicate properties measured on fresh, activated, and spent catalyst samples, respectively.

b In situ measurement under reaction conditions. The subscripts “dry”, “wet”, and “alkane” correspond to the three different reaction feeds applied at *T*_ref_: dry, wet, and alkane-rich feed, respectively.

c Elementary electron charge.

The expression for the alkane-conversion descriptor *d*_1_^*X*^, displayed in eq 2 of Table 1, reveals the key primary features correlated with the alkane conversion (highlighted in Table 2): the per-mass reversible oxygen uptake of the fresh catalyst (*u*_m, fr_^O_2_^), the carbon surface contents of the fresh catalyst under UHV (*x*_s, fr_^C^), the RAE surface content of the fresh catalyst under UHV and of the activated catalyst under wet feed (*x*_s, fr_^RAE^and *x*_s, wet_^RAE^, respectively), the surface RAE oxidation states of the activated and spent catalysts under UHV (Ω_s, act_^RAE^, Ω_s, spt_^RAE^), and surface RAE oxidation states of the activated catalyst under wet feed (Ω_s, wet_^RAE^). The relevance of these parameters reflects that the activity in alkane oxidation is governed by an interplay between the catalyst bulk and surface redox capacity (encoded by *u*_m, fr_^O_2_^, Ω_s, act_^RAE^, Ω_s, spt_^RAE^, and Ω_s, wet_^RAE^) with surface processes depending on the surface composition, in particular the adsorption strength (encoded by *x*_s, fr_^C^) and the availability of surface vanadium or manganese (encoded by *x*_s, fr_^RAE^, and *x*_s, wet_^RAE^). The absolute difference between the RAE content on the fresh catalyst and the RAE content on the activated catalyst under wet reaction conditions |*x*_s, fr_^RAE^ – *x*_s, wet_^RAE^| is identified by SISSO as one of the ingredients of the model. This points at the role of catalyst restructuring at the reaction conditions. The relevance of reversible oxygen uptake for catalyst activity is in line with previously proposed descriptors of oxidation catalysis.^73^ However, the more complex descriptor expression identified indicates that additional surface processes and the restructuring of the material play in concert with the materials’ redox capacity to determine alkane conversion. Interestingly, the specific surface area, which is intuitively associated with the activity of the catalysts, is not selected among the key primary features.

In order to assess the importance of in situ characterization data for describing the activity in alkane oxidation, we have also performed the SISSO analysis by excluding the primary features obtained from NAP-XPS (Table S14 and Figure S20). The minimum CV-RMSE significantly increases from 6.19 to 10.87% when the NAP-XPS information is not included. Moreover, the optimal complexity of the descriptor obtained without the in situ primary features is lower (*q* = 1) compared to that of eq 2 (*q* = 3), which is reflected in the worse fit of the best model to the data set (Figure S21). In particular, the model obtained without the NAP-XPS data is unable to capture that the SmMnO_3_ becomes more active than MoVO_*x*_ in the case of *n*-butane oxidation. The best model identified without the in situ primary features is based on a 2-D descriptor with components  and *u*_m, fr_^O_2_^*x*_s, act_^RAE^. These results indicate that the in situ information is crucial for modeling the reaction-dependent *X*_alkane_.

We next address the catalyst selectivity in alkane oxidation reactions based on the targets *Y*_CO_*x*__, *Y*_CO_2__/*Y*_CO_*x*__, *Y*_olefin_, and *Y*_oxygenate_. The total number of data points is 36 for each target, corresponding to 12 catalysts × 3 reactions. The fits of the best identified models to the measured targets, shown as crosses and bars in the lower panels of Figure 4B, respectively, show that the models are able to describe the experimental trends across materials and reactions.

**Figure 8:**
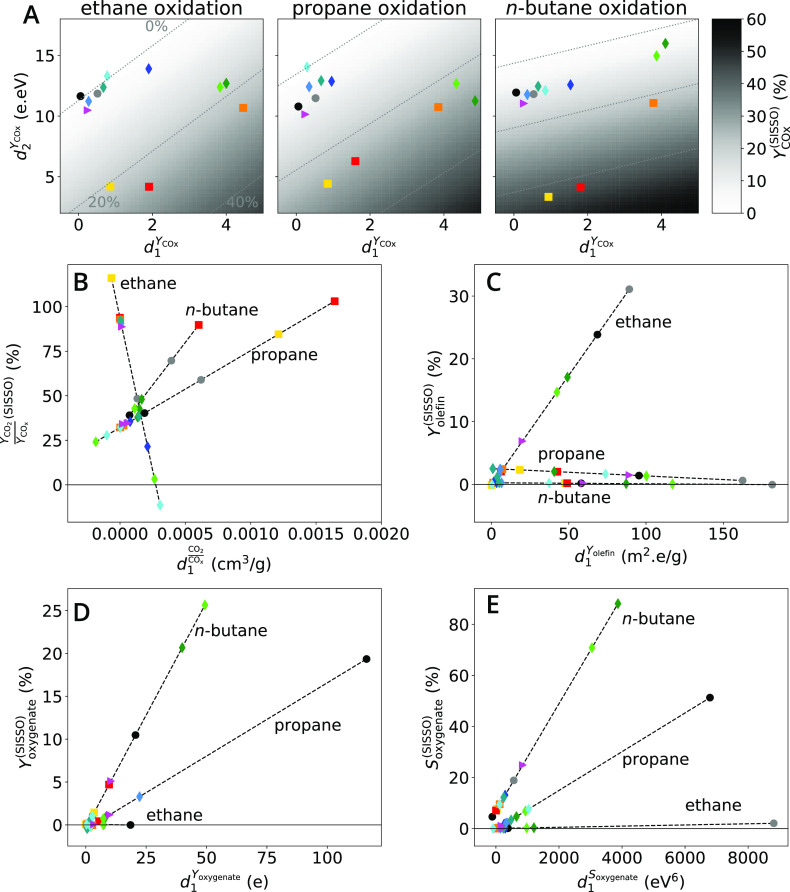
Descriptors for product yield and selectivity identified with SISSO based on 36 data points describe the selectivity of the 12 oxidation catalysts across the three considered reactions. The coefficients of the model adapt to each reaction, while the descriptor assumes one single expression per target (see Table 1). The descriptor values for a fixed material are different for each reaction because the parameters entering the expression are reaction-dependent. The figures correspond to the targets *Y*_CO_*x*__(A), *Y*_CO_2__/*Y*_CO_*x*__(B), *Y*_olefin_ (C), *Y*_oxygenate_ (D), and *S*_oxygenate_ (E). The identified descriptor for *Y*_CO_*x*__ is 2-D, while the identified descriptors for the remaining targets are 1-D. The catalysts are represented using the same convention as for Figure 4.

The best identified model for the yield of CO and CO_2_ is 2-D, and it is shown in eq 3 of Table 1. The *Y*_CO_*x*__^(SISSO)^ expression contains the carbon and RAE surface contents on the fresh and activated catalysts under UHV (*x*_s, fr_^C^ and *x*_s, act_^RAE^, respectively), the surface RAE oxidation state of the activated catalyst under UHV (Ω_s, act_^RAE^), and the valence-band onset under alkane-rich feed (*VB*_alkane_). These key parameters reflect that the formation of the combustion products is governed by surface processes correlated to the surface composition, for instance, the adsorption strength and availability of surface redox-active species, encoded by *x*_s, fr_^C^,and *x*_s, act_^RAE^, respectively, as well as the charge transfer from the surface to adsorbed species, encoded by Ω_s, act_^RAE^, and *VB*_alkane_. The 12 catalysts are represented in the coordinates of the 2-D descriptor in Figure 8A. They are roughly located in three portions of the plot, containing (i) the combustion catalysts MnWO_4_ and SmMnO_3_, (ii) VPP, a-VPP, and V_2_O_5_, which produce significant CO_*x*_ in ethane and propane oxidation, and (iii) the remaining catalysts, which provide low *Y*_CO_*x*__. In this “map of potential catalysts”, the values predicted by the model (*Y*_CO_*x*__^(SISSO)^) are indicated by the gray scale. Interestingly, the gradient of *Y*_CO_*x*__^(SISSO)^, determined by the reaction-dependent fitted coefficients, shows that , which contains the electronic properties Ω_s, act_^RAE^ and *VB*_alkane_, becomes more important than , as one moves from ethane, to propane and *n*-butane oxidation. This can be related to the number of electrons that needs to be transferred from the catalyst surface to the adsorbed species in order to achieve combustion, which increases in the order C_2_ < C_3_ < C_4_ oxidation.

We have also considered the ratio *Y*_CO_2__/*Y*_CO_*x*__ as target in our analysis to obtain insights on the mechanisms driving the formation of the total vs partial combustion product. In the identified expression of the 1-D  model (eq 4, Table 1) the surface RAE oxidation states of the spent catalyst sample under UHV (Ω_s, spt_^RAE^), the surface RAE oxidation states under dry, alkane-rich and wet feeds (Ω_s, dry_^RAE^, Ω_s, alkane_^RAE^, and Ω_s, wet_^RAE^, respectively) and the pore volume of the fresh catalyst (*V*_fr_^pore^) appear. These parameters correlate with the surface redox activity and local transport. In the relatively complex expression of the model, the differences between oxidation states measured in situ at different chemical potentials, i.e., (Ω_s, dry_^RAE^ – Ω_s, alkane_^RAE^) and (Ω_s, dry_^RAE^ – Ω_s, wet_^RAE^), are identified as important ingredients. This suggests that total oxidation to CO_2_ is favored on catalysts for which the RAE can change its oxidation state over a wide range for different environments.

In the expression of best identified model describing the olefin yield *Y*_olefin_^(SISSO)^ (eq 5, Table 1), the specific surface area of the activated catalyst (*s*_act_), the surface RAE content of the activated catalyst under UHV (*x*_s, act_^RAE^), the surface RAE oxidation states on the spent catalyst under UHV (Ω_s, spt_^RAE^), and the surface RAE oxidation states under dry feed (Ω_s, dry_^RAE^) appear. These parameters can be associated to the relevance of local transport (the density of active sites is approximated by *x*_s, act_^RAE^/*s*_act_), availability of surface redox-active species, and surface redox activity. The equation implies that olefin formation appears to be a local surface process for which the electronic properties of the catalyst volume do not play a major role. Site isolation of the active sites also has a positive effect on the selectivity to the olefin, since the lower the *x*_s, act_^RAE^ values in eq 5, the higher is the olefin yield. For ethane oxidation, the fitted *c*_1_^*Y*_olefin_^ is positive, while it assumes a negative value for propane oxidation (Figure 8C). The change in the sign of the coefficient can be related to the stability of the olefin formed. In particular, the consecutive reaction of propylene to form an allyl surface intermediate can lead to additional reaction pathways that lower the olefin selectivity, which is not possible in the case of the oxidative dehydrogenation of ethane to ethylene.

The best identified model for oxygenate yield *Y*_oxygenate_^(SISSO)^, shown in eq 6 (Table 1), depends on the surface RAE content under dry feed (*x*_s, dry_^RAE^), the surface RAE oxidation state of the spent catalyst under UHV (Ω_s, spt_^RAE^), the surface RAE oxidation state under dry feed (Ω_s, dry_^RAE^), and on the relative amount of carbon assigned to C–C in the activated sample under UHV (*a*_act_^C – C^). These parameters correlate with the availability of surface redox-active species, the surface redox activity, and the importance of specific surface sites where reactants, reaction intermediates, or products might adsorb. The *c*_1_^oxy^ values increase from ethane to propane and *n*-butane oxidation (Figure 8D).

The difference Ω_s, spt_^RAE^ – Ω_s, wet_^RAE^ appears as an important ingredient in the model expressions for both *Y*_olefin_^(SISSO)^ and *Y*_oxygenate_^(SISSO)^ models. Indeed, both targets are proportional to the absolute difference |Ω_s, spt_^RAE^ – Ω_s, wet_^RAE^| in eqs 5 and 6. Thus, the production of value-added olefins and oxygenates correlates with the change in surface RAE oxidation state that occurs from UHV to reaction conditions. This can be associated to the response of the catalyst to the environment and to the formation, on selective catalysts, of a surface termination layer that has different properties compared to the bulk phase.

Descriptors for product yield are relatively difficult to interpret, as the target simultaneously depends on activity and selectivity. For this reason, we have also derived a model for the oxygenate selectivity at a fixed low alkane conversion degree (5%), denoted *S*_oxygenate_^(SISSO)^ and shown in eq 7 of Table 1. It contains, as key parameters, the valence-band onset under alkane-rich feed (*VB*_alkane_), the surface RAE oxidation state of the fresh catalyst under UHV (Ω_s, fr_^RAE^), and the surface RAE oxidation state under dry and wet feeds ( Ω_s, dry_^RAE^ and Ω_s, wet_^RAE^, respectively). *VB*_alkane_ can be related to the surface charge transfer from the catalyst to adsorbed species. Ω_s, fr_^RAE^, Ω_s, dry_^RAE^, and Ω_s, wet_^RAE^ point to the buffer function of a selective oxidation catalyst, which ensures that the change in the oxidation state of the RAE remains within limits and thus overoxidation to CO or CO_2_ is avoided.^69,70^

The models derived by SISSO are expected to be valid as long as the reactivity is governed by the processes that determine the catalytic performance for the materials in the data set. However, for describing regions of the materials space containing catalysts significantly different from those in the data set, which might function according to different underlying processes, the models might need to be re-trained with more data. We also note that, in addition to the underlying processes indicated in Table 2, it is possible that other, so far not well understood processes are captured by AI, as the relationship between identified parameters and the underlying chemistry might be indirect, that is, the identified correlations do not necessarily reflect direct causality.

## Discussion

By using standardized protocols fully described in an experimental handbook, 12 oxidation catalysts were synthesized, extensively characterized and tested in ethane, propane, and *n*-butane oxidation. The application of the rigorous protocols, which take into account the kinetics of formation of the catalyst active states, enabled a consistent comparison of materials properties and reactivity toward the three reactions. By applying AI, we demonstrated that the reactivity is correlated with multiple materials properties in a nonlinear way. This highlights that several underlying processes play in concert to determine the performance in selective oxidation. Therefore, AI is not an addition to the heterogeneous catalysis research toolkit, but rather a prerequisite for the identification of property–function relationships that are overlooked by simpler (e.g., linear) models.

We simultaneously modeled ethane, propane, and *n*-butane oxidation reactions using MT-SISSO with reaction-dependent primary features. This allowed us to obtain *universal* property–function relationships in the form of analytical expressions. These relationships apply to all three oxidation reactions at the same time, even though they incorporate the response of the catalyst to each specific reaction environment. Indeed, the key physicochemical parameters identified by AI are not only catalyst properties under thermodynamic standard conditions, but also properties of the *working catalysts*. Therefore, the crystal structure and translational repetitive arrangement of atoms in the surface, used in conventional catalyst design approaches and theoretical models, is insufficient to describe selective oxidation catalysis. The crucial role of the catalyst modulation by the reaction environment highlighted by AI is consistent with the fact that the application of simple test reactions, such as TPRO or CO oxidation, does not provide an efficient identification of potential catalysts. The catalyst is in a different chemical state under the conditions of the probe reaction compared to the case of alkane oxidation reaction conditions due to the different chemical potential. Thus, the incorporation of in situ characterization into catalyst design strategies is crucial.

The key physicochemical parameters identified by AI relate to the underlying processes governing selective oxidation. Besides, the identified models show how these parameters may be tuned in order to achieve improved performance. For the case of the target olefin yield, for instance, local transport, site isolation, surface redox activity, and surface restructuring are the important underlying processes indicated by the parameters specific surface area, surface RAE content, and RAE oxidation state. To achieve high olefin yields, the catalyst must have a high specific surface area, a low concentration of surface RAE, and the ability to change the surface RAE oxidation state under reaction conditions with respect to vacuum. In the case of the desired formation of valuable oxygenates, the catalyst must also display specific surface sites on which reactants, intermediates, or products might adsorb. The descriptive parameters identified in our analysis reflect some of the criteria previously associated with catalyst performance and summarized in the “seven pillars of oxidation catalysis” (lattice oxygen, metal–oxygen bond strength, host structure, redox properties, multifunctionality of active sites, site isolation, and phase cooperation).^73,74^ However, the correlations captured by SISSO involve multiple physicochemical parameters related in a nonlinear way. These correlations were not identified in conventional studies. Thus, they provide insights beyond the established concepts in oxidation catalysis.

Finally, the AI analysis also enables to plan meaningful catalyst physical characterization campaigns, since it tells which techniques are the most crucial ones. Out of the 55 parameters initially considered in our study, only 6 appear in the expressions for the yields of the value-added products olefins and oxygenates. These key parameters were derived from N_2_ adsorption, XPS, and NAP-XPS techniques. However, there is always a combination of key parameters involved and these parameters might be related to each other. Therefore, the synthesis of an improved catalyst based on the knowledge gained by the approach presented here is still a challenging task. In this sense, the systematic inclusion of new data points in an active-learning fashion is a promising approach. Efficient experimental (automated) workflows for data generation and reliable estimates of AI model uncertainty will be crucial in order to efficiently navigate the materials space using such AI-guided experimental workflow.

## Conclusions

We have demonstrated how a clean-data-centric approach combining standardized experimental procedures, designed to capture the kinetics of active catalyst states formation from catalyst precursors, with interpretable AI enables the identification of key physicochemical parameters correlated with catalyst performance in selective oxidation. These correlations were determined for the oxidation of alkanes with different chain lengths under different reaction conditions. While the correlations reflect to some extent the previous empirical knowledge on the reactions and materials and thus provide a proof of principle of the method, they had not yet been identified in their full complexity in conventional studies. Large-scale energy storage and efficient material economic use of resources require the mastering of complex catalyzed reactions at interfaces. The integration of rigorously conducted experiments according to standard operating procedures and interpretable data analysis presented here points the direction to breaking the inherent barrier in developing better catalysts for such reactions.

## Supporting Information Available

Content: summary of relevant literature; description of catalyst synthesis procedures; sample IDs; details about the catalyst tests, probe reactions, and characterization techniques (CO oxidation, nitrogen adsorption, chemical analysis, XRD, TPR/O, XPS, and NAP-XPS); further details on the AI analysis; raw data of the catalyst test; raw data of CO oxidation; detailed description of the XRD characterization; additional tables and figures concerning analysis of surface area, textural properties, surface composition, and oxidation states, electronic properties, as well as further AI analysis results; references; link to handbook and raw data (PDF)

## Notes

The handbook describing the protocols for catalyst synthesis, characterization, and testing and the raw data is available in the repository of the Department of Inorganic Chemistry at the Fritz-Haber-Institut (https://ac.archive.fhi.mpg.de/P51850). The AI analysis described in this paper can be reproduced and modified at the Novel-Materials Discovery (NOMAD) AI Toolkit (https://nomad-lab.eu/aitoolkit/cleandata_oxidation). The data set of measured physicochemical parameters and performance is available at the NOMAD repository (DOI: 10.17172/NOMAD/2022.12.14-1)
