# Supplementary material for: Data-Centric Heterogeneous Catalysis: Identifying Rules and Materials Genes of Alkane Selective Oxidation
Source: J Am Chem Soc. 2023 Feb 6;145(6):3427–42. doi: 10.1021/jacs.2c11117 (PMC9936587; doi:10.1021/jacs.2c11117)
Supplement: Supplementary file 1 — ja2c11117_si_001.pdf [file ja2c11117_si_001.pdf]

SUPPORTING INFORMATION FOR

Data-Centric Heterogeneous Catalysis: Identifying Rules and Materials Genes of Alkane Selective Oxidation

Lucas Foppa[1*], Frederik Rüther[2], Michael Geske[2], Gregor Koch[3], Frank Girgsdies[3], Pierre Kube[3], Spencer J. Carey[3], Michael Hävecker[3,4], Olaf Timpe[3], Andrey V. Tarasov[3], Matthias Scheffler[1], Frank Rosowski[5,2], Robert Schlögl[3], and Annette Trunschke[3*]

[1]The NOMAD Laboratory at the Fritz-Haber-Institut of the Max-Planck-Gesellschaft and IRIS-Adlershof of the Humboldt-Universität zu Berlin, Faradayweg 4-6, D-14195 Berlin, Germany. [2]BasCat - UniCat BASF JointLab, Hardenbergstraße 36, D-10623 Berlin, Germany. [3]Department of Inorganic Chemistry, Fritz-Haber-Institut of the Max-Planck-Gesellschaft, Faradayweg 4-6, D-14195 Berlin, Germany. [4]Max Planck Institute for Chemical Energy Conversion, 45470 Mülheim, Germany. [5]BASF SE, Catalysis Research, Carl-Bosch-Straße 38, D-67065 Ludwigshafen, Germany.

*foppa@fhi-berlin.mpg.de https://orcid.org/0000-0003-3002-062X

*trunschke@fhi-berlin.mpg.de https://orcid.org/0000-0003-2869-0181

1. Content
2. Literature Review
3. Catalyst Synthesis Procedures
4. Functional Analysis and Ex Situ and In Situ Catalyst Characterization
5. Details on the Artificial Intelligence Analysis
6. Raw Data of Catalyst Test in Alkane Oxidation
7. Raw Data of Catalyst Test in CO Oxidation
8. Detailed Description of X-ray Diffraction Characterization
9. Analysis of Surface Area and Texture of Fresh and Activated Catalysts
10. Surface Analysis by Photoelectron Spectroscopy
11. Further Detailed Results of the SISSO Analysis
12. References
13. Link to Handbook and Raw Data: https://ac.archive.fhi.mpg.de/P51850

1. Literature Review

Table S1. Vanadium- and Manganese-based Catalysts Analyzed in this Work and Examples for Catalytic Reactions and Reaction Conditions Reported in the Literature.

catalyst	alkane	T_{react} [°C]	W/F or **τ** or GHSV	$C_x/O_2/H_2O$/inert	reference
$MoVO_X$	ethane	340	0.6 $g \cdot s \cdot ml^{-1}$	30/10/20/40	Katou 2004[1]
		280-380	0.6 $g \cdot s \cdot ml^{-1}$	10/10/00/80	Konya 2013[2]
		250-300	0.13–1.6 $g \cdot s \cdot ml^{-1}$	03/06/00/91	Wernbacher 2019[3]
		330-420	unclear	9.1/9.1/00/81.8	Melzer 2020[4]
	propane	250-360	0.75 $g \cdot s \cdot ml^{-1}$	7.5/10/45.5/37	Ishikawa 2014[5]
		250-300	0.36 $g \cdot s \cdot ml^{-1}$	03/06/40/51	Trunschke 2017[6]
		250-300	0.13–1.6 $g \cdot s \cdot ml^{-1}$	03/06/00/91	Wernbacher 2019[3]
	n-butane	250-300	0.13–1.6 $g \cdot s \cdot ml^{-1}$	03/06/00/91	Wernbacher 2019[3]
$MoVTeNbO_X$	ethane	340-400	70 $g\ h \cdot mol_{C2}^{-1}$	30/30/00/40	Lopéz Nieto 2002[7]
		400	unclear	04/08/00/88	Lopéz Nieto 2010[8]
	propane	390	1200 h^{-1}	05/10/47/38	Lin 2000[9]
		350	1.5 $g \cdot s \cdot ml^{-1}$	6.5/10/45 /38.5	Katou 2004[1]
		400	unclear	04/08/00/88	Lopéz Nieto 2010[8]
	n-butane	400	unclear	04/08/00/88	Lopéz Nieto 2010[8]
		400	16980 h^{-1}	03/06/00/91	Heine 2013[10]
$MnWO_4$	propane	400	0.9–2.4 $g \cdot s \cdot ml^{-1}$	10/05/00/85	Li 2019[11]
$SmMnO_3$	propane	200-300	0.2 $g \cdot s \cdot ml^{-1}$	10/05/00/85	Koch 2020[12]
V_2O_5	ethane	430-630	0.003 $g \cdot ml \cdot s^{-1}$	13/28/10/49	Oyama 1990[13]
	propane	360-400	unclear	1.8/76/22.2/00	Ai 1986[14]
	propane	500	unclear	04/08/00/88	Chaar 1988[15]
	n-butane	540	0.24 $g \cdot s \cdot ml^{-1}$	08/08/00/88	Chaar 1987[16]
$(VO)_2P_2O_7$ (VPP)	ethane	280-450	3.8 s	2.5/05/00/92.5	Merzouki 1993[17]
		300-500	0.02 - 7.5 $g \cdot s \cdot ml^{-1}$	10/05/00/85	Ivars-Barceló 2017[18]
	propane	360-400	unclear	1.8/76/22.2/00	Ai 1986[14]
		300-450	0.4 $g \cdot h \cdot dm^{-3}$	1.6/17.8/20/60.6	Landi 2005[19]
	n-butane	340-450	1000 h^{-1}	unclear	Hutchings 1996[20]
		250-450	0.04-1.8 $g \cdot s \cdot ml^{-1}$	1.2/16.4/00/82.4	Zhang-Lin 1994[21]
		360-420	2775 $mL \cdot g^{-1} \cdot h^{-1}$	02/20/03/75	Schulz 2019[22]
β-$VOPO_4$	*n*-butane	440	670 $g \cdot h \cdot mol^{-1}$	1.5/17/00/81.5	Schimoda 1985[23]
		250-450	0.04-1.8 $g \cdot s \cdot ml^{-1}$	1.2/16.4/00/82.4	Zhang-Lin 1994[21]
α_{II}-$VWOPO_4$	*n*-butane	375-450	2000 h^{-1}	02/20/03/75	Schulz 2019[24]
α_{II}-$VOPO_4$	*n*-butane	250-450	0.04-1.8 $g \cdot s \cdot ml^{-1}$	1.2/16.4/00/82.4	Zhang-Lin 1994[21]
		440	670 $g\ h \cdot mol^{-1}$	1.5/17/00/81.5	Schimoda 1985[23]
		385	4240 cm^{-1}	1.4/20/00/78.6	Eichelbaum 2013[25]
$VOPO_4 \cdot 2H_2O$	*n*-butane	400	2500 h^{-1}	1.7/20.3/00/79.7	Grisel 2004[26]
$VWPO_x$	*n*-butane	375-450	2000 h^{-1}	02/20/03/75	Welker-Nieuwoudt[27]

2. Catalyst Synthesis Procedures

$MoVO_x$.[28] 116.31 g of ammonium heptamolybdate (0.65 mmol Mo, Merck, purity >99%, Lot: A578482622) were dissolved in 2.875 L deionized water, which was obtained from a laboratory purification system (MilliQ), and mixed with 37.26 g $VOSO_4$ (0.17 mmol V, Sigma-Aldrich, purity 97%, Lot: MKCB3411) dissolved in 375 mL water at $\vartheta = 30°C$. The mixture was then transferred into a fully automated autoclave (MED1852, Premex Reactor AG, Switzerland) made of Hastelloy HC22 with a total volume of 5 L and a maximal operational volume of 3.75 L. The mixture was heated to $\vartheta = 200°C$ at 1°C/min and kept isothermal for 17 h while stirring with a rate of 100 rpm (lidheating off). The resulting black solid was isolated by filtration (pore 4 frit), washed 2 times with H_2O and dryed at $\vartheta = 80°C$ for 16 h. 71.6 g of a black powder were obtained. The sample was washed with oxalic acid (0.25 mol/L, $\vartheta = 60$ °C, 30 min, 25mL/g) and H_2O and dried at $\vartheta = 80$ °C for 24 h. Subsequently, a thermal treatment in Ar was performed (heating rate 10°C/min, 400°C, 2h, flow rate 100 mL/min) in a rotating furnace (Xerion, Germany) in portions of 2 g each. The activated portions were combined after confirming reproducibility by XRD, nitrogen adsorption and chemical analysis, and pressed and sieved according to the handbook,[29] sieve fraction 100-200 micrometer.

$MoVTeNbO_x$.[30] 146.88 g of ammonium heptamolybdate (Merck, purity >99%, Lot: A578482622) were dissolved in 2175 mL of deionized water at 40 °C. 47.5 g of ammonium niobium oxalate (H.C. Starck GmbH, Lot: 20060802) were added to the solution at $\vartheta = 40$ °C. The mixture was then transferred into a fully automated autoclave (MED1852, Premex Reactor AG, Switzerland) made of Hastelloy HC22 with a total volume of 5 L and a maximal operational volume of 3.75 L and heated to $\vartheta = 175$ °C at 1°C/min while stirring with a rate of 100 rpm. After 3 h at $\vartheta = 175$ °C, an aqueous solution of $VOSO_4$ (53.06 g in 375 mL H_2O, Sigma-Aldrich, purity 97%, Lot: MKCB3411) was added at 40 mL/min. After another 1.75 h at $\vartheta = 175$ °C, an aqueous solution of $Te(OH)_6$ (44 g in 375 mL H_2O, TCI, purity>99%, Lot: Y2S2I-BP) was added. The isothermal step was continued for 17 h, then the solution was cooled to $\vartheta = 30$ °C at 1.6 °C/min. The product of the hydrothermal synthesis was filtered (pore 4 frit). The solid phase was washed with water and dried at $\vartheta = 80$ °C for 16 h. 133.4 g of a black powder were obtained. Subsequently, a thermal treatment in Ar was performed (heating rate 10°C/min, $\vartheta = 650°C$, 2h, flow rate 100 mL/min) in a rotating furnace (Xerion, Germany) in portions of 2 g each. The activated portions were combined after confirming reproducibility by XRD, nitrogen adsorption and chemical analysis, and pressed and sieved according to the handbook,[29] sieve fraction 100-200 micrometer.

$MnWO_4$.[31, 32] 17.15 g $Na_2WO_4*2H_2O$ (Carl Roth, purity > 98%, Lot: 313188601) were dissolved in 130 g of H_2O (pH=9.75), and 13.05 g $Mn(NO_3)2*4H_2O$ (Merck, purity > 99%, Lot: 0099371) were dissolved in 130 g of H_2O (pH=4.78) at $\vartheta = 30°C$ (15 min each). Then the two solutions were mixed (pH=8.52) and a white precipitate was formed. Next, 2 M NaOH (11.4 mL) was used to adjust the pH to 9.9 (duration 2.5 minutes) and the final mixture was transferred into a fully automated autoclave (MED1316, Premex Reactor AG, Switzerland). A pressure test was perfomed while stirring at 50 rpm and 10 bar (N_2) for about 12.5 min. Then, the temperature was raised at 1.6 °C/min to $\vartheta = 180$ °C and kept isothermal for 24 h while stirring at 50 rpm. The product of the hydrothermal synthesis (light brown slurry with a clear, transparent phase on top) was separated by centrifugation (8000 rpm, 10 min), washed two times with deionized H_2O and dried at $\vartheta = 80$ °C for 16 h. Subsequently, a thermal treatment in Ar was performed (heating rate 10°C/min, $\vartheta = 400°C$, 2h, flow rate 50 mL/min) in a rotating furnace (Xerion, Germany) in portions of 2 g each. The activated portions were combined after confirming reproducibility by XRD, nitrogen adsorption and chemical analysis, and pressed and sieved according to the handbook,[29] sieve fraction 100-200 micrometer.

$SmMnO_3$.[12, 33] A batch of 20 g was obtained by combining 4 small batches. Each smaller batch was synthesized according to the following procedure: 8.59 g of $Sm(NO_3)\cdot 3.6H_2O$ (Aldrich,99.9%, Lot MKCG1993, 30819) and 5.10 g of $Mn(NO_3)2\cdot 4H_2O$(Carl Roth, purity > 98%, ChargeNumber 46827460) were dissolved in 50 mL of distilled water.This was followed by addition of 3.92 g of glycine (Lancaster,purity > 99%, 200-272-2 Batch 10052309). The reaction mixture was stirred for 1 h and then transferred into an evaporation basin. The gel was dried for 16 h at $\vartheta = 150$ °C on a hot plate. Ignition was proceeded in a vertically installed steel tube at $\vartheta = 350$ °C. The united powders of the 4 smaller batches were transferred into a large rotating furnace reactor (Xerion, Germany). Thermal activation was performed while rotating at 6 rpm and heating at 5 °C/min to $\vartheta = 800$ °C and continue dwelling for 5 h in a stream of 200 mL/min consisting of 20% O_2/Ar. The dark brown powder obtained was pressed and sieved according to the handbook,[29] sieve fraction 100-200 micrometer.

V_2O_5. Divanadium pentoxide (V_2O_5, purity 99.6 %, Dallan Galaxy Metal Material Co. Ltd., CAS-Nr. 1314-62-1) was purchased. The powder was pressed and sieved according to the handbook,[29] sieve fraction 100-200 micrometer.

VPP.[34] Vanadyl(IV) pyrophosphate (VPP, $(VO)_2P_2O_7$) was synthesized according to patent literature following an organic route. In short, divanadium pentoxide (V_2O_5) as vanadium source was dissolved in isobutanol ($C_4H_{10}O$) and phosphoric acid (H_3PO_4) was added. The mixture was heated under reflux until the precursor was formed as precipitate, which was then filtered,

washed, dried, and tempered. The powder was pressed and sieved according to the handbook,[29] sieve fraction 100-200 micrometer.

a-VPP. The synthesis of vanadyl(IV) pyrophosphate (VPP, $(VO)_2P_2O_7$) in its amorphous phase was performed with the same approach, as described for VPP. The resulting precursor was filtered, washed, dried and pre-activated in wet lean air (5 % O_2, 50 % steam in N_2) at ϑ = 375 °C. The obtained powder was pressed and sieved according to the handbook, sieve fraction 100-200 micrometer.

β-$VOPO_4$[35] The sample was synthesized in one batch according to the following procedure: 24.46 g of ammonium metavanadate (NH_4VO_3, Alfa Aesar, Lot L32044) was used as vanadium-source and mixed with 20.56 g diammonium hydrogenphosphate ($(NH_4)_2HPO_4$, Roth, Lot 219284319). The mixture was then dissolved in 500 mL deionized water. 4 mL of nitric acid (HNO_3, Thermo Fisher Scientific Inc., Lot 1864022) was added as an oxidizer. The solution was evaporated till dryness at temperatures $\vartheta \leq$ 80 °C; while stirring at 300 rpm. The dry residue was then dried at ϑ = 120 °C for 16 hours. The obtained powder was exposed to multiple tempering steps (ϑ = 400 °C for 24 hours, ϑ = 500 °C for 24 hours, ϑ = 600 °C for 24 hours, ϑ = 725 °C for 24 hours). The obtained sample (30 g) with a yellow appearance was pressed and sieved according to the handbook,[29] sieve fraction 100-200 micrometer.

α-$(V_{0.8}W_{0.2})OPO_4$.[22, 35] The sample was synthesized via solution combustion synthesis (SCS) in several small batches according to the following procedure: For each batch, 1.49 g of ammonium metavanadate (NH_4VO_3, Alfa Aesar, Lot L32044) was used as vanadium- and 0.8 g of ammonium metatungstate ($(NH_4)_6W_{12}O_{39}$, Alfa Aesar, Lot S05E020) as tungsten-source, which were mixed with 2.1 g diammonium hydrogenphosphate ($(NH_4)_2HPO_4$, Roth, Lot 219284319). The mixture was dissolved in 80 mL deionized water followed by the addition of 3.58 g glycine ($C_2H_5NO_2$, Sigma, Lot SLBV5094) as chelator and fuel. Furthermore, 2 mL of nitric acid (HNO_3, Thermo Fisher Scientific Inc., Lot 1864022) was added as an oxidizer. The solution was evaporated till dryness at $\vartheta \leq$ 80 °C while stirring at 250 rpm. The dry residue was then ignited in a preheated furnace at ϑ = 400 °C for 15 minutes. Then a first tempering step at ϑ = 400 °C was performed for 24 hours resulting in 43 g powder, which was then exposed to multiple tempering steps (ϑ = 500 °C and ϑ = 600 °C for 24 hours each and ϑ = 700 °C for 48 h). The obtained powder with an green appearance was pressed and sieved according to the handbook,[29] sieve fraction 100-200 micrometer.

$V_{0.167}W_{0.5}P_{0.333}O_{2.5+x}$.[36] The sample represents a novel tungsten-phosphate with a ReO_3-like structure type. For a sufficient batch size, 2 batches were prepared. Each batch was synthesized according to the following procedure: 1.94 g of ammonium metavanadate (NH_4VO_3, Alfa Aesar, Lot L32044) was used as vanadium- and 12.54 g of ammonium metatungstate ($(NH_4)_6W_{12}O_{39}$, Alfa Aesar, Lot S05E020) as tungsten-source, which were mixed with 4.38 g diammonium hydrogenphosphate ($(NH_4)_2HPO_4$, Roth, Lot 219284319). The mixture was dissolved in 400 mL deionized water followed by the addition of 15.94 g glycine ($C_2H_5NO_2$, Sigma, Lot SLBV5094) as chelator and fuel and by 10 mL of nitric acid (HNO_3, Thermo Fisher Scientific Inc., Lot 1864022) as an oxidizer. The solution was evaporated till dryness at $\vartheta \leq$ 80 °C while stirring at 250 rpm. The dry residue was then ignited in a preheated furnace at ϑ = 400 °C for 15 minutes. Then a first tempering step at ϑ = 400 °C was performed for 16 hours. Two batches of 15 g powder each ($\sum$ = 30.3 g) were obtained. The batches were combined and exposed to multiple tempering steps (ϑ = 500 °C for 16 hours, ϑ = 650 °C for 36 hours). The obtained powder with an olive green appearance was pressed and sieved according to the handbook,[29] sieve fraction 100-200 micrometer.

$VOPO_4 \cdot 2H_2O$ and α_{II}-$VOPO_4$.[37, 38] The vanadiumorthophosphate with α_{II}-structure type was prepared by precipitation similar to literature synthesis-routes. 48.48 g divanadium pentoxide (V_2O_5, purity ≥ 99.6 %, Dallan Galaxy Metal Material Co. Ltd., CAS-Nr. 1314-62-1) was mixed with an aqueous solution of phosphoric acid (1.165 g distilled water, 170 mL H_3PO_4 65 %). The suspension was then boiled in an round bottom flask under reflux for 17 hours. The mixture turned yellow after 70 minutes. After cooling it down to room temperature while mixing, the suspension was filtered (P3) and washed three times with 200 mL deionized water and dried. Then, the precipitate was washed three times with 100 mL acetone and dried at ϑ = 100 °C resulting in $VOPO_4 \cdot 2H_2O$. To obtain the α_{II}-phase, 17 g of the sample was used as precursor and tempered at ϑ = 725 °C for 2.5 days. The two different powders with a lemon-yellow appearance were pressed and sieved according to the handbook,[29] sieve fraction 100-200 micrometer.

Each sample is uniquely identified by a sample number in order to unambiguously assign reproductions of the synthesis. Table S2 gives an overview of the sample numbers of the freshly synthesized samples as well as the samples after activation and after the catalytic test in C_2, C_3 and C_4 oxidation.

Table S2. Unique Sample Identifyers in the Database of the Inorganic Chemistry Department at the Fritz-Haber-Institut der Max-Planck-Gesellschaft.

	parent	C2 activated	C2 spent	C3 activated	C3 spent	C4 activated	C4 spent
$MoVO_x$	31012	34035	32972	31804	32196	34048	31660
$MoVTeNbO_x$	31652	34036	34229	31821	32197	34049	32357
$MnWO_4$	32024	34037	34231	32111	32198	34050	34227
$Sm_{0.95}MnO_3$	30869	34038	34230	31836	32199	34051	32358
V_2O_5	31034	34039	34228	31846	32050	34052	31696
VPP	31650	34040	32974	32083	32052	34053	32363
a-VPP	31401	34041	32975	32082	32200	34054	31698
β-$VOPO_4$	31620	34042	31719	31848	32056	34055	31702
α_{II}-$(V_{0.8}W_{0.2})OPO_4$	31747	34043	32978	31850	32053	34056	32364
α_{II}-$VOPO_4$	31915	34044	32977	32084	32201	34057	32365
$VOPO_4 \cdot 2H_2O$	31199	34045	32980	32081	32051	34058	32360
$V_{0.167}W_{0.5}P_{0.333}O_x$	31749	34046	32973	31851	32049	34059	32361

3. Functional Analysis and Ex Situ and In Situ Catalyst Characterization

The general catalyst characterization and testing procedures are described in the handbook ,[29] whose updated version is accessible via the link **https://ac.archive.fhi.mpg.de/P51850**. More details are given below. The parameters varied and the data measured in the kinetic analysis are summarized in Figure 3 in the main text, in Table S3 and in Figures S3-S14. The materials properties measured are listed in Table S4.

Oxidation of Ethane and *n*-Butane. The catalyst tests were performed using a commercial 8-fold parallel setup build by hte GmbH. Catalyst volumes of 0.7 ml were placed in the isothermal zone of stainless steel reactor tubes with a diameter of 7 mm (the exact catalyst mass is documented with the data set). The catalyst bed was fixed by two inert steatite fillings. Reactor temperatures were individually controlled between 225 and 450°C at atmospheric pressure. The input gas feed was equally distributed between the parallel reactors. The feed composition was balanced by nitrogen. Argon was used as an internal standard, which included possible gas expansion in the data evaluation. The flow rates controlled by multiple mass flow controllers (MFC, Smart Mass Flow DELTA, Brooks Instrument LLC) were set based on the GHSV and feed composition guidelines of the respective reaction according to the handbook using C_2H_6, C_2H_4, C_4H_{10} (purity 3.5), C_4H_8 (purity 2.0), O_2 (purity 5.0), Ar (purity 5.0) and N_2 (purity 5.0) (Westfalen AG, Air Liquide S.A.). The design of experiments was fixed using the handbook guidelines. A blank reactor filled with an inert material was used to monitor the exact input feed composition in parallel to the eight reactor measurements, that was applied as a reference in the calculations of conversion and selectivity. The outlet gas flows were consecutively analyzed using an online gas chromatography (GC) system (Two GC 7890 A, Agilent, column configurations including a combination of Restek RTX Wax, Agilent CP Volamin or Restek RTX-5, Agilent Doppel Saeule HR, HP-Plot8/Q+PT with a flame ionization (FID) and thermal conductivity detector (TCD) each). Based on the catalyst and the reactants, multiple products including MAN, acrylic acid, acetic acid, acetaldehyde, alkenes, alkanes, CO and CO_2, among others, together with N_2, O_2 and Ar were identified and quantified. Each measurement point of the procedure or setpoint was tested in five iteration GC measurements. The final data set includes the averaged performance parameters at each setpoint. The overall gas composition was checked by a closed calculated carbon balance. The evaluation of the raw GC data was done using the commercial software tool myhte™. The calculation was performed according to the handbook guidelines using the formulas given in the handbook.

Oxidation of Propane. The catalyst tests were carried out using three different setups: a single tube fixed-bed reactor setup (quartz reactor, ID = 8 mm), a setup with 8 fixed-bed tubular reactors in parallel (quartz reactors, ID = 8 mm) (ILS, Germany) and a setup with 10 fixed-bed tubular reactors in parallel (hastelloy reactors, ID = 2 mm) (ILS, Germany). The experiments were performed at atmospheric pressure and under steady state conditions. The catalyst bed was fixed in the isothermal zone of the corresponding reactor by two quartz wool plugs or inert ceramic frits, respectively. An appropriate volume of the catalyst was used to realize gas hourly space velocities from 1000 to 4000 h^{-1}. The exact catalyst mass is documented with the data set. The reactor temperatures were individually controlled between 225 and 450°C for the single-tube and the 10-fold parallel reactor and in two blocks of four reactors each for the 8-fold parallel reactor. The gasous reactant feed was mixed with mass flow controllers (EL-FLOW, Bronkhorst) using C_3H_8 (purity 3.5), C_3H_6 (purity 3.5), O_2 (purity 5.0), and N_2 (purity 5.0) (Westfalen AG). Steam was added to the gas flow through vaporizers. The design of experiments was fixed using the handbook guidelines. Gas analysis was done using 3 online gas chromatographs (Agilent 7890A) with an equal column configuration. A combination of Plot-Q and Plot-MoleSieve 5A columns, connected to a thermal conductivity detector (TCD), was used to analyse the permanent gases CO, CO_2, N_2, and O_2. A system of a FFAP and a Plot-Q column, connected to a flame ionization detector (FID) with upstream methanizer, was used to analyse hydrocarbons, oxygenates, CO, and CO_2. Each measurement point of the procedure or setpoint was tested in three to five iteration measurements. The final data set includes the averaged performance parameters at each setpoint. The overall gas composition was checked by a closed calculated carbon balance. The calculation was performed according to the handbook guidelines using the formulas given in the handbook.

Table S3. Kinetic Parameters of the Alkane Oxidation Experiments.

symbol	unit	description
	ethane oxidation	
$GHSV$	h^{-1}	gas hourly space velocity
T	°C	temperature
c_{ethane}	vol%	ethane feed composition
c_{oxygen}	vol%	oxygen feed composition
c_{water}	vol%	water feed composition
c_{ethylene}	vol%	ethylene feed composition
c_{inert}	vol%	inert feed composition
X_{ethane}	%	ethane conversion
X_{oxygen}	%	oxygen conversion
C_{balance}	%	carbon balance
r_{ethane}	$mmol \cdot g^{-1} \cdot h^{-1}$	rate of ethane consumption
r_{CO_2}	$mmol \cdot g^{-1} \cdot h^{-1}$	rate of CO_2 formation
S_{ethylene}	%	ethylene selectivity
$S_{\text{acetic acid}}$	%	acetic acid selectivity
S_{CO}	%	CO selectivity
S_{CO_2}	%	CO_2 selectivity
	propane oxidation	
$GHSV$	h^{-1}	gas hourly space velocity
T	°C	temperature
c_{propane}	vol%	propane feed composition
c_{oxygen}	vol%	oxygen feed composition
c_{water}	vol%	water feed composition
$c_{\text{propylene}}$	vol%	propylene feed composition
c_{inert}	vol%	inert feed composition
X_{propane}	%	propane conversion
X_{oxygen}	%	oxygen conversion
C_{balance}	%	carbon balance
r_{propane}	$mmol \cdot g^{-1} \cdot h^{-1}$	rate of propane consumption
r_{CO_2}	$mmol \cdot g^{-1} \cdot h^{-1}$	rate of CO_2 formation
S_{ethane}	%	ethane selectivity
S_{ethylene}	%	ethylene selectivity
$S_{\text{acetaldehyde}}$	%	acetaldehyde selectivity
$S_{\text{acetic acid}}$	%	acetic acid selectivity
S_{ethanol}	%	ethanol selectivity
$S_{\text{propylene}}$	%	propylene selectivity
$S_{\text{propionic acid}}$	%	propionic acid selectivity
$S_{\text{propionic aldehyde}}$	%	propionic aldehyde selectivity
$S_{\text{acrylic acid}}$	%	acrylic acid selectivity
S_{acrolein}	%	acrolein selectivity
S_{acetone}	%	acetone selectivity
$S_{2-\text{propanol}}$	%	2-propanol selectivity
$S_{1-\text{propanol}}$	%	1-propanol selectivity
$S_{\text{allyl alcohol}}$	%	allyl alcohol selectivity
$S_{n-\text{butane}}$	%	*n*-butane selectivity
S_{CO}	%	CO selectivity

S_{CO_2}	%	CO_2 selectivity
	n-butane oxidation	
$GHSV$	h^{-1}	gas hourly space velocity
T	°C	temperature
$c_{n-\text{butane}}$	vol%	*n*-butane feed composition
c_{oxygen}	vol%	oxygen feed composition
c_{water}	vol%	water feed composition
$c_{1-\text{butene}}$	vol%	1-butene feed composition
c_{inert}	vol%	inert feed composition
$X_{n-\text{butane}}$	%	*n*-butane conversion
X_{oxygen}	%	oxygen conversion
C_{balance}	%	carbon balance
$r_{n-\text{butane}}$	$mmol \cdot g^{-1} \cdot h^{-1}$	rate of *n*-butane consumption
r_{CO_2}	$mmol \cdot g^{-1} \cdot h^{-1}$	rate of CO_2 formation
S_{ethylene}	%	ethylene selectivity
$S_{\text{acetylene}}$	%	acetylene selectivity
$S_{\text{acetic acid}}$	%	acetic acid selectivity
$S_{\text{acetaldehyde}}$	%	acetaldehyde selectivity
$S_{\text{propylene}}$	%	propylene selectivity
S_{propyne}	%	propyne selectivity
$S_{\text{acrylic acid}}$	%	acrylic acid selectivity
S_{acrolein}	%	acrolein selectivity
$S_{1-\text{butene}}$	%	1-butene selectivity
S_{MAN}	%	maleic anhydride selectivity
S_{furan}	%	furan selectivity
$S_{2,5-\text{dihydrofuran}}$	%	2,5-dihydrofuran selectivity
S_{CO}	%	CO selectivity
S_{CO_2}	%	CO_2 selectivity

Table S4. Materials Properties and Parameters Measured for the 12 Catalysts with the Two Different Redox Active Elements (RAE) Vanadium and Manganese and the Three Reactions Analyzed in this Work (Ethane, Propane, and *n*-Butane Oxidation). These 55 Physicochemical Parameters are Used as Primary Features within the SISSO Approach.

technique	symbol	unit	description
	T	°C	reaction temperature[a]
fresh samples			
N_2 ads. (*ex situ*)	s_{fr}	$m^2 \cdot g^{-1}$	surface area per mass
	$V_{\mathrm{fr}}^{\mathrm{pore}}$	$cm^3 \cdot g^{-1}$	pore volume per mass
XRF (ex situ)	$x_{\mathrm{b,fr}}^{\mathrm{RAE}}$	fraction atom	RAE bulk atomic content
XPS (ex situ)	$x_{\mathrm{s,fr}}^{\mathrm{RAE}}$	fraction atom	RAE surface atomic content
	$x_{\mathrm{s,fr}}^{\mathrm{O}}$	fraction atom	O surface atomic content
	$x_{\mathrm{s,fr}}^{\mathrm{C}}$	fraction atom	C surface atomic content
	$\Omega_{\mathrm{s,fr}}^{\mathrm{RAE}}$	e	RAE surface oxidation state
	$a_{\mathrm{fr}}^{\mathrm{C-C}}$	fraction area	amount of C 1s C-C component
	$a_{\mathrm{fr}}^{\mathrm{C-O}}$	fraction area	amount of C 1s C-O component
	$a_{\mathrm{fr}}^{\mathrm{C=O}}$	fraction area	amount of C 1s C=O component
	$\lambda_{\mathrm{fr}}^{\mathrm{RAE}}$	nm	RAE inelastic mean free path
	$\lambda_{\mathrm{fr}}^{\mathrm{O}}$	nm	O inelastic mean free path
	$\lambda_{\mathrm{fr}}^{\mathrm{C}}$	nm	C inelastic mean free path
TPRO (*ex situ*)	$u_{\mathrm{m,fr}}^{\mathrm{O_2}}$	μmol $O_2 \cdot g^{-1}$	O_2 uptake per mass
	$u_{\mathrm{s,fr}}^{\mathrm{O_2}}$	μmol $O_2 \cdot m^{-2}$	O_2 uptake per surface area
activated samples[b]			
XRD (ex situ)	$V_{\mathrm{act}}^{\mathrm{cell}}$	$Å^3$	normalized unit cell volume
N_2 ads. (*ex situ*)	s_{act}	$m^2 \cdot g^{-1}$	surface area per mass
	$V_{\mathrm{act}}^{\mathrm{pore}}$	$cm^3 \cdot g^{-1}$	pore volume per mass
XPS (ex situ)	$x_{\mathrm{s,act}}^{\mathrm{RAE}}$	fraction atom	RAE surface atomic content
	$x_{\mathrm{s,act}}^{\mathrm{O}}$	fraction atom	O surface atomic content
	$x_{\mathrm{s,act}}^{\mathrm{C}}$	fraction atom	C surface atomic content
	$\Omega_{\mathrm{s,act}}^{\mathrm{RAE}}$	e	RAE surface oxidation state
	$a_{\mathrm{act}}^{\mathrm{C-C}}$	fraction area	amount of C 1s C-C component
	$a_{\mathrm{act}}^{\mathrm{C-O}}$	fraction area	amount of C 1s C-O component
	$a_{\mathrm{act}}^{\mathrm{C=O}}$	fraction area	amount of C 1s C=O component
	$\lambda_{\mathrm{act}}^{\mathrm{RAE}}$	nm	RAE inelastic mean free path
	$\lambda_{\mathrm{act}}^{\mathrm{O}}$	nm	O inelastic mean free path
	$\lambda_{\mathrm{act}}^{\mathrm{C}}$	nm	C inelastic mean free path
NAP-XPS (*in situ*)[c]	$x_{\mathrm{s,dry}}^{\mathrm{RAE}}$, $x_{\mathrm{s,wet}}^{\mathrm{RAE}}$, $x_{\mathrm{s,alkane}}^{\mathrm{RAE}}$	fraction atom	RAE surface atomic content
	$x_{\mathrm{s,dry}}^{\mathrm{O}}$, $x_{\mathrm{s,wet}}^{\mathrm{O}}$, $x_{\mathrm{s,alkane}}^{\mathrm{O}}$	fraction atom	O surface atomic content
	$\Omega_{\mathrm{s,dry}}^{\mathrm{RAE}}$, $\Omega_{\mathrm{s,wet}}^{\mathrm{RAE}}$, $\Omega_{\mathrm{s,alkane}}^{\mathrm{RAE}}$	e	RAE surface oxidation state
	VB_{dry}, VB_{wet}, VB_{alkane}	eV	valence band onset
	W_{dry}, W_{wet}	eV	work function
	$\lambda_{\mathrm{NAP}}^{\mathrm{RAE}}$	nm	RAE inelastic mean free path
	$\lambda_{\mathrm{NAP}}^{\mathrm{O}}$	nm	RAE inelastic mean free path
spent samples[b]			
XRD (ex situ)	$V_{\mathrm{spt}}^{\mathrm{cell}}$	$Å^3$	normalized unit cell volume
XPS (ex situ)	$x_{\mathrm{s,spt}}^{\mathrm{RAE}}$	fraction atom	RAE surface atomic content
	$x_{\mathrm{s,spt}}^{\mathrm{O}}$	fraction atom	O surface atomic content
	$x_{\mathrm{s,spt}}^{\mathrm{C}}$	fraction atom	C surface atomic content

$\Omega_{\mathrm{s,spt}}^{\mathrm{RAE}}$	e	RAE surface oxidation state
$a_{\mathrm{spt}}^{\mathrm{C-C}}$	fraction area	amount of C 1s C-C component
$a_{\mathrm{spt}}^{\mathrm{C-O}}$	fraction area	amount of C 1s C-O component
$a_{\mathrm{spt}}^{\mathrm{C=O}}$	fraction area	amount of C 1s C=O component
$\lambda_{\mathrm{spt}}^{\mathrm{RAE}}$	nm	RAE inelastic mean free path
$\lambda_{\mathrm{spt}}^{\mathrm{O}}$	nm	O inelastic mean free path
$\lambda_{\mathrm{spt}}^{\mathrm{C}}$	nm	C inelastic mean free path

[a] The temperature is only used as primary feature when modelling catalyst conversion at multiple temperatures. [b] Every parameter corresponds to a different value for each of the three considered reactions, as the activation procedures are reactions conditions are reaction-dependant. [c]*In situ* measurement under reaction conditions. The subscripts "dry", "wet" and "alkane" correspond to the three different reaction feeds applied at $\boldsymbol{T}_{\mathbf{ref}}$: dry feed, wet feed and alkane-rich feed, respectively.

CO Oxidation. CO oxidation measurements were carried out using a single tube fixed-bed reactor setup with a u-shaped quartz reactor at atmospheric pressure. An on-line gas analyzer (X-Stream, Emerson/Rosemount) was used for quantification of O_2, CO, CO_2 and H_2O. The temperature inside the catalyst bed was recorded with a K-type thermocouple. The reactant feed was composed of CO, O_2, and N_2 as diluent. The CO-gas line was equipped with a carbonyl trap (tube filled with SiC and heated to 300 °C) and a CO_2 trap. All catalysts were pressed, crushed and sieved to a particle size of 100 to 200 µm. An appropriate volume of catalyst was used to realize a space velocity of 60000 h^{-1} (based on the undiluted catalyst). Each catalyst was diluted with SiC (100 to 200 µm) with a ratio of 1:9. All measurements were performed with a heating rate of 1 K min^{-1}. Prior to the CO oxidation, every fresh catalyst was pretreated at 150°C for 2 hours in inert gas flow. All CO oxidation measurements were performed from 30 °C to 420°C. Heating and cooling was repeated 3 times in the same feed (3 cycles). Two different feeds were applied for the CO oxidation, namely: (A) 1 Vol.-% CO (purity 3.5, Westfalen AG) and 20 Vol.-% O_2 (purity 5.0, Westfalen AG) (ratio of 1:20) in inert gas and (B) 1 Vol.-% CO and 2 Vol.-% O_2 (ratio of 1:2) in inert gas. Experiments with feed (A) and (B) were separate experiments using fresh catalysts.

Nitrogen Adsorption. Surface area determination and analysis of porosity were performed at -196°C using the Autosorb-6B analyzer (Quantachrome) after outgassing the catalysts under vacuum for 16 h at 150 °C. All data treatments were performed using the Quantachrome Autosorb software package. The specific surface areas s_{fr} and s_{act} of fresh and activated catalyst, respectively, were calculated according to the multipoint Brunauer−Emmett− Teller method (BET) in the range 0.05 < p/p0 < 0.15 assuming a N_2 cross-sectional area of 16.2 Å^2. The total specific pore volume $\boldsymbol{V}_{\boldsymbol{fr}}^{\boldsymbol{pore}}$ and $\boldsymbol{V}_{\boldsymbol{act}}^{\boldsymbol{pore}}$ of fresh and activated catalysts, respectively, were calculated using the amount of adsorbed N_2 at p/p_0 = 0.99.

Chemical Analysis. The chemical composition of the catalysts was determined by X-ray fluorescence spectroscopy using a Bruker S4 Pioneer wavelength dispersive X-ray fluorescence spectrometer. For sample preparation, a mixture of 0.05g of the catalyst and 8.9 g of lithium tetraborate (>99.995%, Aldrich) was fused into a disk using an automated fusion machine (Vulcan 2MA, Fluxana). The binary oxides were used for calibration.

X-ray Diffraction. X-ray diffraction (XRD) patterns were measured on a Bruker AXS D8 ADVANCE Series II theta/theta diffractometer, using Ni-filtered Cu Kα radiation and a position-sensitive LynxEye silicon strip detector. Powder samples were measured as provided (sieve fraction), i.e. without additional grinding, using a low background Si single crystal sample holder with a cavity. The data were collected in Bragg-Brentano geometry with a fixed 0.3° divergence slit (goniometer radius 217.5 mm) between 5 and 100° 2θ with 0.02° stepsize and a nominal counting time of 1 second/step, leading to a total accumulation time of 192 seconds per data point (due to 192 active detector channels). Identification of secondary phases was performed using DIFFRAC.EVA software (Bruker AXS) combined with the latest edition of the PDF-4+ database. The diffraction data were then analyzed by Whole Powder Pattern Fitting using the Rietveld method implemented in the DIFFRAC.TOPAS software (V5, Bruker AXS). The necessary crystal structure models were taken from the ICSD database unless noted otherwise. While lattice parameters, peak profile parameters, phase fractions and, in some cases, preferred orientation were refined, the atomic coordinates were generally kept fixed.

Temperature-Programmed Oxidation (TPO) and Temperature-Programmed Reduction (TPR). Temperature-programmed reduction and oxidation cycles were performed using a custom-designed TPR/O setup in a U shaped fixed bed quartz reactor (OD=10 mm, WT=1 mm). Around 300-350 mg pressed and sieved sample (100-200 micrometer) was positioned between two quarzwoll layers supported on the quartz substrate. All samples were exposed to sequential TPO-TPR cycles to examine the oxygen exchange capacity up to 400°C and its reversibility according to the following conditions: heating rate 5 K/min, 2 h holding time at 400°C, flow rate 40 mL min^{-1}, 0.25% O_2 in He in TPO, 0.25% H_2 in Ar.

The atmosphere in the cooling segment was the same as in the previous heating segment. The H_2 and O_2 consumption was monitored with thermal conductivity and paramagnetic detectors, respectively, build into a multichannel X-stream Gasanalyser by Emerson GmbH. An IR detector downstream of the O_2 sensor (ABB EL1020) was used to detect CO_2 during oxidation and inert treatment. A tube containing molecular sieve A was installed upstream the TCD detector as a water trap. The O_2 and H_2 detectors were calibrated with certified calibration gas mixtures and controlled with a CuO standard. The controlled gas flow was set with mass flow controllers EL-Flow by Bronkhorst calibrated with a flowmeter Definer 220. The gases used in the experiments (Ar (99.999%) and He (99.999%), Westfalen AG) were purified with Oxysorb and Hydrosorb cartriges. The inert segment was used to remove adsorbed species accumulated by contact with the ambient atmosphere. Nevertheless the first TPO segment was accompanied by significant CO_2 evolution. Hence, only the second and third TPR/TPO cycles were considered for evaluation of the reversible oxygen release/uptake.

Laboratory X-ray Photoelectron Spectroscopy (XPS). XPS spectra were recorded at room temperature using non-monochromatized Al Kα (1486.6 eV) or Mg Kα (1253.6 eV) excitation and a hemispherical analyzer (Phoibos 150, SPECS). Instrument work functions were calibrated to give an Au $4f_{7/2}$ metallic gold binding energy (BE) of 83.95 eV, while the spectrometer dispersion was adjusted to give a BE of 932.63 eV for metallic Cu $2p_{3/2}$. Furthermore, the binding energy scale was calibrated by the standard Au $4f_{7/2}$ and Cu $2p_{3/2}$ procedure. To calculate the elemental composition, the theoretical cross sections from Yeh and Lindau,[39] the inelastic free path of the electrons from Tanuma, Powell and Penn,[40] and the transmission function of the analyzer were used.

Near-Ambient Pressure XPS (NAP-XPS). Ambient-pressure X-ray photoelectron spectroscopy measurements were performed at the BELChem facility at the synchrotron radiation light source BESSY II of the Helmholtz-Zentrum Berlin, Germany. The station is served by the UE56/2-PGM beamline as a monochromatic X-ray source. The details of the beamline layout and performance can be found elsewhere.[41] The home-built near-ambient pressure electron spectrometer was described in detail before.[42, 43] 15 mg of the power samples were pressed into self- sustaining pellets and placed in front of the entrance aperture (diameter: 300 μm) of the electron spectrometer. The gases where dosed via calibrated mass flow controllers to the XPS chamber. The total pressure was 250 Pa. Heating was provided by a NIR laser (LIMO GmbH) from the rear and the temperature was measured with a K-type thermocouple pressed onto the sample surface. A constant heating ramp of 5 K/min was applied. First, the samples were activated in oxygen, heated to 300°C in dry feed (9:3:20=O_2/Cx/He) and the set of spectra was measured. After that the temperature was increased to various levels corresponding to the temperature of 30 % conversion in the reactor tests ("T30", temperature range between 300°C and 450°C). The feed gases were sequentially switched from wet feed (9:3:20=O_2:Cx:H_2O), back to dry feed (9:3:20= O_2:Cx:He), and finally to Cx rich feed (3:9:20=O_2:Cx:He). During each treatment step a set of core level spectra, valence band spectra, and secondary electron cut-off spectra has been measured after an equilibration time of 30 min. Core level spectra were recorded with a constant kinetic energy of about 150 eV by adapting the photon energy accordingly. This results in probing the outermost surface region with an inelastic mean free path of approximately λ = 0.6 nm for these materials.[44, 45] The pass energy of the hemispherical analyzer was 20 eV, the exit slit of the beamline 180 μm. For the quantitative analysis of the core levels, atomic subshell photoionization cross sections and asymmetry parameters from numerical calculations by Yeh and Lindau were used taking the photon-energy-dependent photon flux into account.[39] The core-level spectra were deconvoluted to determine the oxidation state using Gaussian−Lorentzian product functions after subtracting a Shirley background with the CasaXPS software.[46] To determine the work function ϕ of the sample, the secondary electron cut-off was measured with a bias of −17.8 V applied to the sample at a photon energy of 100 eV, beamline exit slit of 20 μm – 40 μm, and a pass energy of 2 eV. The position of the cut-off was determined by a linear extrapolation between 20 and 80% of the maximum intensity. The valence band (VB) onset was likewise evaluated by a leading-edge extrapolation using a photon energy of 100 eV. The composition of the effluent gas from the XPS cell was analyzed with a Thermo Scientific TRACE 1300 gas chromatograph to evaluate conversion and product selectivity.

4. Details on the Artificial-Intelligence Analysis

We used the SISSO++ code[47] with 200 SIS-selected descriptors per dimension (D) for model identification via the SO. Ten different residuals per dimension were considered to select these descriptors in the case of models with $D > 1$. The following mathematical operators were used: addition, (absolute) difference, multiplication, division, exponential, power (2, 3 or 6), square and cubic roots, logarithm and absolute value. Because the size of the descriptor candidate space grows in a combinatorial way with the number of primary features and mathematical operators, we only used part of the initial primary features for the identification of descriptors with rung 3 ($q = 3$). This is to keep the descriptor identification computationally feasible. We chose the primary features that appear in the top-ranked descriptors at the optimal complexity identified considering only $q = 1$ and $q = 2$ (Table S5). For the case of models trained with fresh-catalyst data only, all 16 primary features were consider in the $q = 3$ analysis. The AI approach is discussed in further details in a previous contribution.[48]

Table S5. Primary Features Selected for SISSO Analysis with q=3.

target	(q, D)[a]	
X_{alkane}	(1,2)	$x^{\text{C}}_{\text{s,fr}}$, $x^{\text{C}}_{\text{s,spt}}$<br>$x^{\text{RAE}}_{\text{s,fr}}$, $x^{\text{RAE}}_{\text{s,act}}$, $x^{\text{RAE}}_{\text{s,spt}}$, $x^{\text{RAE}}_{\text{s,dry}}$, $x^{\text{RAE}}_{\text{s,wet}}$, $x^{\text{RAE}}_{\text{s,alkane}}$<br>$x^{\text{RAE}}_{\text{b,fr}}$<br>$a^{\text{C−O}}_{\text{act}}$<br>$u^{\text{O}_2}_{\text{m,fr}}$<br>$\Omega^{\text{RAE}}_{\text{s,fr}}$, $\Omega^{\text{RAE}}_{\text{s,act}}$, $\Omega^{\text{RAE}}_{\text{s,spt}}$, $\Omega^{\text{RAE}}_{\text{s,wet}}$
X_{alkane}[b]	(1,2)	T<br>$x^{\text{C}}_{\text{s,fr}}$, $x^{\text{C}}_{\text{s,spt}}$<br>$x^{\text{RAE}}_{\text{s,fr}}$, $x^{\text{RAE}}_{\text{s,act}}$, $x^{\text{O}}_{\text{s,act}}$<br>$u^{\text{O}_2}_{\text{m,fr}}$<br>$\Omega^{\text{RAE}}_{\text{s,fr}}$, $\Omega^{\text{RAE}}_{\text{s,act}}$, $\Omega^{\text{RAE}}_{\text{s,spt}}$, $x^{\text{RAE}}_{\text{s,spt}}$<br>$x^{\text{RAE}}_{\text{b,fr}}$<br>$a^{\text{C−O}}_{\text{act}}$, $a^{\text{C−O}}_{\text{fr}}$<br>$\lambda^{\text{RAE}}_{\text{fr}}$
Y_{CO_x}	(1,2)	$x^{\text{RAE}}_{\text{s,fr}}$, $x^{\text{RAE}}_{\text{s,act}}$, $x^{\text{RAE}}_{\text{s,spt}}$, $x^{\text{RAE}}_{\text{s,wet}}$, $x^{\text{O}}_{\text{s,act}}$<br>$x^{\text{RAE}}_{\text{b,fr}}$<br>$x^{\text{C}}_{\text{s,fr}}$<br>$VB_{\text{dry}}$, VB_{wet}, VB_{alkane}<br>$\Omega^{\text{RAE}}_{\text{s,fr}}$, $\Omega^{\text{RAE}}_{\text{s,act}}$, $\Omega^{\text{RAE}}_{\text{s,spt}}$, $\Omega^{\text{RAE}}_{\text{s,dry}}$, $\Omega^{\text{RAE}}_{\text{s,wet}}$, $\Omega^{\text{RAE}}_{\text{s,alkane}}$
$\frac{Y_{\text{CO}_2}}{Y_{\text{CO}_x}}$	(1,2)	$V^{\text{pore}}_{\text{fr}}$, $V^{\text{pore}}_{\text{act}}$, s_{fr}<br>$\lambda^{\text{RAE}}_{\text{fr}}$<br>$\Omega^{\text{RAE}}_{\text{s,fr}}$, $\Omega^{\text{RAE}}_{\text{s,spt}}$, $\Omega^{\text{RAE}}_{\text{s,dry}}$, $\Omega^{\text{RAE}}_{\text{s,alkane}}$, $\Omega^{\text{RAE}}_{\text{s,wet}}$<br>$VB_{\text{wet}}$, VB_{alkane}<br>$x^{\text{C}}_{\text{s,spt}}$<br>$x^{\text{RAE}}_{\text{b,fr}}$<br>$a^{\text{C−O}}_{\text{act}}$
Y_{olefin}	(2,1)	s_{act}, $V^{\text{pore}}_{\text{act}}$, $V^{\text{pore}}_{\text{fr}}$<br>$\Omega^{\text{RAE}}_{\text{s,spt}}$, $\Omega^{\text{RAE}}_{\text{s,dry}}$<br>$VB_{\text{wet}}$, W_{wet}, W_{dry}<br>$\lambda^{\text{RAE}}_{\text{fr}}$, $\lambda^{\text{RAE}}_{\text{act}}$, $\lambda^{\text{C}}_{\text{spt}}$<br>$x^{\text{RAE}}_{\text{s,fr}}$, $x^{\text{RAE}}_{\text{s,act}}$, $x^{\text{C}}_{\text{s,act}}$, $x^{\text{C}}_{\text{s,fr}}$
$Y_{\text{oxygenate}}$	(1,1)	$x^{\text{C}}_{\text{s,act}}$, $x^{\text{O}}_{\text{s,fr}}$, $x^{\text{RAE}}_{\text{s,dry}}$, $x^{\text{RAE}}_{\text{s,wet}}$, $x^{\text{RAE}}_{\text{s,alkane}}$<br>$a^{\text{C−C}}_{\text{act}}$, $a^{\text{C−O}}_{\text{act}}$, $a^{\text{C=O}}_{\text{act}}$, $a^{\text{C−O}}_{\text{spt}}$, $a^{\text{C=O}}_{\text{spt}}$<br>$VB_{\text{dry}}$, VB_{wet}, VB_{alkane}, W_{dry}, W_{wet}
$S_{\text{oxygenate}}$	(1,1)	VB_{dry}, VB_{wet}, VB_{alkane}, W_{dry}, W_{wet}<br>$\Omega^{\text{RAE}}_{\text{s,fr}}$, $\Omega^{\text{RAE}}_{\text{s,dry}}$, $\Omega^{\text{RAE}}_{\text{s,wet}}$, $\Omega^{\text{RAE}}_{\text{s,alkane}}$, $\Omega^{\text{RAE}}_{\text{s,spt}}$<br>$a^{\text{C=O}}_{\text{fr}}$, $a^{\text{C=O}}_{\text{spt}}$<br>$x^{\text{C}}_{\text{s,act}}$, $x^{\text{C}}_{\text{s,spt}}$, $x^{\text{O}}_{\text{s,act}}$

[a] Optimal model complexity (q and D correspond to the depth of the symbolic-regression tree and to the descriptor dimension, respectively) identified by leave-one-material-out cross-validation considering only $q = 1$ and $q = 2$. [b]Without primary features derived from *in situ* characterization.

In order to evaluate the optimal model complexity, we performed leave-one-material-out cross-validation. This CV procedure consists of training models with a data set in which one of the catalysts is removed, and then using the so-obtained ensemble of best models to predict the property of the left-out material. This procedure is iterated until all the catalysts are left-out once. The root mean squared errors (RMSEs) averaged over all CV iterations (averaged CV-RMSEs) are used as our performance metric. The optimal complexity is considered the one with the lowest CV-RMSE. Because of the rather small number of materials and the fact that some materials might be unique compared the remaining ones (e.g., $MoVTeNbO_x$ in C_3 oxidation), the estimation of the target for the left-out material using the best model trained on the remaining materials might present abnormally low or high values, for example due to small denominators on the descriptor expression. This is particularly the case for higher-rung and higher-dimensional models. To circumvent this issue, we considered, for the evaluation of CV-RMSE, not only the best model, but rather the few N_{ensemble} best top-ranked models (ranked according to their training RMSE) when estimating the RMSE on the left-out (test) material. By doing this, we ensure a balance between the performance on the subset of data used for training and on the test material. The evolution of CV-RMSE as a function of N_{ensemble} (Fig. S1) shows that when only the model with the best performance in the training set is considered ($N_{\mathrm{ensemble}} = 1$), the CV-RMSEs might be extremely large for some models. When a few more models are considered in the ensemble, the errors drop significantly and are stabilized for ensemble sizes of ca. 25 for most of the cases. The CV-RMSE values shown in Fig. S2 correspond to $N_{\mathrm{ensemble}} = 25$. For the case of the target $Y_{\mathrm{oxygenate}}$, the CV-RMSE values for $q = 1$ and $q = 2$, 1-D models are very similar (1.89 and 2.03%, respectively, Fig. S2). Thus, both complexities could be considered optimal. We only discuss the $q = 2$, 1-D model in the manuscript based on the fact that the CV-RMSE is stabilized at lower N_{ensemble} values for $q = 2$ compared to $q = 1$ (Fig. S1). The descriptor associated to the best identified model for $Y_{\mathrm{oxygenate}}$ with the complexity 1-D, $q = 1$ model is $|VB_{\mathrm{alkane}} - W_{\mathrm{wet}}|$.

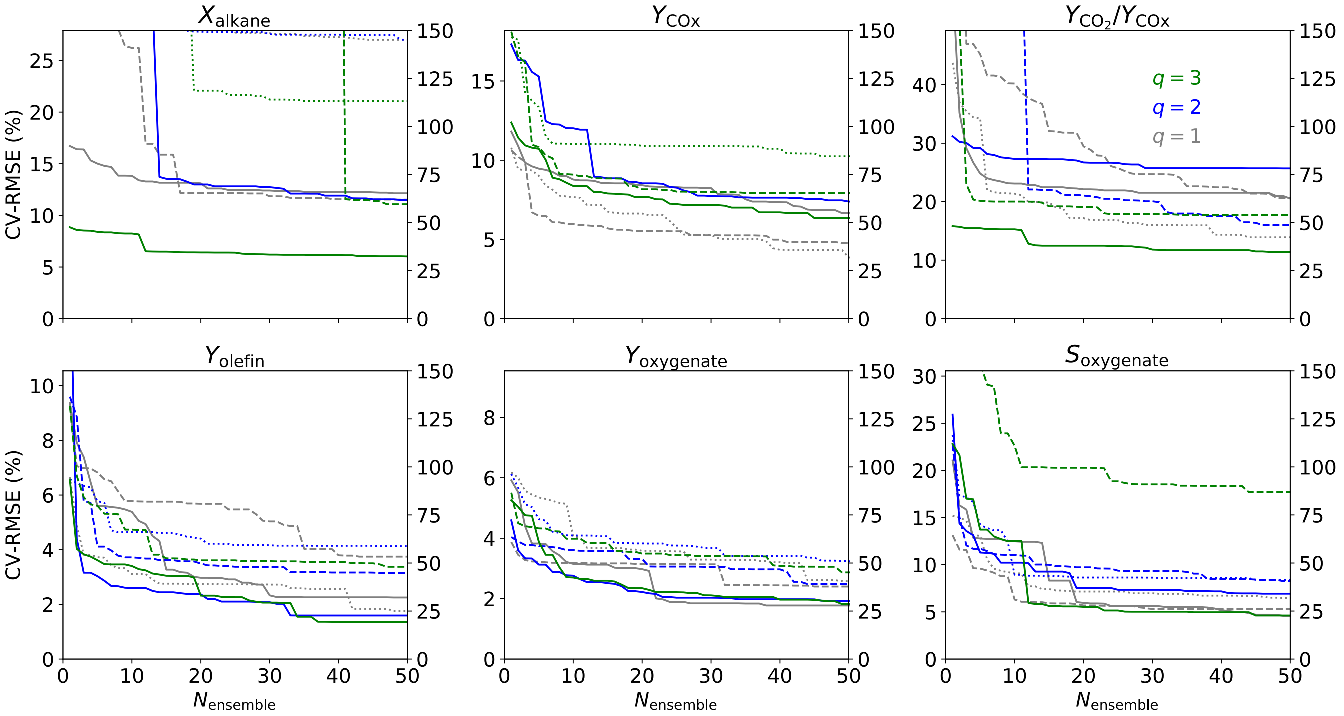

Figure S1. Average CV-RMSE dependence on the number of top-ranked descriptors analyzed (N_{ensemble}) for all the targets analyzed. The solid, dashed and dotted lines correspond, respectively, to 1-, 2-, and 3-D models.

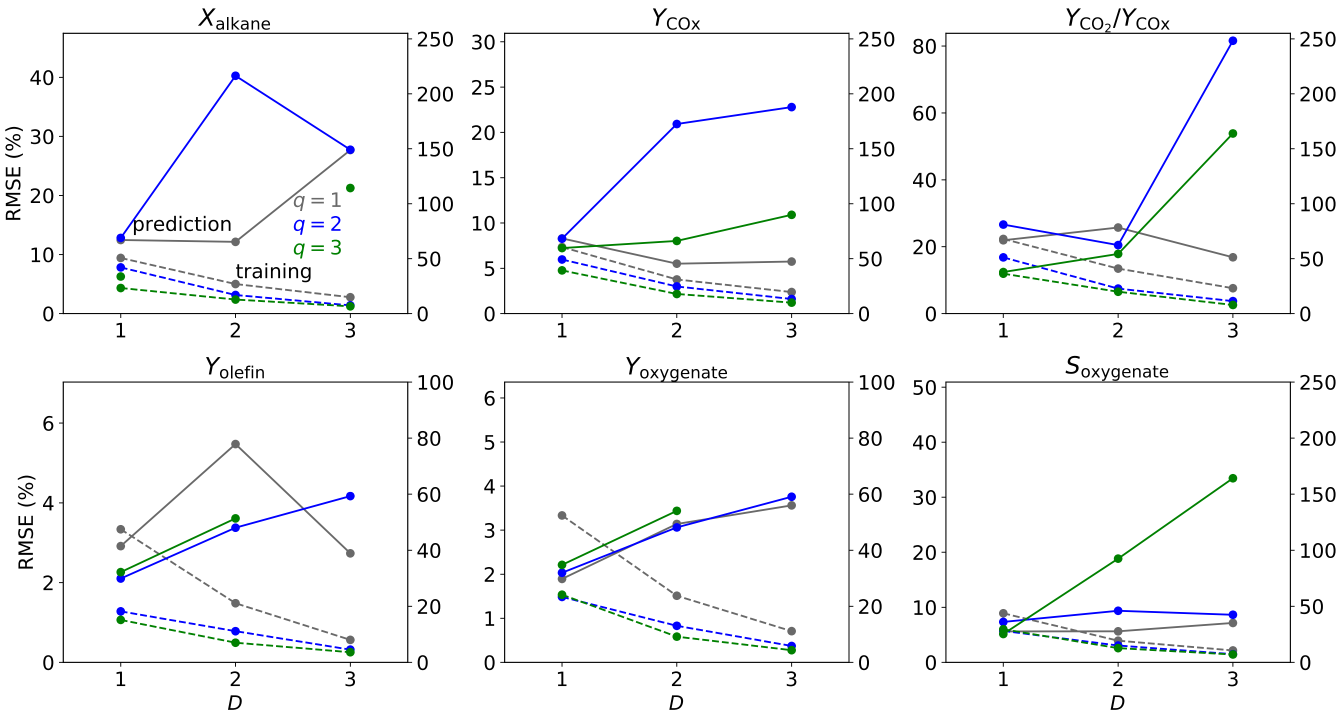

Figure S2. CV analysis of models derived by MT-SISSO for the analyzed targets. The CV errors shown correspond to the averaged RMSE across leave-one-material-out-CV iterations. The secondary axis (on the right) shows the CV-RMSE as a fraction of the standard deviation of the target over the whole dataset. The CV-RMSE values shown here correspond to $N_{\text{ensemble}} = 25$.

5. Raw Data of Catalyst Test in Alkane Oxidation

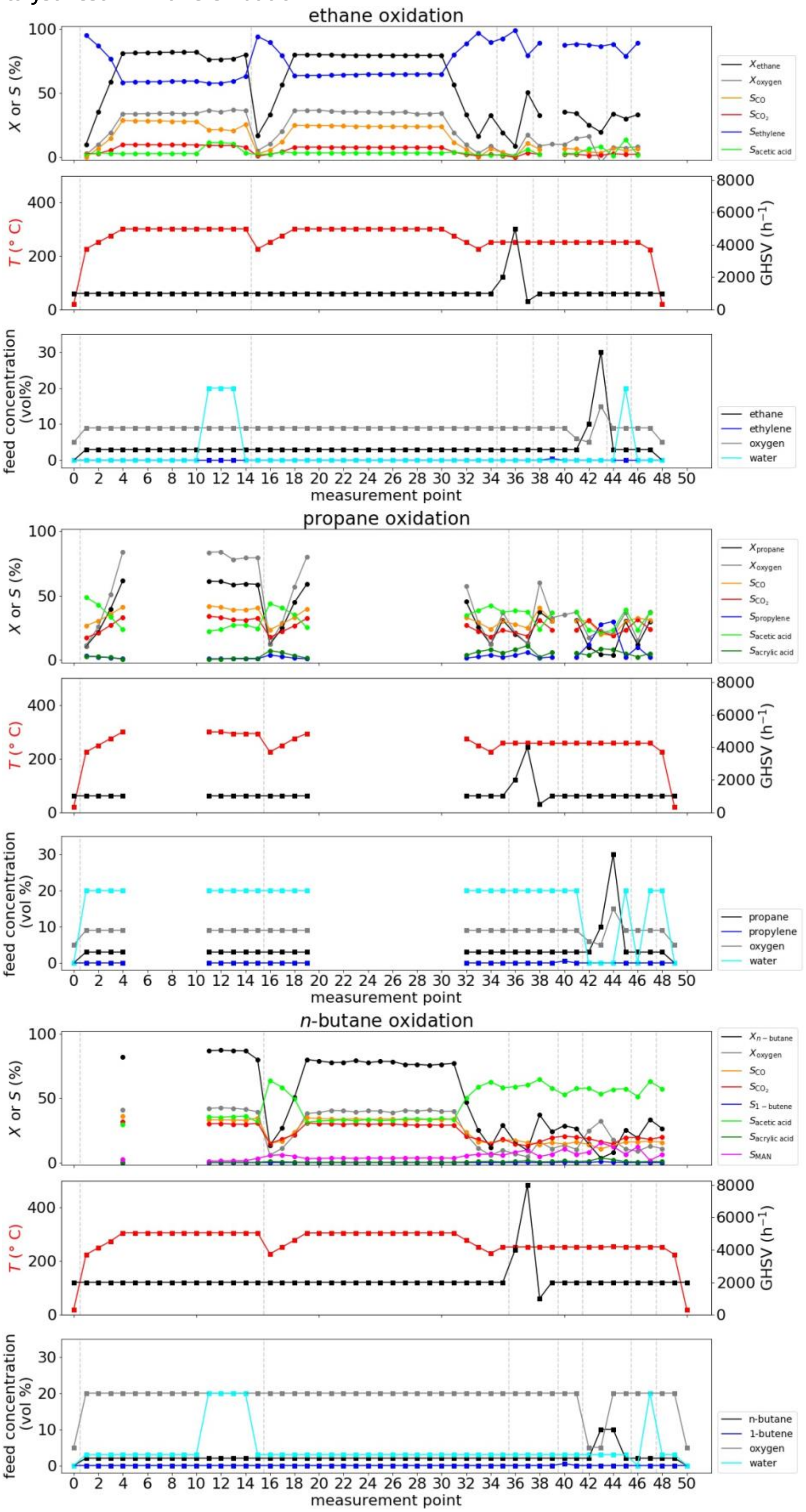

Figure S3. Catalyst test for $MoVO_x$.

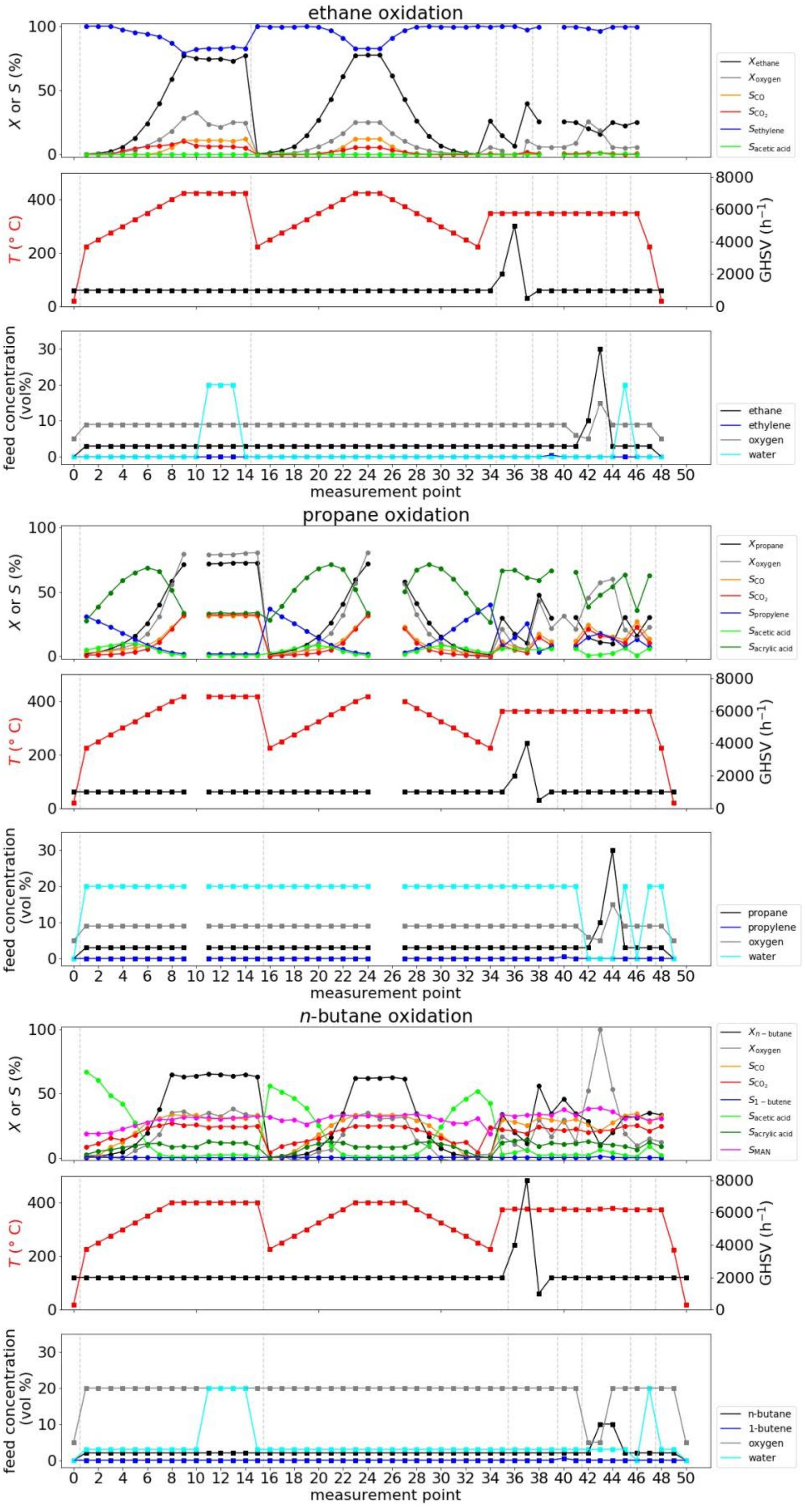

Figure S4. Catalyst test for the $MoVTeNbO_X$.

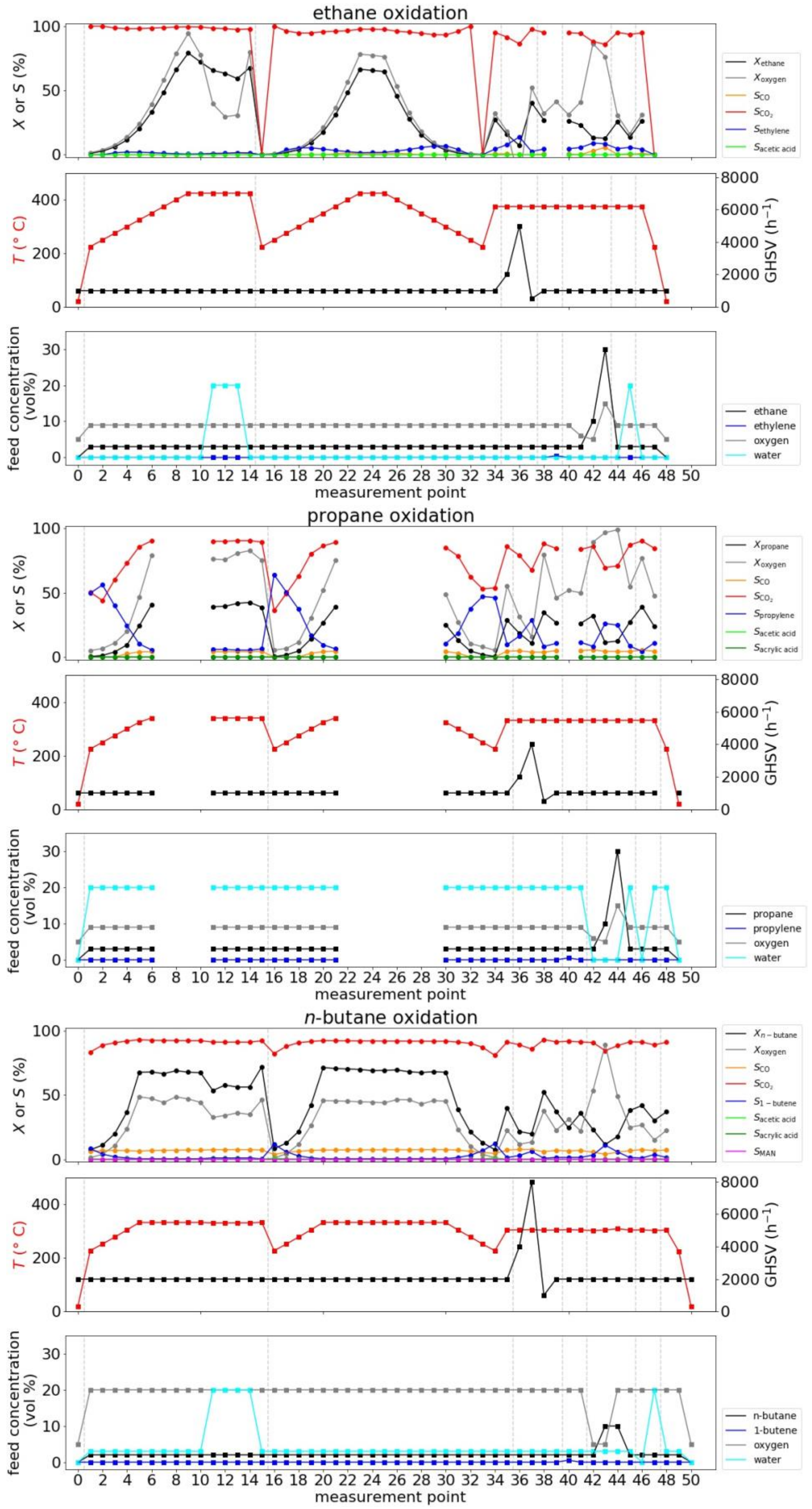

Figure S5. Catalyst test for $MnWO_4$.

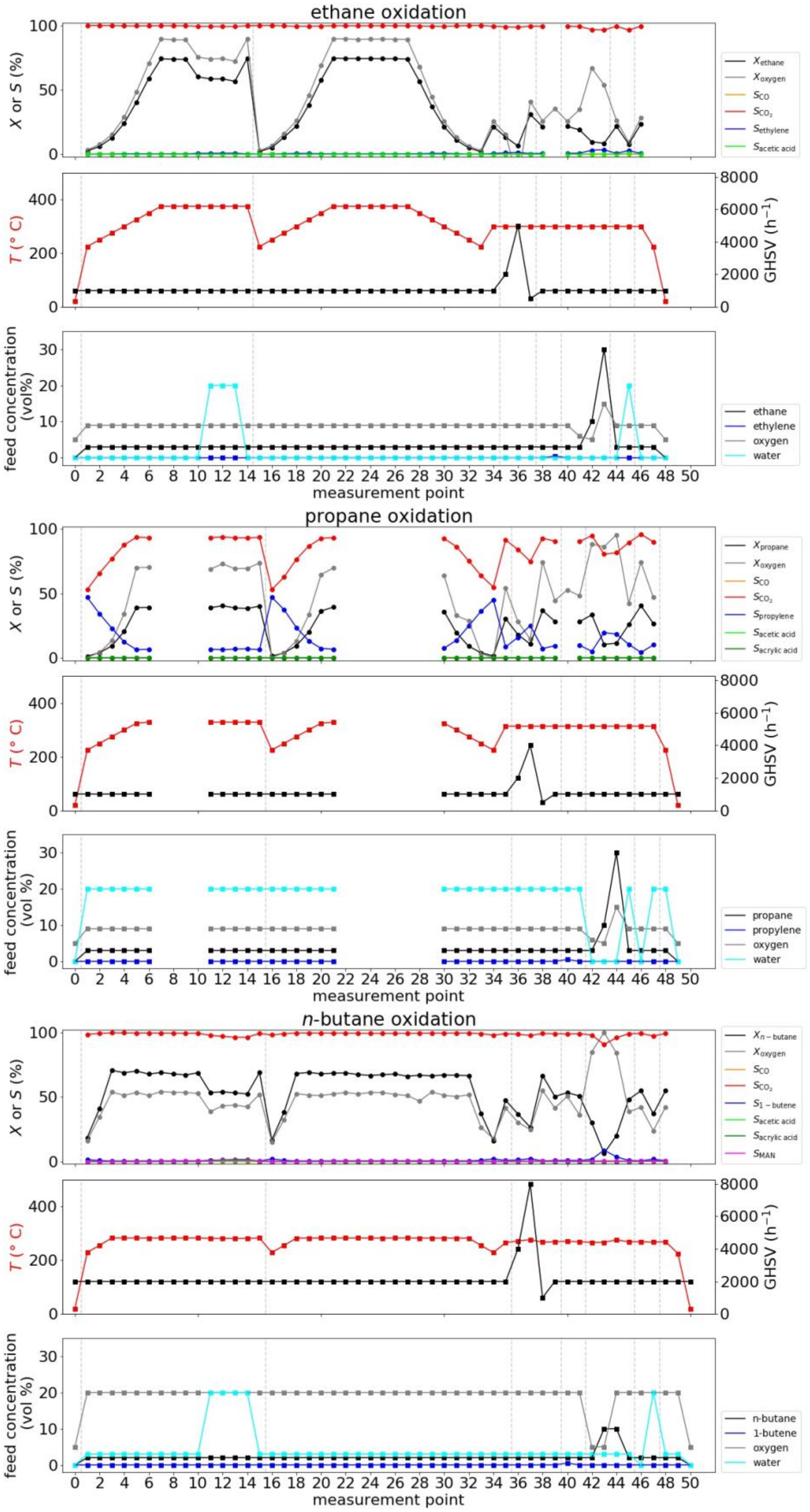

Figure S6. Catalyst test for $SmMnO_3$.

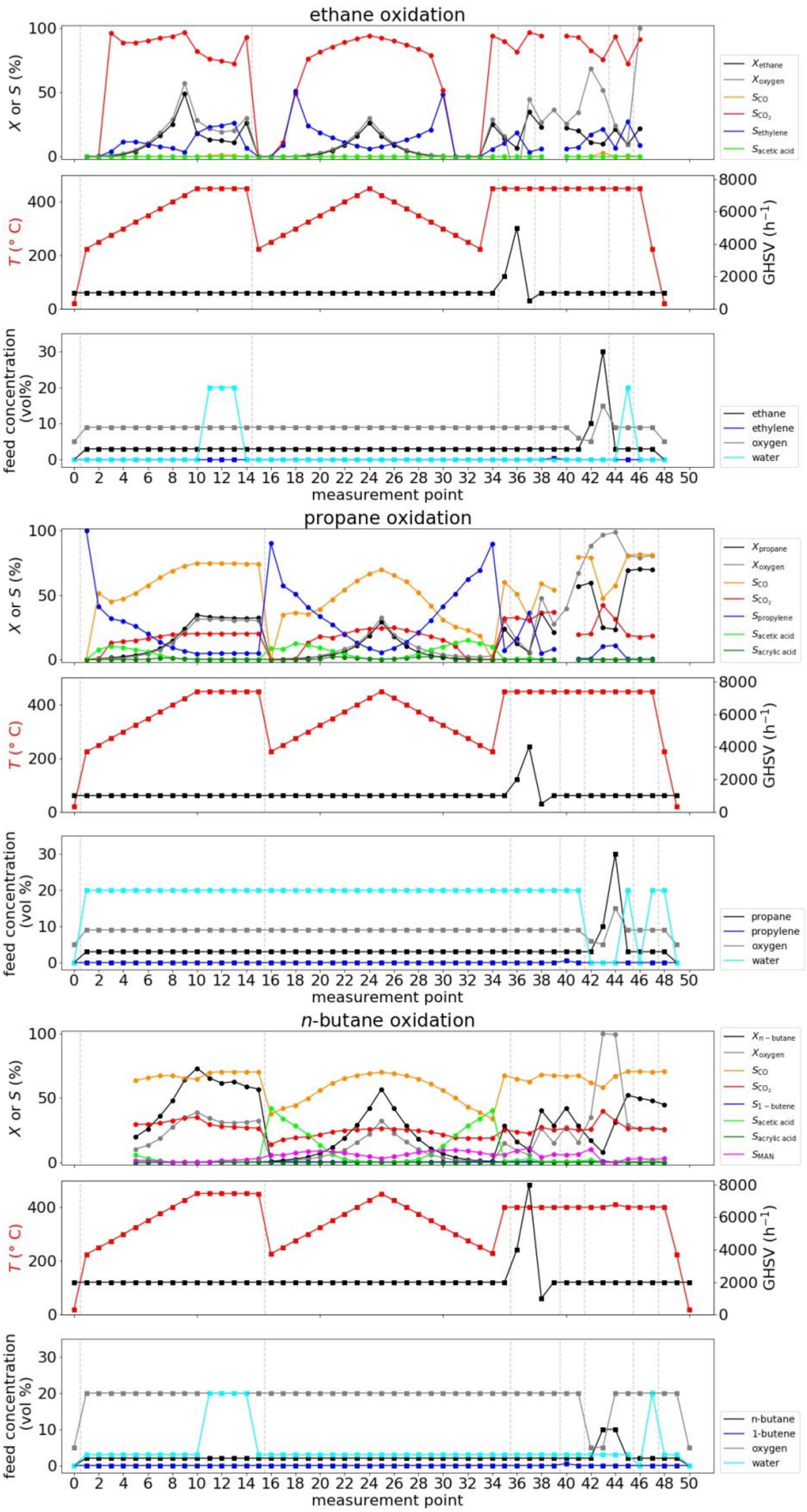

Figure S7. Catalyst test for V_2O_5.

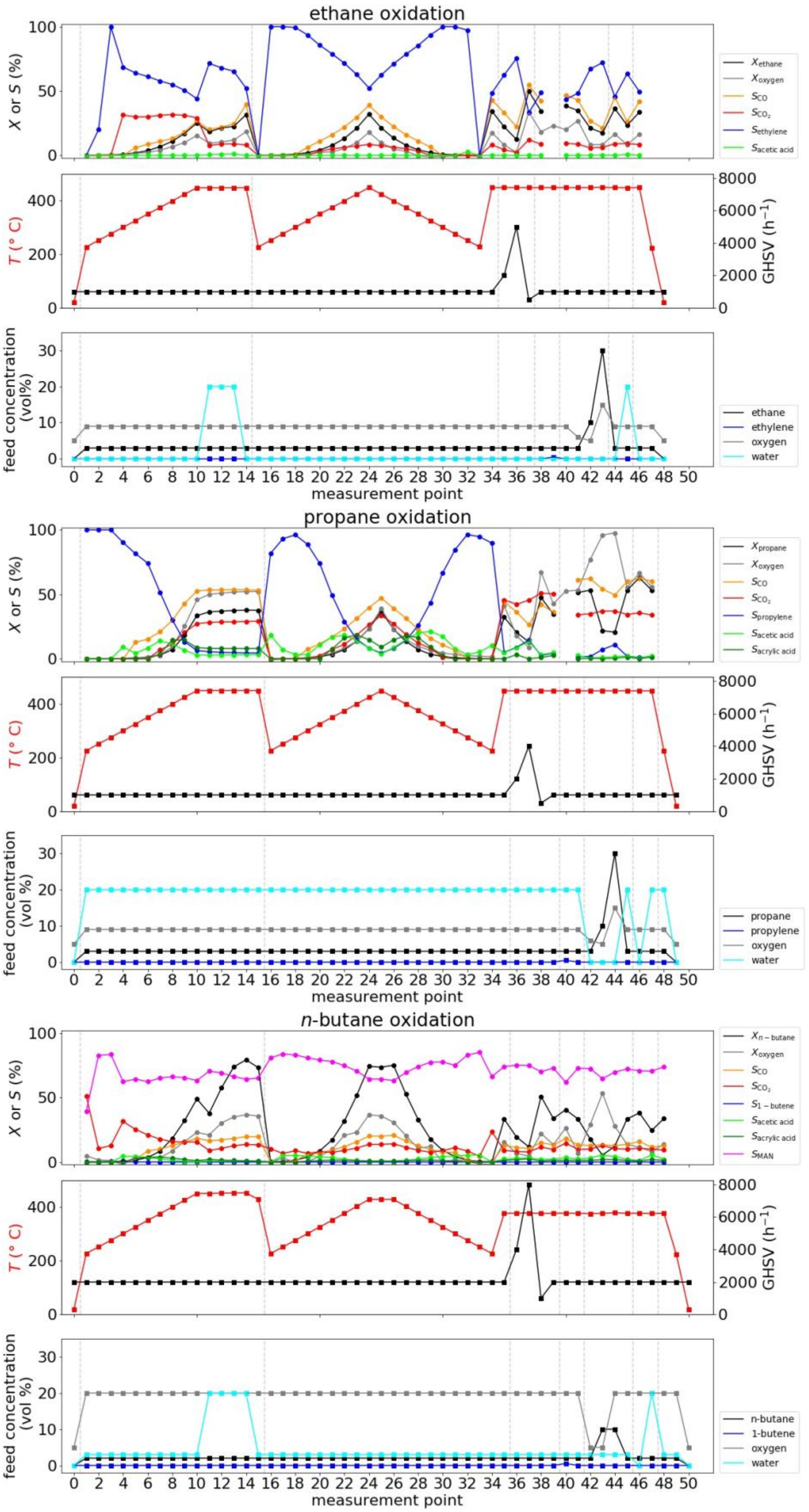

Figure S8. Catalyst test for $(VO)_2P_2O_7$ (VPP).

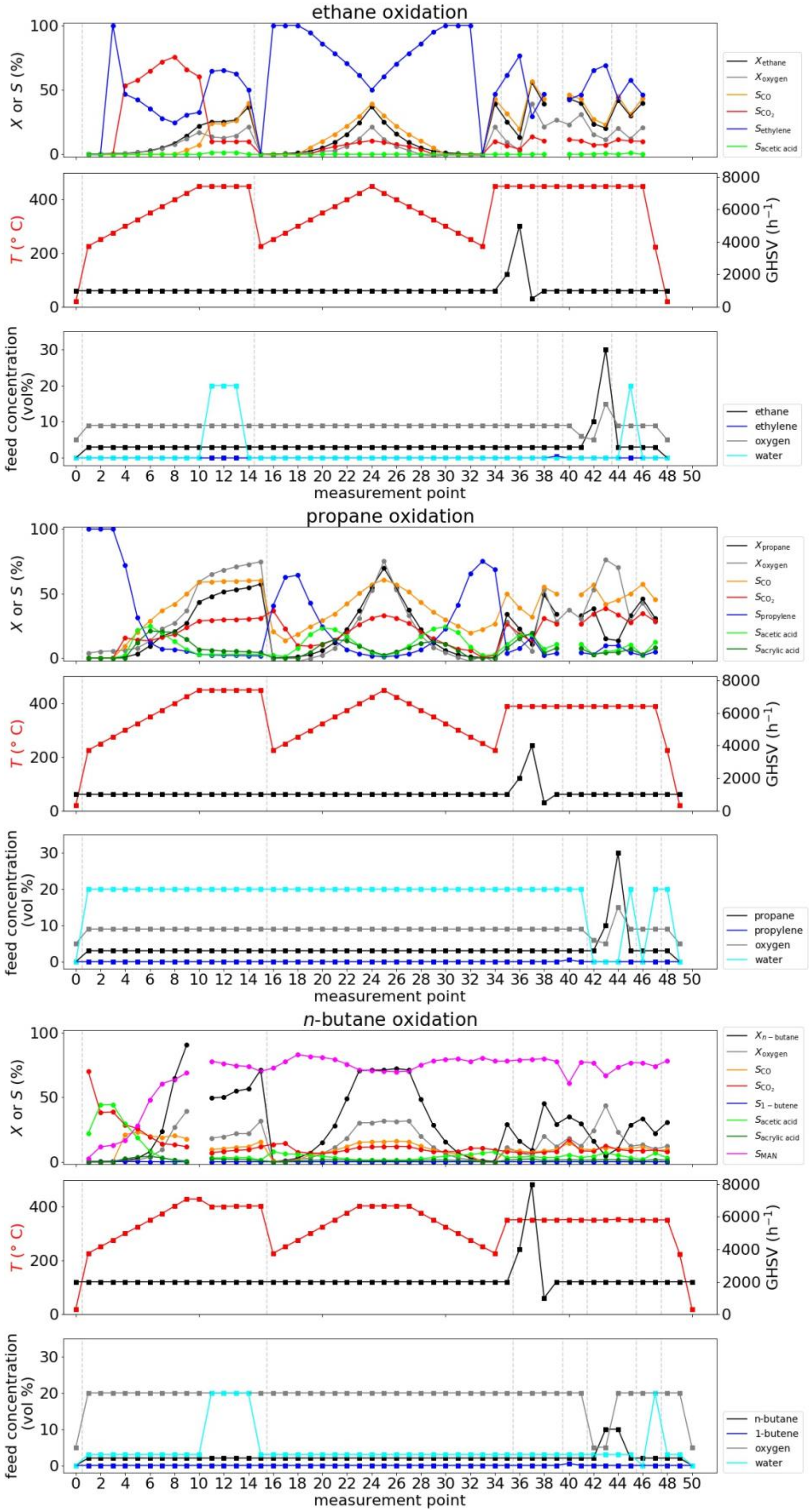

Figure S9. Catalyst test for amorphous VPP.

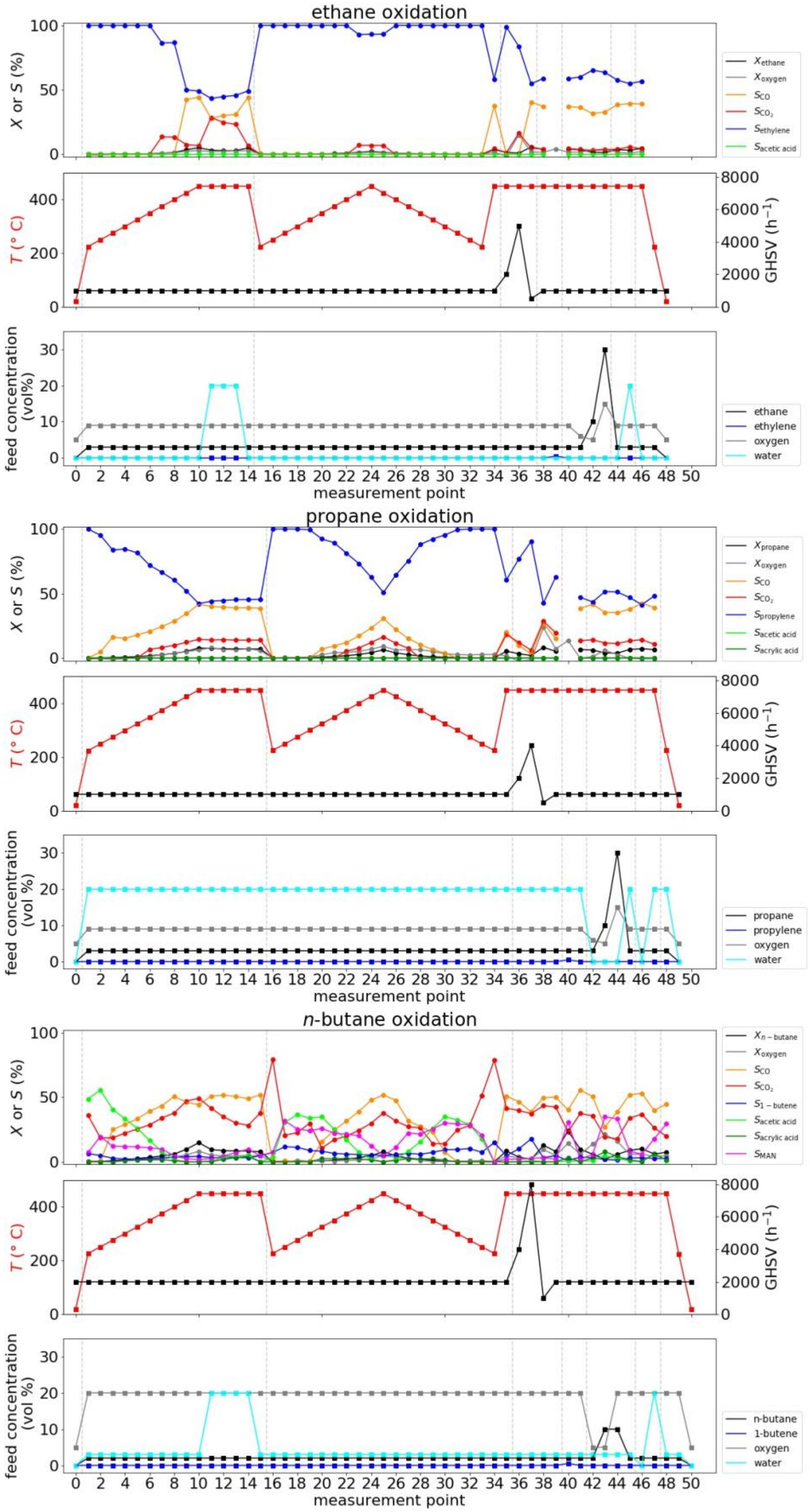

Figure S10. Catalyst test for β-$VOPO_4$.

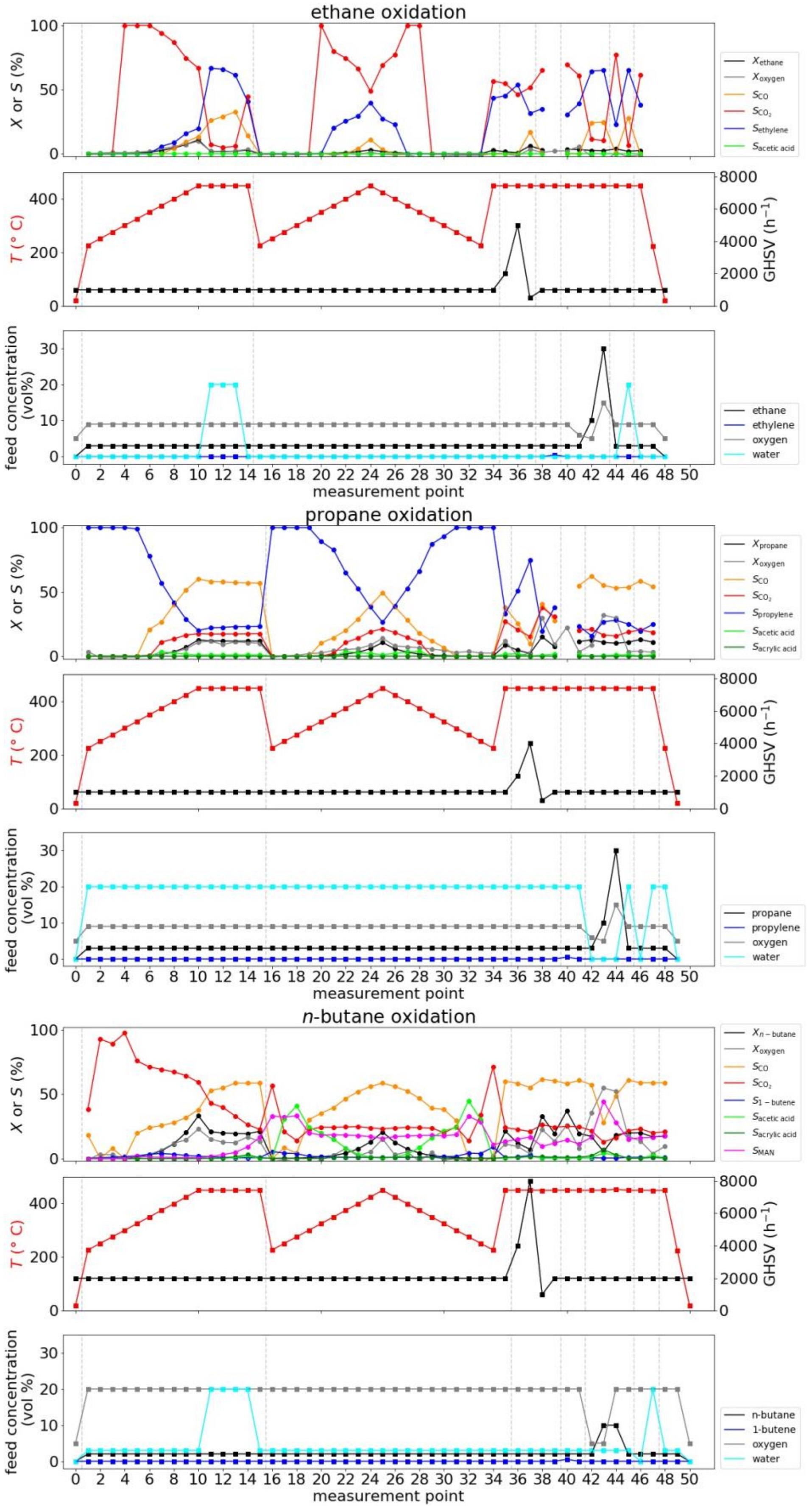

Figure S11. Catalyst test for α_{II}-VWOPO$_4$.

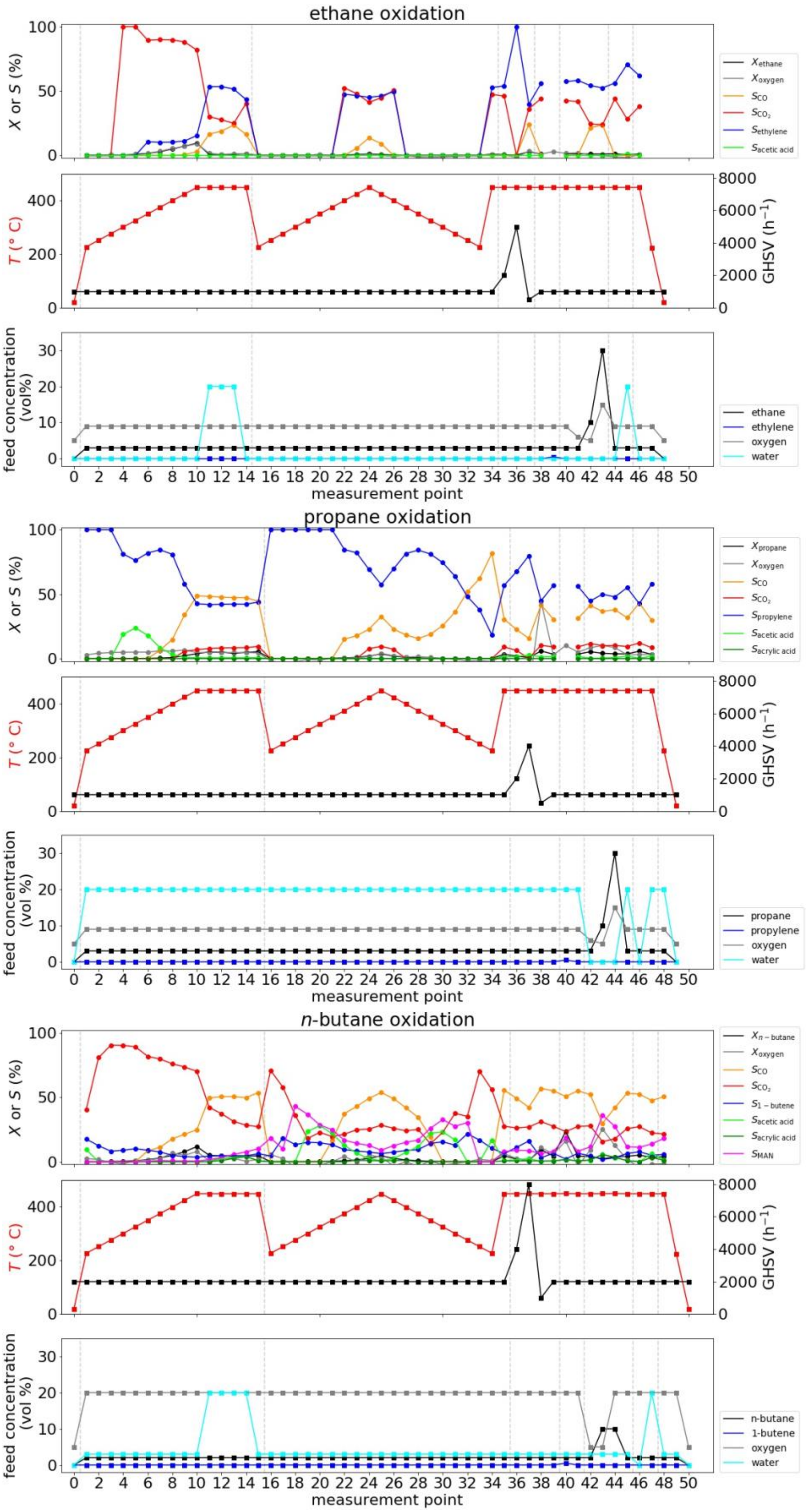

Figure S12. Catalyst test for α_{II}-$VOPO_4$.

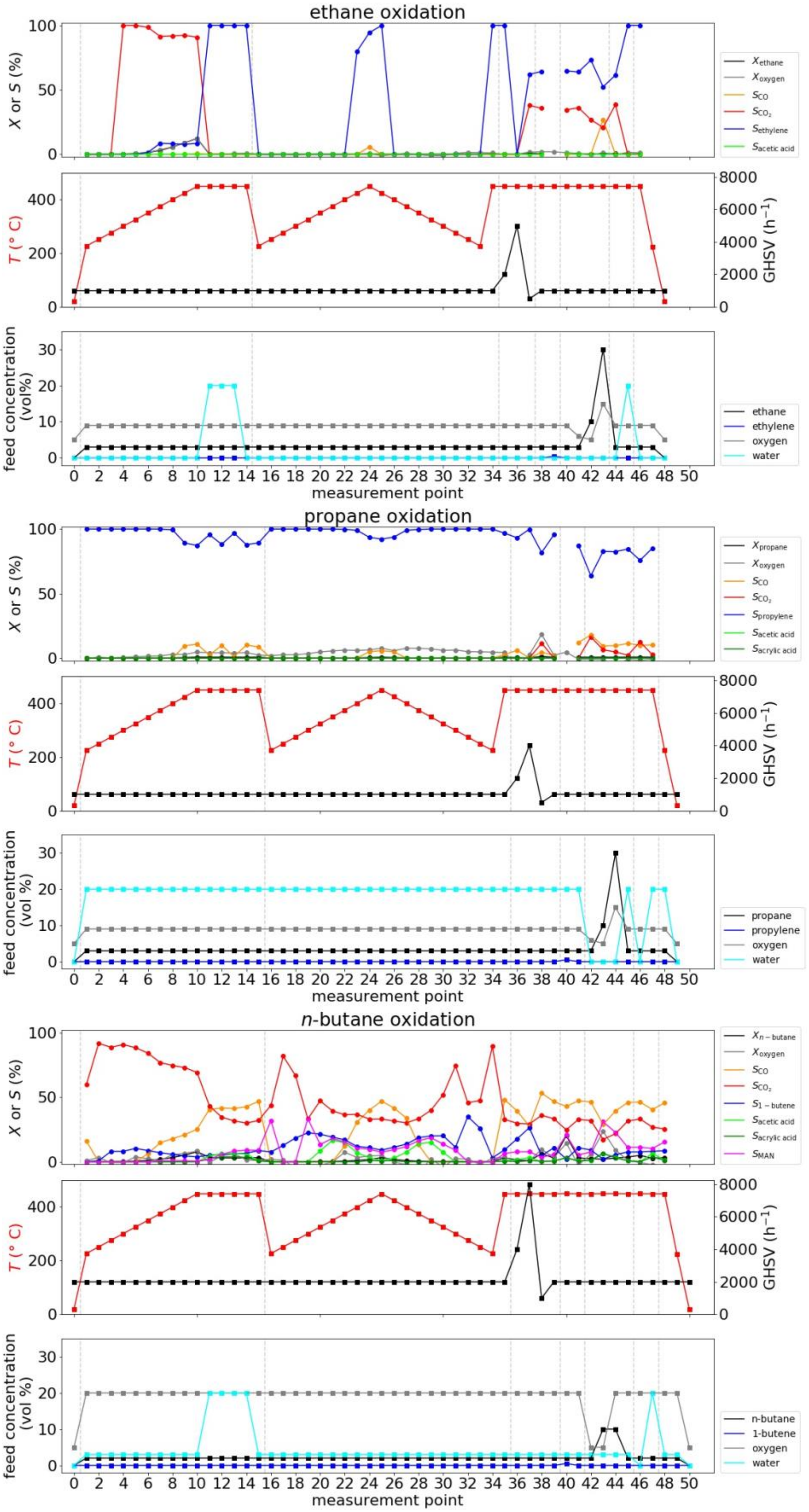

Figure S13. Catalyst test for $VOPO_4 \cdot 2H_2O$.

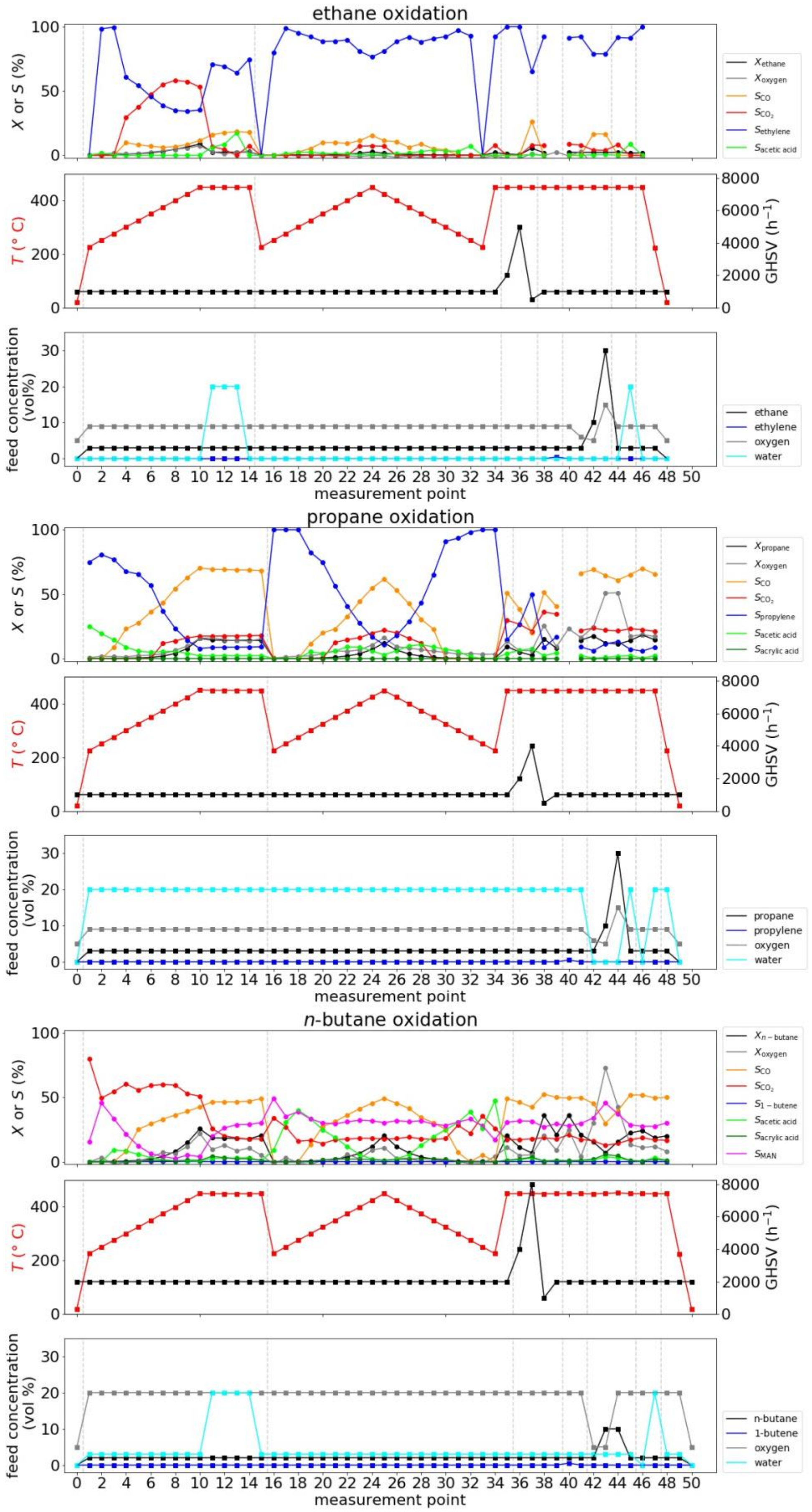

Figure S14. Catalyst test for $VWPO_x$.

6. Raw Data of the Catalyst Test in CO Oxidation

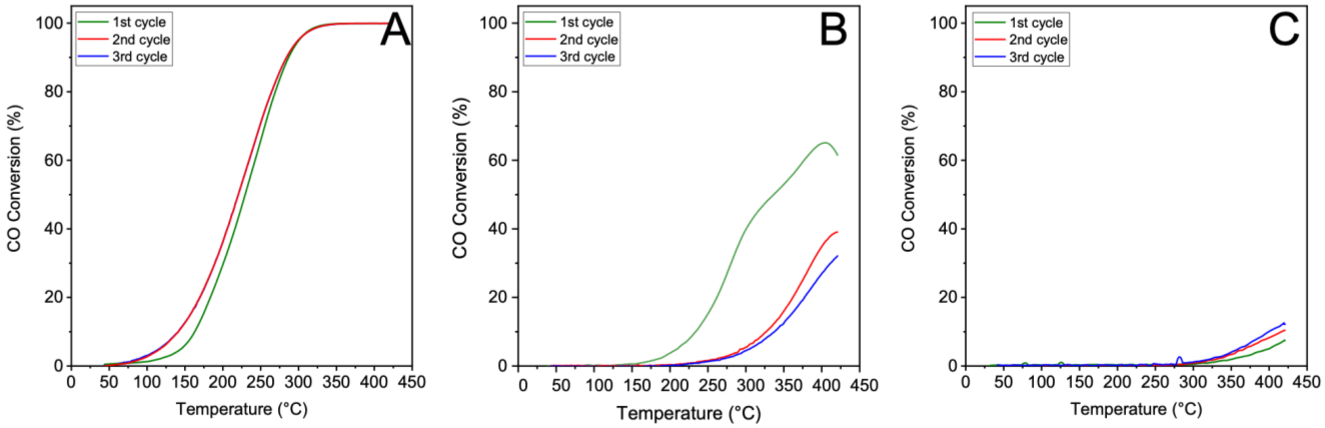

Figure S15. Light-off curves of (A) $SmMnO_3$, (B) $MnWO_4$, and (C) α_{II}-$VWOPO_4$ in CO oxidation in oxygen rich feed $CO:O_2:N_2$ = 1:20:79 vol.%.

7. Detailed Description of X-Ray Diffraction Characterization

The crystal structures and peak shape models used in the whole powder pattern fitting according to the Rietveld method are listed in Table S6.

Table S6. Crystal Structure and Peak Broadening Models Used for XRD Analysis.

catalyst	structure type (ICSD)	structure model	space group	size [a]	strain [a]
$MoVO_x$	$(TeO)_{1-d}((Mo_{9-x}V_xNb)O_{28})$	[6]	Pba2 (32)	i L [b]<br>i LG [c]	-
$MoVTeNbO_x$	$(TeO)_{1-d}((Mo_{9-x}V_xNb)O_{28})$	ICSD 55097[49]	Pba2 (32)	a L	i G
$MnWO_4$	Wolframite#$NiWO_4$	ICSD 15850[50]	P2/c (13)	a LG	-
$SmMnO_3$	Perovskite#$GdFeO_3$	ICSD 183040[51]	Pbnm (62)	i LG	a LG
V_2O_5	V_2O_5	ICSD 60767[52]	Pmmn (59)	a LG	
VPP	$(VO)_2P_2O_7$	ICSD 88661[53]	$Pca2_1$ (29)	a L	i G
amorphous VPP	-	-	-	-	-
β-$VOPO_4$	β−$VOSO_4$	ICSD 291605[54]	Pnma (62)	i L	i G
α_{II}-$VWOPO_4$	$MoO(PO_4)$	ICSD 237052[35]<br>ICSD 427591[35]	P4/n (85)	i L<br>i L	a LG<br>a LG
α_{II}-$VOPO_4$	$MoO(PO_4)$	ICSD 2889[55]	P4/n (85)	i L	i LG
$VOPO_4{\cdot}2H_2O$	n.a.	ICSD 200884[56]	P4/nmm (129)	a L	i L
α_I-$VOPO_4$	n.a.	ICSD 425552[57]	C2/m (12)	a L	i L
$VWPO_x$	ReO_3	ICSD 108651[58]	Pm-3m (221)	i LG [d]<br>i G [e]	-<br>-

[a] i = isotropic, a = anisotropic (original (strain) or modified (size) Stephens model), L = Lorentz, G = Gauss, LG = Voigt (i.e. convolution of Lorentz and Gauss). [b] Larger domain size, [c] smaller domain size component. [d] Crystalline, [e] nanocrystalline component.

The XRD patterns of $MoVO_x$ showed strongly "super-Lorentzian" reflections (i.e., sharp tips combined with very broad bases), indicating a rather wide domain size distribution. Accordingly, the Rietveld fits required the superposition of two $MoVO_x$ "phases" (with lattice parameters constrained to be identical), representing large and small domain sizes, to approximate the measured patterns reasonably well. In addition, the patterns exhibited strong preferred orientation effects with suppression of the 001 orientation. This observation is in line with the needle-like crystal morphology and demonstrates that the crystals were not agglomerated, as they responded readily to the gentle directing forces of sample preparation. In contrast, the isostructural $MoVTeNbO_x$ catalysts were highly crystalline, showed slightly anisotropic peak broadening, but lacked noticeable preferred orientation, thus indicating agglomeration of the needle-like crystals in random orientation. The diffraction patterns of $MnWO_4$ exhibited distinctly anisotropic peak broadening, which was fitted using a modified Stephens model[59] adapted for size broadening.[31] The reflection profiles of the $SmMnO_3$ perovskite and V_2O_5 were slightly anisotropic. The XRD patterns of crystalline VPP exhibited distinctly anisotropic peak broadening, which can be attributed to the presence of stacking faults.[60] Unfortunately, the effects of stacking faults on the diffraction pattern cannot be modeled satisfactory using conventional Rietveld methods. Hence, the best approximation available to us described the measured patterns only moderately well. "Amorphous VPP", which was actually found to contain some poorly crystalline hemihydrate $VOHPO_4{\cdot}½H_2O$ in addition to the amorphous component, crystallizes into $(VO)_2P_2O_7$ during the oxidation of the alkanes. The latter shows the same stacking fault problems as the originally crystalline VPP catalysts. The reflections of β- and α_{II}-$VOPO_4$ were isotropic, while the XRD patterns of α_{II}-$(V,W)OPO_4$ were found to be very inhomogeneous, likely due to an uneven distribution of Mo and V throughout the sample. Correspondingly, the patterns could only be approximated by using two strongly overlapping phases based on $(V_{0.74}W_{0.26})OPO_4$ and $(V_{0.9}W_{0.1})OPO_4$,[35] both with anisotropic peak broadening. The $VOPO_4{\cdot}2H_2O$ diffraction patters proved to be particularly troublesome, as the layered hydrate loses water upon mechanical pressing (preparation of defined sieve fraction for catalysis) or heating (during catalytic reaction), causing stacking faults and a general degradation of the crystal structure. Dehydrated samples contained more or less crystalline α_I-$VOPO_4$, which represents the completely dehydrated state of $VOPO_4{\cdot}2H_2O$, formed by topotactic deintercalation of water. When exposed to ambient conditions, however, the samples re-hydrated to $VOPO_4{\cdot}2H_2O$, again creating stacking faults which affected the α_I-$VOPO_4$ and $VOPO_4{\cdot}2H_2O$ patterns. Consequently, most samples were found to be $VOPO_4{\cdot}2H_2O/\alpha_I$-$VOPO_4$ mixtures during the XRD measurements, with strongly varying anisotropic peak broadening effects. While both $VOPO_4{\cdot}2H_2O$ and α_I-$VOPO_4$ were approximated using anisotropic peak broadening, the fits were typically not satisfactory. $VWPO_x$, which can be described with the formula $V_{0.167}W_{0.5}P_{0.333}O_x$, seems to have a ReO_3-type structure, which was approximated using a cubic WO_3 model with a mixed occupation of the metal site.

The diffraction patterns show discrete reflections on top of a diffuse background formed by very broad peaks, which seem to be centered around the crystalline reflections. Correspondingly, it was assumed that the diffuse peaks represent nanocrystalline material of similar composition as the crystalline $VWPO_x$ phase, and crystalline and diffuse component were both fitted with the same WO_3 type structure and coupled lattice parameters, but vastly different nominal domain sizes. Surprisingly, the nanocrystalline component did not crystallize further under catalytic conditions.
The broad diversity of structures and microstructural properties made a numerical assessment of the changes observed after the catalytic reactions difficult to impossible. The only numerical quantity, which is both accessible and comparable for all crystalline catalysts, is the relative change of the unit cell volumes, which is listed in Table S7 and visualized in Figure S16.
Table S8 summarizes whether or not fresh and spent catalysts were single phase according to XRD. In those cases in which a single phase fresh catalyst is not phase pure anymore after reaction, it can be inferred that the main phase is not stable against phase transformation under the conditions applied. In contrast, if the starting material is already a phase mixture, no conclusions about the main phase stability can be made from Table S8 alone. Thus, Table S9 gives information about whether or not the main phase of a catalyst seems to transform under catalytic conditions. Table S10 lists the secondary phases of mixed phase catalysts, although it needs to be kept in mind that some of the phase assignments are only tentative.
The observed diffraction peak profiles in the crystalline samples, and the models necessary to fit them, were too diverse (see Table S6) to allow a consistent numerical micro-structural characterization of all catalysts. However, an increase in peak broadening, independent from the actual nature of the defects, always indicates an increase in defectivity (i.e., smaller domain sizes, stronger mirco-strain, larger stacking fault concentration). Thus, we looked qualitatively at whether or not a catalytic reaction changed the peak broadening, with the results collected in Table S11.
Some general trends which can be deduced are the following: All vanadium phosphates $(V,W)OPO_4$ are phase mixtures, which undergo phase transformations during catalysis. The extent of phase changes depends on the reaction carried out. Generally, the α_{II} modification is partially transformed into the β modification. The highly crystalline, phase-pure materials $MoVO_x$, $MoVTeNbO_x$, $MnWO_4$, $SmMnO_3$, V_2O_5, and VPP are also characterized by high structural stability under most conditions. Only $MoVTeNbO_x$ and especially V_2O_5 exhibited some loss of phase integrity under propane oxidation conditions (Tables S9 and S10). Otherwise, there is no segregation of secondary phases after use, while changes in peak broadening and/or lattice parameters can be observed after the alkane oxidation reactions in many cases (Tables S7 and S11).

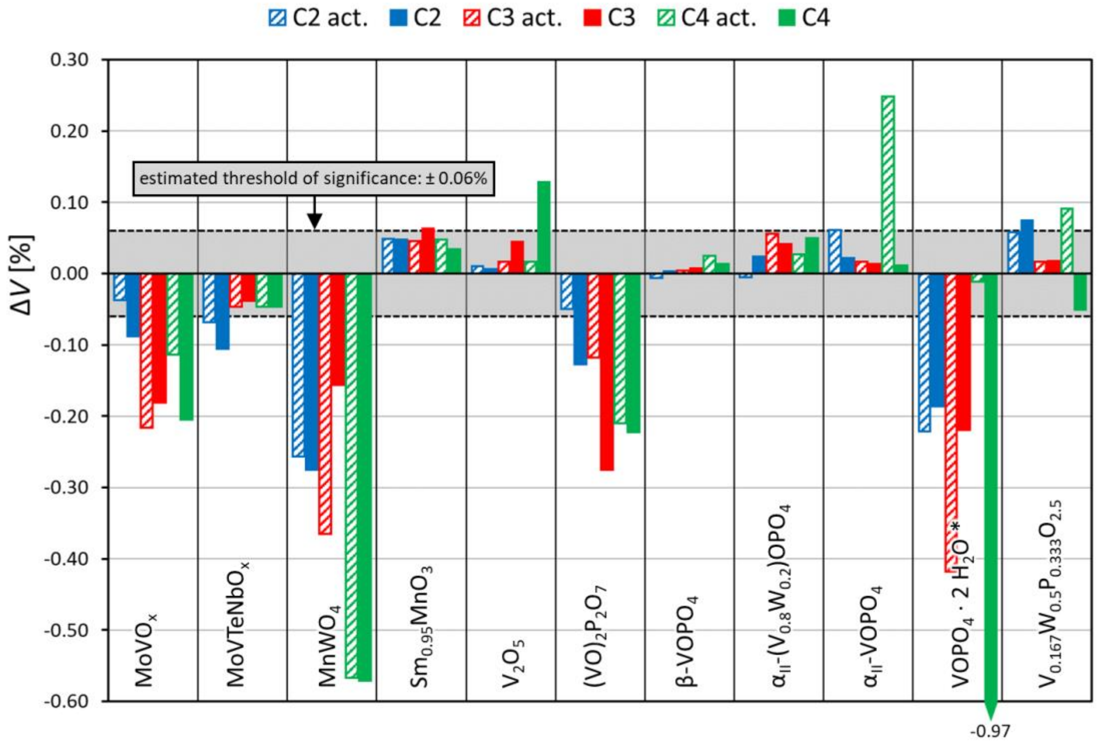

Figure S16. Visualization of relative unit cell volume changes from Table S7. Amorphous VPP is not included since the starting material has no defined unit cell. * In contrast to the remaining catalysts, where unit cell changes are likely related to changes in the oxidation state, the differences observed in $VOPO_4{\cdot}2H_2O$ are predominantly caused by the dynamic de-/re-hydration behavior and associated stacking faults.

Table S7. XRD Unit Cell Volumes of Fresh, Activated and Spent Samples and their Relative Changes. Significant Changes (> 0.06%) are Highlighted in Red.

catalyst	V_{fr} (Å^3) [a]	V_{act} (Å^3)	ΔV_{act} (%)	V_{spt} (Å^3)	ΔV_{spt} (%)
			ethane oxidation		
$MoVO_x$	2236.5	2235.6	-0.04	2234.5	-0.09
$MoVTeNbO_x$	2267.1	2265.6	-0.07	2264.7	-0.11
$MnWO_4$	139.1	138.7	-0.26	138.7	-0.27
$SmMnO_3$	234.0	234.2	0.05	234.2	0.05
V_2O_5	179.4	179.4	0.01	179.4	0.01
VPP	1235.1	1234.5	-0.05	1233.6	-0.13
amorphous VPP	n.a.	1234.5 [b]	n.a.	1232.7 [b]	n.a.
β-$VOPO_4$	333.2	333.1	-0.01	333.2	0.00
α_{II}-$VWOPO_4$	159.9	159.9	0.00	159.9	0.02
α_{II}-$VOPO_4$	160.1	160.2	0.06	160.2	0.02
$VOPO_4{\cdot}2H_2O$	286.4	285.8	-0.22	285.9	-0.19
$VWPO_x$	53.5	53.6	0.06	53.6	0.07
			propane oxidation		
$MoVO_x$	2236.5	2231.6	-0.22	2232.4	-0.18
$MoVTeNbO_x$	2267.1	2266.1	-0.05	2266.3	-0.04
$MnWO_4$	139.1	138.6	-0.36	138.9	-0.16
$SmMnO_3$	234.0	234.1	0.05	234.2	0.06
V_2O_5	179.4	179.4	0.02	179.5	0.04
VPP	1235.1	1233.7	-0.12	1231.7	-0.28
amorphous VPP	n.a.	1235.4 [b]	n.a.	1231.2 [b]	n.a.
β-$VOPO_4$	333.2	333.2	0.00	333.2	0.01
α_{II}-$VWOPO_4$	159.9	160.0	0.06	159.9	0.04
α_{II}-$VOPO_4$	160.1	160.1	0.02	160.1	0.01
$VOPO_4{\cdot}2H_2O$	286.4	285.2	-0.42	285.8	-0.22
$VWPO_x$	53.5	53.5	0.02	53.5	0.02
			n-butane oxidation		
$MoVO_x$	2236.5	2233.9	-0.11	2231.9	-0.20
$MoVTeNbO_x$	2267.1	2266.1	-0.05	2266.1	-0.05
$MnWO_4$	139.1	138.3	-0.57	138.3	-0.57
$SmMnO_3$	234.0	234.2	0.05	234.1	0.03
V_2O_5	179.4	179.4	0.02	179.6	0.13
VPP	1235.1	1232.6	-0.21	1232.4	-0.22
amorphous VPP	n.a.	1233.9 [b]	n.a.	1231.3 [b]	n.a.
β-$VOPO_4$	333.2	333.3	0.02	333.2	0.01
α_{II}-$VWOPO_4$	159.9	159.9	0.03	159.9	0.05
α_{II}-$VOPO_4$	160.1	160.5	0.25	160.1	0.01
$VOPO_4{\cdot}2H_2O$	286.4	286.4	-0.01	283.6	-0.97
$VWPO_x$	53.5	53.6	0.09	53.5	-0.05

[a] The cell volumes of the fresh samples are identical for the three reactions. [b] Refers to crystallized VPP.

Table S8. XRD Phase Purity of Fresh and Spent Catalysts. Deviations from the Main Trends are Highlighted in Red.

		ethane oxidation		propane oxidation		*n*-butane oxidation	
catalyst	fresh	activated	spent	activated	spent	activated	spent
$MoVO_x$	yes	yes	yes	yes	yes	yes	yes
$MoVTeNbO_x$	yes	yes	yes	yes	no	yes	yes
$MnWO_4$	yes	yes	yes	yes	yes	yes	yes
$SmMnO_3$	yes	yes	yes	yes	yes	yes	yes
V_2O_5	yes	yes	yes	yes	no	yes	yes
VPP	yes	yes	yes	yes	yes	yes	yes
amorphous VPP	no [a]	yes	yes	yes	yes	yes	yes
β-$VOPO_4$	no	no	no	no	no	no	no
α_{II}-$VWOPO_4$	no	no	no	no	no	no	no
α_{II}-$VOPO_4$	no	no	no	no	no	no	no
$VOPO_4 \cdot 2H_2O$	yes	(yes) [b]	(yes) [b]	(yes) [b]	(yes) [b]	no	no
$VWPO_x$	no	no	no	no	no	no	no

[a] Apart from the fact that the term "phase purity" is not applicable to amorphous material, the fresh sample contains traces of badly crystalline $VO(HPO_4) \cdot ½H_2O$. [b] Due to the dynamic behavior of $VOPO_4 \cdot 2H_2O$, α_I-$VOPO_4$ is regarded as its de-hydrated form rather than an impurity phase.

Table S9. Stability of the Primary Phase Against Phase Transformation. Deviations from the Main Trends are Highlighted in Red.

	ethane oxidation		propane oxidation		*n*-butane oxidation	
catalyst	activated	spent	activated	spent	activated	spent
$MoVO_x$	yes	yes	yes	yes	yes	yes
$MoVTeNbO_x$	yes	yes	yes	no	yes	yes
$MnWO_4$	yes	yes	yes	yes	yes	yes
$SmMnO_3$	yes	yes	yes	yes	yes	yes
V_2O_5	yes	yes	yes	no	yes	yes
VPP	yes	yes	yes	yes	yes	yes
amorphous VPP	(no) [a]	(no) [a]	(no) [a]	(no) [a]	(no) [a]	(no) [a]
β-$VOPO_4$	no	yes	yes	yes	yes	no
α_{II}-$VWOPO_4$	yes	yes	yes	yes	yes	no
α_{II}-$VOPO_4$	no	no	no	no	no	no
$VOPO_4 \cdot 2H_2O$	(no) [b]	(no) [b]	(no) [b]	(no) [b]	no	no
$VWPO_x$	yes	yes	yes	yes	yes	yes

[a] Conversion into crystalline VPP. [b] Dynamic interconversion with α_I-$VOPO_4$ due to de-/rehydration.

Table S10. Tentative Assignment of Impurity Phases Observed. Unususal Phases are Highlighted in Red.

catalyst	fresh [a]	activated	spent
		ethane oxidation	
$MoVO_x$	-	-	-
$MoVTeNbO_x$	-	-	-
$MnWO_4$	-	-	-
$SmMnO_3$	-	-	-
V_2O_5	-	-	-
VPP	-	-	-
amorphous VPP	$VO(HPO_4)\cdot$½H_2O	(crystalline VPP), β-$VOPO_4$	(crystalline VPP), β-$VOPO_4$
β-$VOPO_4$	V_2O_5, $VOPO_4\cdot 2H_2O$	V_2O_5, $VOPO_4\cdot 2H_2O$	V_2O_5
α_{II}-$VWOPO_4$	β-$VOPO_4$, $VOPO_4\cdot 2H_2O$	β-$VOPO_4$	β-$VOPO_4$
α_{II}-$VOPO_4$	β-$VOPO_4$, γ-$VOPO_4$, $VOPO_4\cdot 2H_2O$	β-$VOPO_4$, $VOPO_4\cdot 2H_2O$, second hydrate phase?	β-$VOPO_4$, α_I-$VOPO_4$, $VOPO_4\cdot 2H_2O$
$VOPO_4\cdot 2H_2O$	(α_I-$VOPO_4$)	(α_I-$VOPO_4$)	(α_I-$VOPO_4$)
$VWPO_x$	β-$VOPO_4$, $VOPO_4\cdot 2H_2O$, β''-$(VO)_2P_2O_7$	β-$VOPO_4$, $VOPO_4\cdot 2H_2O$	β-$VOPO_4$, β''-$(VO)_2P_2O_7$
		propane oxidation	
$MoVO_x$	-	-	-
$MoVTeNbO_x$	-	-	TeO_2, unknown phase
$MnWO_4$	-	-	-
$SmMnO_3$	-	-	-
V_2O_5	-	-	V_6O_{13}, unknown phase
VPP	-	-	-
amorphous VPP	$VO(HPO_4)\cdot$½H_2O	(crystalline VPP)	(crystalline VPP)
β-$VOPO_4$	V_2O_5, $VOPO_4\cdot 2H_2O$	V_2O_5	V_2O_5
α_{II}-$VWOPO_4$	β-$VOPO_4$, $VOPO_4\cdot 2H_2O$	β-$VOPO_4$	β-$VOPO_4$
α_{II}-$VOPO_4$	β-$VOPO_4$, γ-$VOPO_4$, $VOPO_4\cdot 2H_2O$	β-$VOPO_4$, β''-$(VO)_2P_2O_7$	β-$VOPO_4$, α_I-$VOPO_4$
$VOPO_4\cdot 2H_2O$	(α_I-$VOPO_4$)	(α_I-$VOPO_4$)	(α_I-$VOPO_4$)
$VWPO_x$	β-$VOPO_4$, $VOPO_4\cdot 2H_2O$, β''-$(VO)_2P_2O_7$	β-$VOPO_4$, α_{II}-$VOPO_4$, β''-$(VO)_2P_2O_7$	β-$VOPO_4$, α_{II}-$VOPO_4$
		n-butane oxidation	
$MoVO_x$	-	-	-
$MoVTeNbO_x$	-	-	-

$MnWO_4$	-	-	-
$SmMnO_3$	-	-	-
V_2O_5	-	-	-
VPP	-	-	-
amorphous VPP	$VO(HPO_4)\cdot½H_2O$	(crystalline VPP)	(crystalline VPP)
β-$VOPO_4$	V_2O_5, $VOPO_4\cdot2H_2O$	V_2O_5	V_2O_5, VPP
α_{II}-$VWOPO_4$	β-$VOPO_4$, $VOPO_4\cdot2H_2O$	β-$VOPO_4$, $VOPO_4\cdot2H_2O$	β-$VOPO_4$, c-WO_3
α_{II}-$VOPO_4$	β-$VOPO_4$, γ-$VOPO_4$, $VOPO_4\cdot2H_2O$	$VOPO_4\cdot2H_2O$, β-$VOPO_4$	β-$VOPO_4$, VPP, α_I-$VOPO_4$, $VOPO_4\cdot2H_2O$
$VOPO_4\cdot2H_2O$	(α_I-$VOPO_4$)	unknown phase	(α_I-$VOPO_4$), VPP
$VWPO_x$	β-$VOPO_4$, $VOPO_4\cdot2H_2O$, β''-$(VO)_2P_2O_7$	β-$VOPO_4$, $VOPO_4\cdot2H_2O$	β-$VOPO_4$, α_{II}-$VOPO_4$

[a] The fresh samples are identical for the three reactions.

Table S11. Stability of the Primary Phase XRD Peak Broadening. Deviations from the Main Trends are Highlighted in Red.

	ethane oxidation		propane oxidation		*n*-butane oxidation	
catalyst	activated	spent	activated	spent	activated	spent
$MoVO_x$	n.d. [a]	n.d. [a]	n.d. [a]	n.d. [a]	n.d. [a]	n.d. [a]
$MoVTeNbO_x$	yes	no	yes	yes	yes	yes
$MnWO_4$	no	no	no	no	no	no
$SmMnO_3$	yes	yes	yes	yes	yes	yes
V_2O_5	yes	yes	yes	yes	yes	no
VPP	no	no	no	no	no	no
amorphous VPP	n.a. [b]	n.a. [b]	n.a. [b]	n.a. [b]	n.a. [b]	n.a. [b]
β-$VOPO_4$	yes	yes	yes	yes	yes	yes
α_{II}-$VWOPO_4$	no	yes	yes	yes	no	yes
α_{II}-$VOPO_4$	yes	yes	yes	yes	n.d. [c]	yes
$VOPO_4\cdot2H_2O$	no [d]	no [d]	no [d]	no [d]	no [d]	no [d]
$VWPO_x$	yes	yes	yes	yes	yes	yes

[a] Due to the broad domain size distribution combined with preferred orientation effects, the peak broadening cannot be assessed reliably. [b] Not applicable due to amorphous stating material. [c] Not determined since main phase is mostly converted to $VOPO_4\cdot2H_2O$. [d] Anisotropic peak broadening heavily influenced by stacking faults, caused by de-/re-hydration dynamics.

8. Analysis of Surface Area and Texture of Fresh and Activated Catalysts

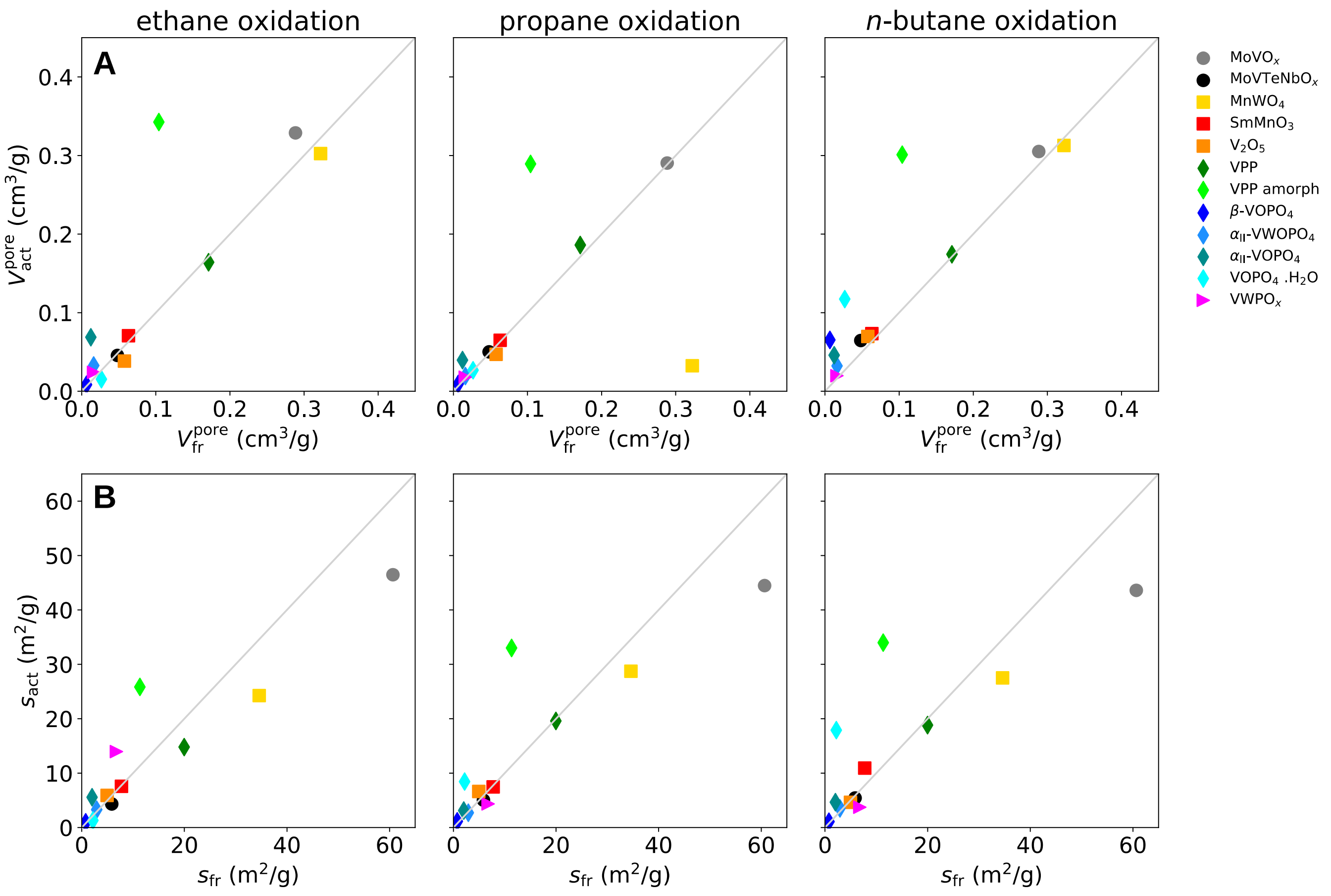

Figure S17. (A) pore volume and (B) specific surface area measured by N_2 adsorption.

9. Surface Analysis by Photoelectron Spectroscopy

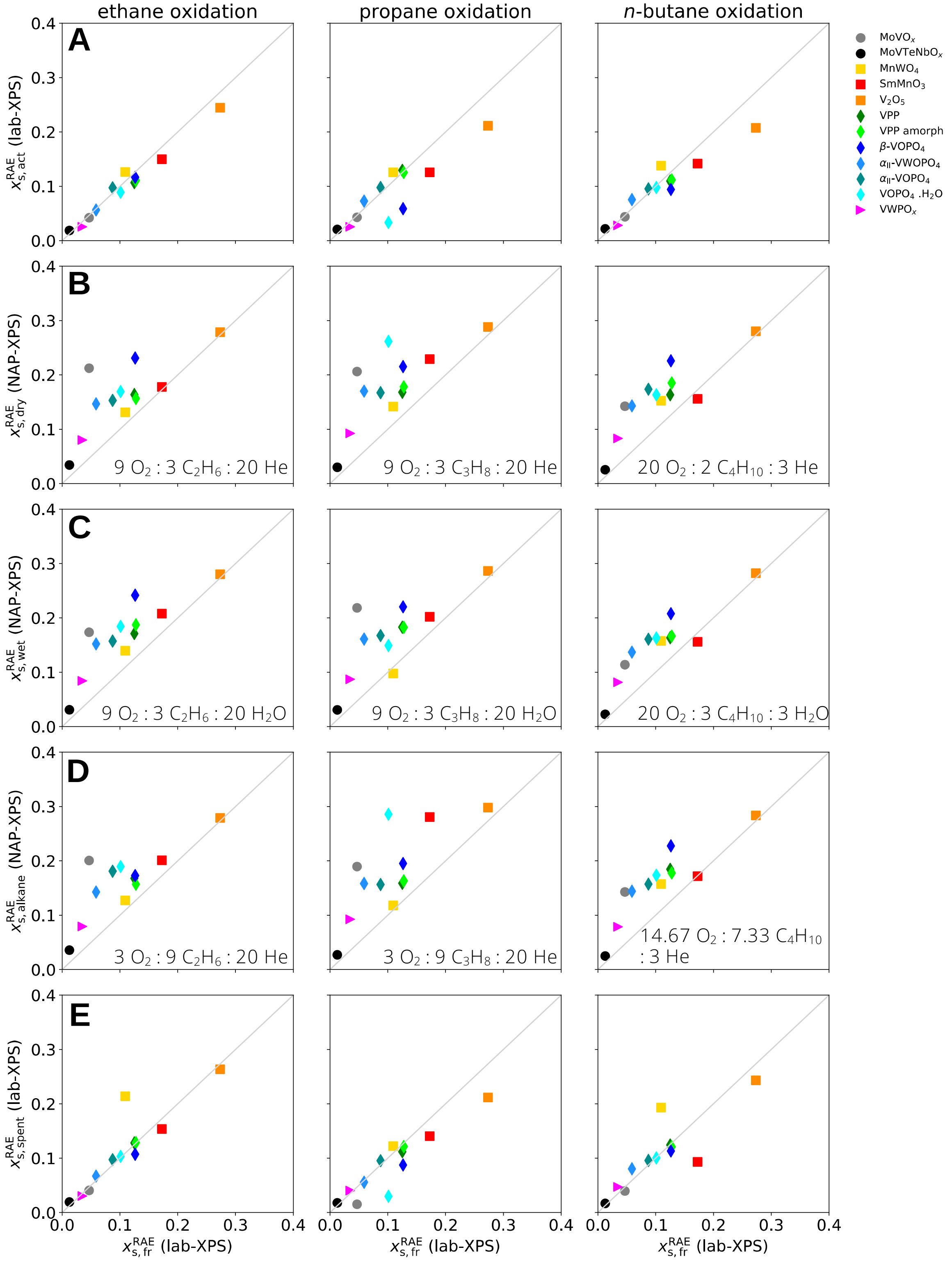

Figure S18. Redox-active element (RAE, V or Mn) surface content measured by XPS. The surface RAE content of (A) the activated materials (B-D) the materials under reaction conditions and (E) the spent materials (shown in the *y*-axes) is different from that of the fresh catalyst (shown in the *x*-axes). The feeds applied in NAP-XPS are indicated in the plots. The NAP-XPS measurements were performed at the reference temperature (T_{ref}).

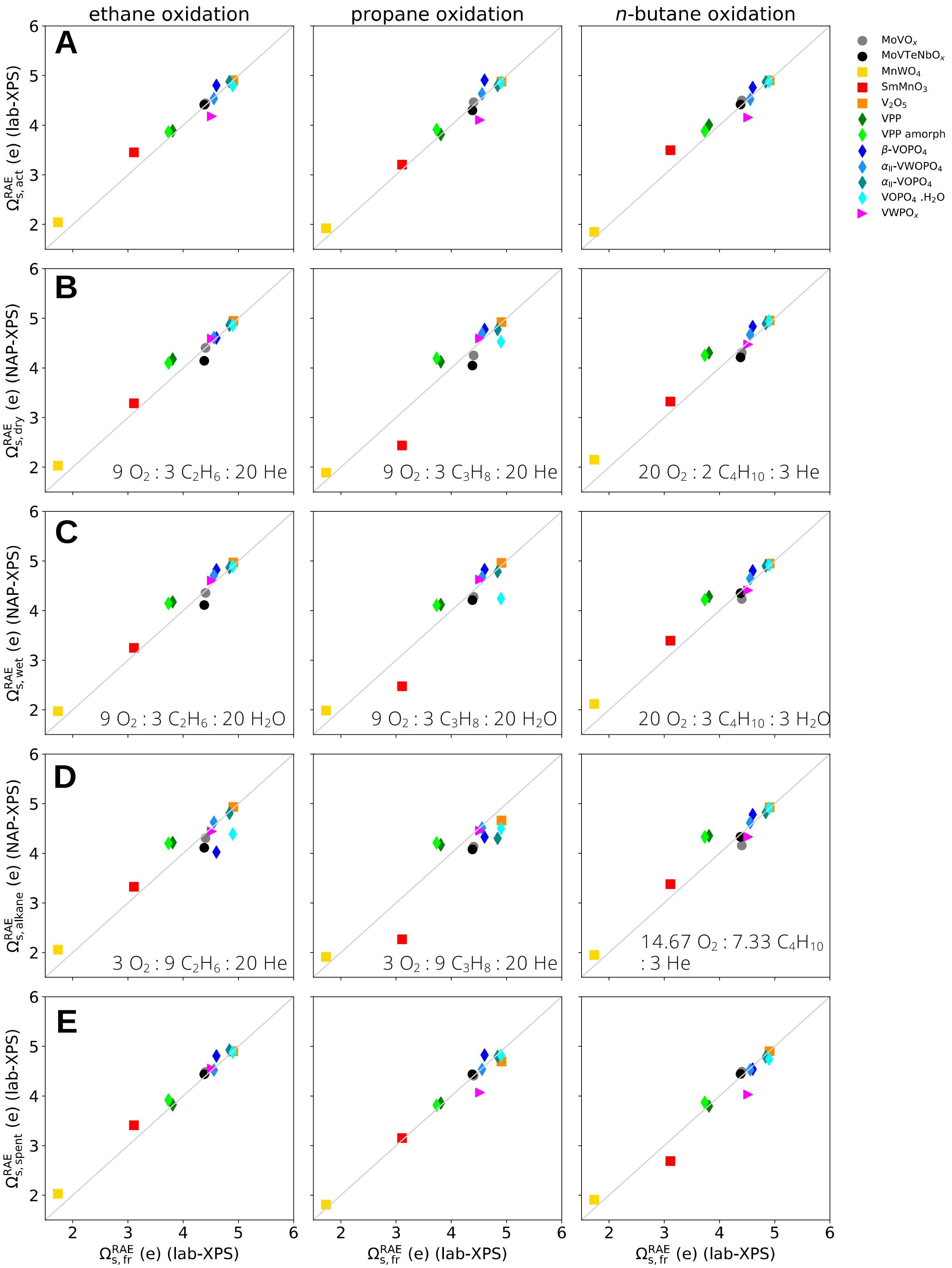

Figure S19. Redox-active element (RAE, V or Mn) oxidation state measured by XPS. The RAE oxidation states of (A) the activated materials (B-D) the materials under reaction conditions and (E) the spent materials (shown in the *y*-axes) is different from that of the fresh catalyst (shown in the *x*-axes). The feeds applied in NAP-XPS are indicated in the plots. The NAP-XPS measurements were performed at the reference temperature (T_{ref}).

Table S12. Consistent Oxidation States Observed among all Materials.

element	oxidation state
O	-2
Mo	5.95-6.00
Te	4
Nb	5
P	5
W	6

Table S13. Oxidation States of the Redox Active Elements (RAE, V or Mn) Measured by XPS and NAP-XPS. These Values are Graphically Shown in Fig. S19.

	XPS			NAP-XPS		
catalyst	$\Omega_{s,fr}^{RAE}$[a] (e)	$x_{s,act}^{RAE}$ (e)	$\Omega_{s,spt}^{RAE}$ (e)	$\Omega_{s,dry}^{RAE}$ (e)	$\Omega_{s,wet}^{RAE}$ (e)	$\Omega_{s,alkane}^{RAE}$ (e)
			ethane oxidation			
$MoVO_X$	4.41	4.44	4.48	4.40	4.36	4.31
$MoVTeNbO_X$	4.38	4.41	4.44	4.14	4.11	4.11
$MnWO_4$	1.73	2.04	2.03	2.03	1.97	2.06
$SmMnO_3$	3.11	3.45	3.41	3.29	3.25	3.33
V_2O_5	4.91	4.91	4.90	4.95	4.97	4.93
VPP	3.81	3.89	3.82	4.18	4.18	4.22
amorphous VPP	3.73	3.86	3.92	4.10	4.14	4.20
β-$VOPO_4$	4.6	4.80	4.81	4.60	4.82	4.02
α_{II}-$VWOPO_4$	4.56	4.54	4.52	4.61	4.71	4.63
α_{II}-$VOPO_4$	4.84	4.88	4.93	4.86	4.87	4.81
$VOPO_4 \cdot 2H_2O$	4.9	4.80	4.88	4.86	4.89	4.39
$VWPO_x$	4.52	4.18	4.55	4.59	4.60	4.44
			propane oxidation			
$MoVO_X$	4.41	4.46	4.41	4.25	4.28	4.13
$MoVTeNbO_X$	4.38	4.30	4.44	4.05	4.21	4.08
$MnWO_4$	1.73	1.92	1.81	1.89	1.99	1.91
$SmMnO_3$	3.11	3.21	3.15	2.44	2.48	2.27
V_2O_5	4.91	4.88	4.70	4.92	4.96	4.66
VPP	3.81	3.81	3.86	4.12	4.12	4.17
amorphous VPP	3.73	3.91	3.81	4.20	4.11	4.21
β-$VOPO_4$	4.6	4.91	4.83	4.78	4.83	4.33
α_{II}-$VWOPO_4$	4.56	4.63	4.54	4.69	4.67	4.51
α_{II}-$VOPO_4$	4.84	4.80	4.8	4.77	4.78	4.30
$VOPO_4 \cdot 2H_2O$	4.9	4.86	4.82	4.53	4.24	4.50
$VWPO_x$	4.52	4.11	4.07	4.59	4.62	4.45
			n-butane oxidation			
$MoVO_X$	4.41	4.50	4.49	4.31	4.23	4.16
$MoVTeNbO_X$	4.38	4.42	4.44	4.21	4.35	4.33
$MnWO_4$	1.73	1.85	1.91	2.15	2.12	1.95
$SmMnO_3$	3.11	3.50	2.69	3.32	3.39	3.38

V_2O_5	4.91	4.90	4.9	4.95	4.95	4.93
VPP	3.81	4.01	3.8	4.3	4.28	4.35
amorphous VPP	3.73	3.88	3.87	4.25	4.22	4.33
β-$VOPO_4$	4.6	4.76	4.54	4.83	4.8	4.78
α_{II}-$VWOPO_4$	4.56	4.52	4.53	4.67	4.65	4.62
α_{II}-$VOPO_4$	4.84	4.87	4.79	4.89	4.90	4.82
$VOPO_4 \cdot 2H_2O$	4.9	4.88	4.74	4.95	4.92	4.91
$VWPO_x$	4.52	4.16	4.03	4.47	4.41	4.33

[a] The oxidation state of the RAE on the fresh samples are identical for the three reactions.

10. Further Detailed Results of the SISSO Analysis

Table S14. Models Identified for Alkane in Ethane, Ppropane and *n*-Butane Oxidation Reactions by the SISSO Approach Using Different Sets of Orimary Features.

primary features	(q, D)[a]	training RMSE	CV-RMSE	model expression
all	(3,1)	4.34	6.19	$X_{\text{alkane}}^{(\text{SISSO})} = c_0^X + c_1^X \mathrm{u}_{\text{m,fr}}^{\text{O}_2} \frac{\left\| \left| x_{\text{s,fr}}^{\text{RAE}} - x_{\text{s,fr}}^{\text{C}} \right| - \left| x_{\text{s,fr}}^{\text{RAE}} - x_{\text{s,wet}}^{\text{RAE}} \right| \right\|}{\Omega_{\text{s,spt}}^{\text{RAE}} \Omega_{\text{s,act}}^{\text{RAE}} \Omega_{\text{s,wet}}^{\text{RAE}}}$
exluding *in situ* (NAP-XPS) data	(1,2)	5.01	10.87	$X_{\text{alkane}}^{(\text{SISSO})} = c_0^X + c_1^X \frac{x_{\text{s,fr}}^{\text{C}}}{x_{\text{s,act}}^{\text{RAE}}} + c_2^X \mathrm{u}_{\text{m,fr}}^{\text{O}_2} x_{\text{s,act}}^{\text{RAE}}$
only fresh-catalyst data	(2,1)	7.93	10.43	$X_{\text{alkane}}^{(\text{SISSO})} = c_0^X + c_1^X \frac{\mathrm{u}_{\text{s,fr}}^{\text{O}_2} V_{\text{fr}}^{\text{pore}}}{x_{\text{s,fr}}^{\text{C}} - x_{\text{s,fr}}^{\text{O}}}$

[a] Optimal model complexity with respect to predictability identified by leave-one-material-out cross-validation. q and D correspond to the depth of the symbolic-regression tree and to the descriptor dimension, respectively.

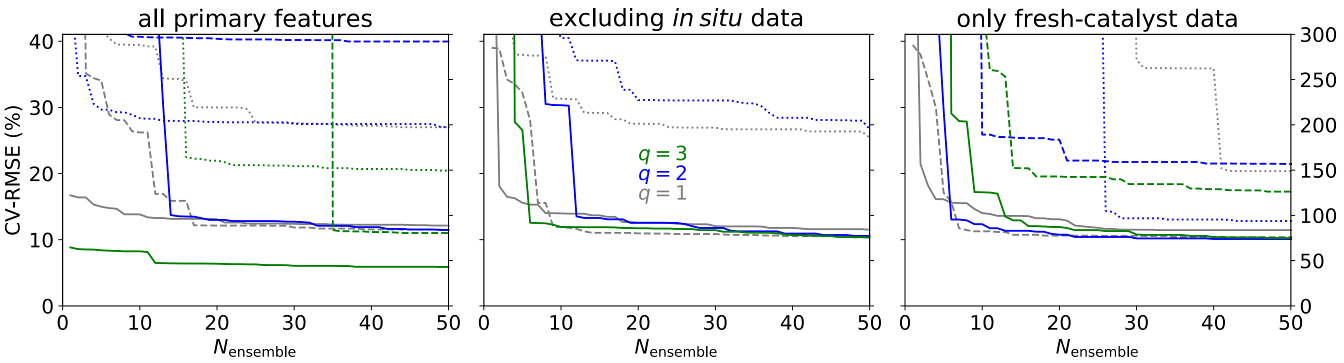

Figure S20. Average CV-RMSE dependence on the number of top-ranked descriptors analyzed (N_{ensemble}) for X^{alkane} with different sets of primary features. The solid, dashed and dotted lines correspond, respectively, to 1-, 2-, and 3-D models.

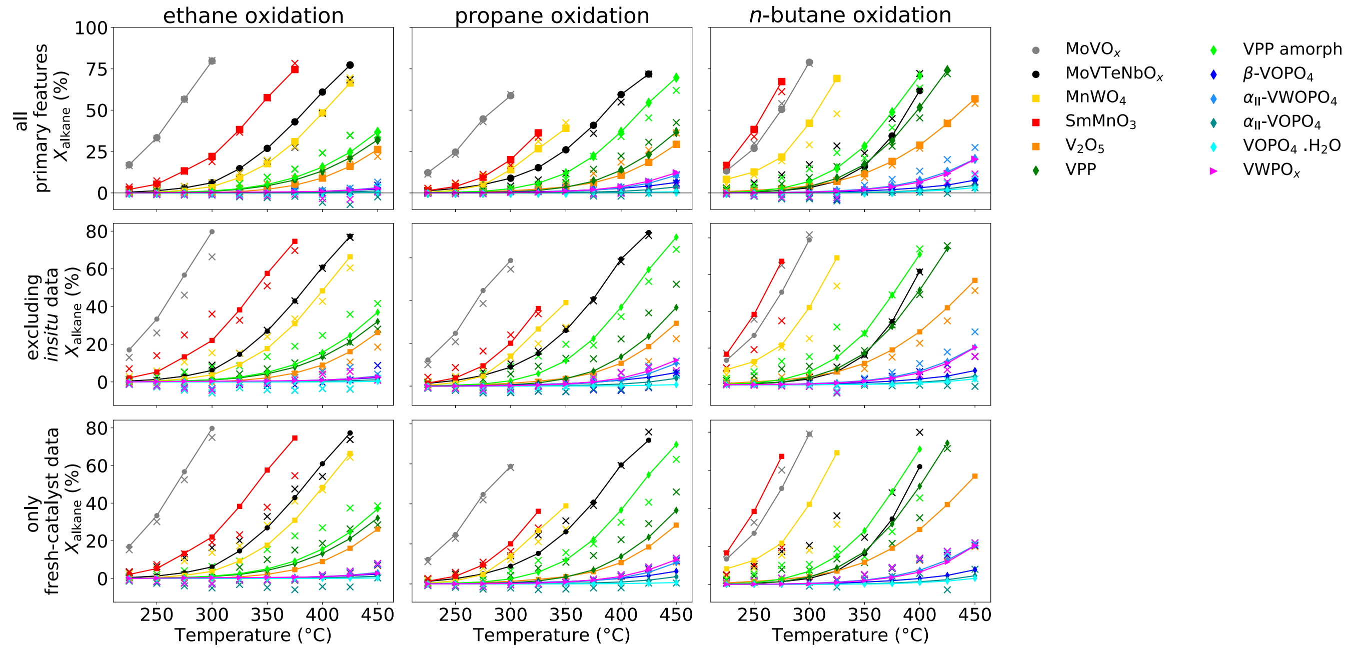

Figure S21. Measured values for alkane conversion (X^{alkane}) as well as the fits of the best models identified using different sets of primary features.

11. References

(1) Katou, T.; Vitry, D.; Ueda, W. Structure dependency of Mo-V-O-based complex oxide catalysts in the oxidations of hydrocarbons. *Catalysis Today* **2004**, *91-92* (1), 237-240.
(2) Konya, T.; Katou, T.; Murayama, T.; Ishikawa, S.; Sadakane, M.; Buttrey, D.; Ueda, W. An orthorhombic Mo3VOx catalyst most active for oxidative dehydrogenation of ethane among related complex metal oxides. *Catalysis Science & Technology* **2013**, *3* (2), 380-387, 10.1039/C2CY20444D. DOI: 10.1039/C2CY20444D.
(3) Wernbacher, A. M.; Kube, P.; Hävecker, M.; Schlögl, R.; Trunschke, A. Electronic and Dielectric Properties of MoV-Oxide (M1 Phase) under Alkane Oxidation Conditions. *The Journal of Physical Chemistry C* **2019**, *123* (21), 13269-13282. DOI: 10.1021/acs.jpcc.9b01273.
(4) Melzer, D.; Mestl, G.; Wanninger, K.; Jentys, A.; Sanchez-Sanchez, M.; Lercher, J. A. On the Promoting Effects of Te and Nb in the Activity and Selectivity of M1 MoV-Oxides for Ethane Oxidative Dehydrogenation. *Topics in Catalysis* **2020**, *63* (19), 1754-1764. DOI: 10.1007/s11244-020-01304-0.
(5) Ishikawa, S.; Yi, X.; Murayama, T.; Ueda, W. Catalysis field in orthorhombic Mo3VOx oxide catalyst for the selective oxidation of ethane, propane and acrolein. *Catalysis Today* **2014**, *238* (0), 35-40. DOI: http://dx.doi.org/10.1016/j.cattod.2013.12.054.
(6) Trunschke, A.; Noack, J.; Trojanov, S.; Girgsdies, F.; Lunkenbein, T.; Pfeifer, V.; Hävecker, M.; Kube, P.; Sprung, C.; Rosowski, F.; et al. The Impact of the Bulk Structure on Surface Dynamics of Complex Mo–V-based Oxide Catalysts. *ACS Catalysis* **2017**, *7* (4), 3061-3071. DOI: 10.1021/acscatal.7b00130.
(7) López Nieto, J. M.; Botella, P.; Vázquez, M. I.; Dejoz, A. The selective oxidative dehydrogenation of ethane over hydrothermally synthesised MoVTeNb catalysts. *Chemical Communications* **2002**, (17), 1906-1907, 10.1039/B204037A. DOI: 10.1039/B204037A.
(8) Lopez Nieto, J. M.; Solsona, B.; Concepcion, P.; Ivars, F.; Dejoz, A.; Vazquez, M. I. Reaction products and pathways in the selective oxidation of C2-C4 alkanes on MoVTeNb mixed oxide catalysts. *Catalysis Today* **2010**, *157* (1-4), 291-296, doi: 10.1016/j.cattod.2010.01.046.
(9) Lin, M.; Desai, T. B.; Kaiser, F. W.; Klugherz, P. D. Reaction pathways in the selective oxidation of propane over a mixed metal oxide catalyst. *Catalysis Today* **2000**, *61* (1-4), 223-229.
(10) Heine, C.; Hävecker, M.; Sanchez-Sanchez, M.; Trunschke, A.; Schlögl, R.; Eichelbaum, M. Work Function, Band Bending, and Microwave Conductivity Studies on the Selective Alkane Oxidation Catalyst MoVTeNb Oxide (Orthorhombic M1 Phase) under Operation Conditions. *The Journal of Physical Chemistry C* **2013**, *117* (51), 26988-26997. DOI: 10.1021/jp409601h (acccessed 2014/01/05).
(11) Li, X.; Teschner, D.; Streibel, V.; Lunkenbein, T.; Masliuk, L.; Fu, T.; Wang, Y.; Jones, T.; Seitz, F.; Girgsdies, F.; et al. How to control selectivity in alkane oxidation? *Chemical Science* **2019**, *10* (8), 2429-2443, 10.1039/C8SC04641G. DOI: 10.1039/C8SC04641G.
(12) Koch, G.; Hävecker, M.; Teschner, D.; Carey, S. J.; Wang, Y.; Kube, P.; Hetaba, W.; Lunkenbein, T.; Auffermann, G.; Timpe, O.; et al. Surface Conditions That Constrain Alkane Oxidation on Perovskites. *ACS Catalysis* **2020**, *10* (13), 7007-7020. DOI: 10.1021/acscatal.0c01289.
(13) Oyama, S. T.; Somorjai, G. A. Effect of structure in selective oxide catalysis: oxidation reactions of ethanol and ethane on vanadium oxide. *The Journal of Physical Chemistry* **1990**, *94* (12), 5022-5028. DOI: 10.1021/j100375a048.
(14) Ai, M. Oxidation of propane to acrylic acid on vanadium pentoxide-phosphorus pentoxide-based catalysts. *Journal of Catalysis* **1986**, *101* (2), 389-395.
(15) Chaar, M. A.; Patel, D.; Kung, H. H. Selective oxidative dehydrogenation of propane over V-Mg-O catalysts. *Journal of Catalysis* **1988**, *109* (2), 463-467. DOI: https://doi.org/10.1016/0021-9517(88)90226-6.
(16) Chaar, M. A.; Patel, D.; Kung, M. C.; Kung, H. H. Selective oxidative dehydrogenation of butane over V-Mg-O catalysts. *Journal of Catalysis* **1987**, *105* (2), 483-498. DOI: https://doi.org/10.1016/0021-9517(87)90076-5.
(17) Merzouki, M.; Taouk, B.; Tessier, L.; Bordes, E.; Courtine, P. Correlation Between Catalytic and Structural Properties of Modified Molybdenum and Vanadium Oxides in the Oxidation of Ethane in Acetic Acid or Ethylene. In *Studies in Surface Science and Catalysis*, Guczi, L., Solymosi, F., TÉTÉNyi, P. Eds.; Vol. 75; Elsevier, 1993; pp 753-764.
(18) Ivars-Barceló, F.; Hutchings, G. J.; Bartley, J. K.; Taylor, S. H.; Sutter, P.; Amorós, P.; Sanchis, R.; Solsona, B. Relationship between bulk phase, near surface and outermost atomic layer of VPO catalysts and their catalytic performance in the oxidative dehydrogenation of ethane. *Journal of Catalysis* **2017**, *354*, 236-249. DOI: https://doi.org/10.1016/j.jcat.2017.08.020.
(19) Landi, G.; Lisi, L.; Russo, G. Oxidation of propane and propylene to acrylic acid over vanadyl pyrophosphate. *Journal of Molecular Catalysis A: Chemical* **2005**, *239* (1-2), 172-179.
(20) Hutchings, G. J.; Higgins, R. Effect of Promoters on the Selective Oxidation ofn-Butane with Vanadium-Phosphorus Oxide Catalysts. *Journal of Catalysis* **1996**, *162* (2), 153-168. DOI: https://doi.org/10.1006/jcat.1996.0273.

(21) Zhanglin, Y.; Forissier, M.; Sneeden, R. P.; Vedrine, J. C.; Volta, J. C. On the Mechanism of n-Butane Oxidation to Maleic Anhydride on VPO Catalysts: I. A Kinetics Study on a VPO Catalyst as Compared to VPO Reference Phases. *Journal of Catalysis* **1994**, *145* (2), 256-266. DOI: https://doi.org/10.1006/jcat.1994.1033.
(22) Schulz, C.; Pohl, F.; Driess, M.; Glaum, R.; Rosowski, F.; Frank, B. Selective Oxidation of n-Butane over Vanadium Phosphate Based Catalysts: Reaction Network and Kinetic Analysis. *Industrial & Engineering Chemistry Research* **2019**, *58* (7), 2492-2502. DOI: 10.1021/acs.iecr.8b04328.
(23) Tomoaki, S.; Toshio, O.; Makoto, M. Preparation of Vanadium–Phosphorus Mixed Oxide (P/V=1) Catalysts and Their Application to Oxidation of Butane to Maleic Anhydride. *Bulletin of the Chemical Society of Japan* **1985**, *58* (8), 2163-2171. DOI: 10.1246/bcsj.58.2163.
(24) Schulz, C.; Roy, S. C.; Wittich, K.; d'Alnoncourt, R. N.; Linke, S.; Strempel, V. E.; Frank, B.; Glaum, R.; Rosowski, F. αII-(V1-xWx)OPO4 catalysts for the selective oxidation of n-butane to maleic anhydride. *Catalysis Today* **2019**, *333*, 113-119. DOI: https://doi.org/10.1016/j.cattod.2018.05.040.
(25) Eichelbaum, M.; Glaum, R.; Hävecker, M.; Wittich, K.; Heine, C.; Schwarz, H.; Dobner, C.-K.; Welker-Nieuwoudt, C.; Trunschke, A.; Schlögl, R. Towards Physical Descriptors of Active and Selective Catalysts for the Oxidation of n-Butane to Maleic Anhydride. *ChemCatChem* **2013**, *5* (8), 2318-2329. DOI: 10.1002/cctc.201200953.
(26) Griesel, L.; Bartley, J. K.; Wells, R. P. K.; Hutchings, G. J. Preparation of vanadium phosphate catalysts from VOPO4·2H2O: effect of VOPO4·2H2O preparation on catalyst performance. *Journal of Molecular Catalysis A: Chemical* **2004**, *220* (1), 113-119. DOI: https://doi.org/10.1016/j.molcata.2004.02.027.
(27) Welker-Nieuwoudt, C. A.; Rosowksi, F.; Goebel, M.; Glaum, R.; Subrata, C. R.; Hautier, G.; Waroquiers, D.; Naumann d'Alnoncourt, R.; Strempel, V.; Linke, S. Wolframphosphate der ReO3 - Strukturfamilie. Germany 2016.
(28) Trunschke, A.; Noack, J.; Trojanov, S.; Girgsdies, F.; Lunkenbein, T.; Pfeifer, V.; Hävecker, M.; Kube, P.; Sprung, C.; Rosowski, F.; et al. The Impact of the Bulk Structure on Surface Dynamics of Complex Mo–V-based Oxide Catalysts. *ACS Catalysis* **2017**, *7*, 3061-3071. DOI: 10.1021/acscatal.7b00130.
(29) Trunschke, A.; Bellini, G.; Boniface, M.; Carey, S. J.; Dong J.; Erdem, E.; Foppa, L.; Frandsen, W.; Geske, M.; Ghiringhelli, L. M.; et al. Towards experimental handbooks in catalysis. *Topics in Catalysis* **2020**, *63*, 1683–1699. DOI: 10.1007/s11244-020-01380-2.
(30) Sanchez Sanchez, M.; Girgsdies, F.; Jastak, M.; Kube, P.; Schlögl, R.; Trunschke, A. Aiding the Self-Assembly of Supramolecular Polyoxometalates under Hydrothermal Conditions To Give Precursors of Complex Functional Oxides. *Angewandte Chemie International Edition* **2012**, *51* (29), 7194-7197. DOI: 10.1002/anie.201200746.
(31) Li, X.; Lunkenbein, T.; Krohnert, J.; Pfeifer, V.; Girgsdies, F.; Rosowski, F.; Schlogl, R.; Trunschke, A. Hydrothermal synthesis of bi-functional nanostructured manganese tungstate catalysts for selective oxidation. *Faraday Discussions* **2016**, *188* (0), 99-113, 10.1039/C5FD00191A. DOI: 10.1039/C5FD00191A.
(32) Li, X.; Lunkenbein, T.; Pfeifer, V.; Jastak, M.; Nielsen, P. K.; Girgsdies, F.; Knop-Gericke, A.; Rosowski, F.; Schlögl, R.; Trunschke, A. Selective Alkane Oxidation by Manganese Oxide: Site Isolation of MnOx Chains at the Surface of MnWO4 Nanorods. *Angewandte Chemie International Edition* **2016**, *55* (12), 4092-4096. DOI: 10.1002/anie.201510201.
(33) Koch, G.; Hävecker, M.; Kube, P.; Tarasov, A.; Schlögl, R.; Trunschke, A. The Influence of the Chemical Potential on Defects and Function of Perovskites in Catalysis. *Frontiers in Chemistry* **2021**, *9* (775), Original Research. DOI: 10.3389/fchem.2021.746229.
(34) Weiguny, J.; S. Storck; M. Duda; Dobner, C. Catalyst and method for producing maleic anhydride. Germany 2003.
(35) Roy, S. C.; Glaum, R.; Abdullin, D.; Schiemann, O.; Quang Bac, N.; Lii, K.-H. Solid Solution Formation between Vanadium(V) and -Tungs-ten(V) Oxide Phosphate. *Zeitschrift für anorganische und allgemeine Chemie* **2014**, *640* (10), 1876-1885, https://doi.org/10.1002/zaac.201400160. DOI: https://doi.org/10.1002/zaac.201400160 (acccessed 2022/09/12).
(36) C. Welker-Nieuwoudt; F. Rosowski; M. Goebel; R. Glaum; C.R. Subrata; G. Hautier; D. Waroquiers; R. Naumann d'Alnoncourt; V.E. Strempel; Linke, S. Wolframphosphate der ReO3-Strukturfamilie. Germany 2016.
(37) Shimoda, T.; Okuhara, T.; Misono, M. Preparation of Vanadium–Phosphorus Mixed Oxide (P/V=1) Catalysts and Their Application to Oxidation of Butane to Maleic Anhydride. *Bulletin of the Chemical Society of Japan* **1985**, *58* (8), 2163-2171. DOI: 10.1246/bcsj.58.2163 (acccessed 2022/09/12).
(38) Koyano, G.; Okuhara, T.; Misono, M. Structural Changes of Surface Layer of Vanadyl Pyrophosphate Catalysts by Oxidation−Reduction and Their Relationships with Selective Oxidation of n-Butane. *Journal of the American Chemical Society* **1998**, *120* (4), 767-774. DOI: 10.1021/ja964437b.
(39) Yeh, J. J.; Lindau, I. Atomic subshell photoionization cross sections and asymmetry parameters: $1 \leq Z \leq 103$. *At. Data Nucl. Data Tables* **1985**, *32* (1), 1-155. DOI: https://doi.org/10.1016/0092-640X(85)90016-6.
(40) Tanuma, S.; Powell, C. J.; Penn, D. R. Calculations of Electron Inelastic Mean Free Paths (IMFPS). IV. Evaluation of Calculated IMFPs and of the Predictive IMFP Formula TPP-2 for Electron Energies between 50 and 2000 eV. *Surface and interface analysis* **1993**, *20* (1), 77-89.

(41) Sawhney, K. J. S.; Senf, F.; Scheer, M.; Schäfers, F.; Bahrdt, J.; Gaupp, A.; Gudat, W. A novel undulator-based PGM beamline for circularly polarised synchrotron radiation at BESSY II. *Nuclear Instruments and Methods in Physics Research Section A: Accelerators, Spectrometers, Detectors and Associated Equipment* **1997**, *390* (3), 395-402. DOI: https://doi.org/10.1016/S0168-9002(97)00402-6.
(42) Knop-Gericke, A.; Kleimenov, E.; Hävecker, M.; Blume, R.; Teschner, D.; Zafeiratos, S.; Schlögl, R.; Bukhtiyarov, V. I.; Kaichev, V. V.; Prosvirin, I. P.; et al. Chapter 4 X-Ray Photoelectron Spectroscopy for Investigation of Heterogeneous Catalytic Processes. In *Advances in Catalysis*, Bruce, C. G., Helmut, K. Eds.; Vol. Volume 52; Academic Press, 2009; pp 213-272.
(43) Salmeron, M.; Schlögl, R. Ambient pressure photoelectron spectroscopy: A new tool for surface science and nanotechnology. *Surface Science Reports* **2008**, *63* (4), 169-199, doi: DOI: 10.1016/j.surfrep.2008.01.001.
(44) Tanuma, S.; Powell, C. J.; Penn, D. R. Calculations of electorn inelastic mean free paths. II. Data for 27 elements over the 50–2000 eV range. *Surf. Interface Anal.* **1991**, *17* (13), 911-926. DOI: doi:10.1002/sia.740171304.
(45) Tanuma, S.; Powell, C. J.; Penn, D. R. Calculation of electron inelastic mean free paths (IMFPs) VII. Reliability of the TPP-2M IMFP predictive equation. *Surf. Interface Anal.* **2003**, *35* (3), 268-275. DOI: doi:10.1002/sia.1526.
(46) Walton, J.; Wincott, P.; Fairley, N.; Carrick, A. *Peak Fitting with CasaXPS*; Accolyte Science, 2010.
(47) Purcell, T. A. R.; Scheffler, M.; Carbogno, C.; Ghiringhelli, L. M. SISSO++: A C++ Implementation of the Sure-Independence Screening and Sparsifying Operator Approach. *Journal of Open Source Software* **2022**, *7 (71)*, 3960. DOI: 10.21105/joss.03960.
(48) Foppa, L.; Ghiringhelli, L. M.; Girgsdies, F.; Hashagen, M.; Kube, P.; Hävecker, M.; Carey, S. J.; Tarasov, A.; Kraus, P.; Rosowski, F.; et al. Materials genes of heterogeneous catalysis from clean experiments and artificial intelligence. *MRS Bulletin* **2021**, *46*, 1016-1026. DOI: 10.1557/s43577-021-00165-6.
(49) DeSanto, P.; Buttrey, D. J.; Grasselli, R. K.; Lugmair, C. G.; Volpe, A. F.; Toby, B. H.; Vogt, T. Structural aspects of the M1 and M2 phases in MoVNbTeO propane ammoxidation catalysts. **2004**, *219* (3), 152-165. DOI: doi:10.1524/zkri.219.3.152.29091 (acccessed 2022-09-12).
(50) Weitzel, H. Kristallstrukturverfeinerung von Wolframiten und Columbiten. **1976**, *144* (1-6), 238-258. DOI: doi:10.1524/zkri.1976.144.16.238 (acccessed 2022-09-12).
(51) O'Flynn, D.; Tomy, C. V.; Lees, M. R.; Daoud-Aladine, A.; Balakrishnan, G. Multiferroic properties and magnetic structure of Sm${}_{1\ensuremath{-}x}$Y${}_{x}$MnO${}_{3}$. *Physical Review B* **2011**, *83* (17), 174426. DOI: 10.1103/PhysRevB.83.174426.
(52) Enjalbert, R.; Galy, J. A refinement of the structure of V2O5. *Acta Crystallographica Section C* **1986**, *42* (11), 1467-1469. DOI: doi:10.1107/S0108270186091825.
(53) Hiroi, Z.; Azuma, M.; Fujishiro, Y.; Saito, T.; Takano, M.; Izumi, F.; Kamiyama, T.; Ikeda, T. Structural Study of the Quantum-Spin Chain Compound (VO)2P2O7. *Journal of Solid State Chemistry* **1999**, *146* (2), 369-379. DOI: https://doi.org/10.1006/jssc.1999.8364.
(54) He, G.; Huq, A.; Kan, W. H.; Manthiram, A. β-NaVOPO4 Obtained by a Low-Temperature Synthesis Process: A New 3.3 V Cathode for Sodium-Ion Batteries. *Chemistry of Materials* **2016**, *28* (5), 1503-1512. DOI: 10.1021/acs.chemmater.5b04992.
(55) Jordan, B.; Calvo, C. Crystal Structure of α-VPO5. *Canadian Journal of Chemistry* **1973**, *51* (16), 2621-2625. DOI: 10.1139/v73-396 (acccessed 2022/09/13).
(56) Tietze, H. R. The crystal and molecular structure of oxovanadium(V) orthophosphate dihydrate, VOPO₄,2H₂O. *Australian Journal of Chemistry* **1981**, *34* (10), 2035-2038.
(57) Gautier, R.; Gautier, R.; Hernandez, O.; Audebrand, N.; Bataille, T.; Roiland, C.; Elkaïm, E.; Le Pollès, L.; Furet, E.; Le Fur, E. DFT-assisted structure determination of α1- and α2-VOPO4: new insights into the understanding of the catalytic performances of vanadium phosphates. *Dalton Transactions* **2013**, *42* (22), 8124-8131, 10.1039/C3DT50217A. DOI: 10.1039/C3DT50217A.
(58) *Izvestiya Akademii Nauk SSSR, Neorganicheskie Materialy* **1973**, *9*, 718-720.
(59) Stephens, P. W. Phenomenological model of anisotropic peak broadening in powder diffraction. *Journal of Applied Crystallography* **1999**, *32* (2), 281-289, https://doi.org/10.1107/S0021889898006001. DOI: https://doi.org/10.1107/S0021889898006001 (acccessed 2022/09/13).
(60) Nguyen, P. T.; Sleight, A. W.; Roberts, N.; Warren, W. W. Modeling of Extended Defects in the Vanadium Phosphate Catalyst for Butane Oxidation, (VO)2P2O7. *Journal of Solid State Chemistry* **1996**, *122* (2), 259-265. DOI: https://doi.org/10.1006/jssc.1996.0111.

12. Link to Handbook and Raw Data

https://ac.archive.fhi.mpg.de/P51850
